# Genome-wide mapping of common and rare variant effects on adiposity across childhood

**DOI:** 10.1038/s41588-026-02772-y

**Published:** 2026-09-25

**Authors:** Katherine A. Kentistou, Jonas Sundfjord, Roya Karimi, Lena R. Kaisinger, Robin J. Hofmeister, Nicolas Fragoso-Bargas, Adina E. Lupu, Yajie Zhao, John A. Tadross, Lukas Steuernagel, Georgina K. C. Dowsett, Sam Lockhart, Jens C. Brüning, Jimmy Liu, Adrian Cortes, Yancy Lo, Jonathan Davitte, Lieven Clement, Alexandra Havdahl, Ole A. Andreassen, Eirik Bratland, Brian Y. H. Lam, Stephen O’Rahilly, Giles S. H. Yeo, Pål R. Njølstad, Zoltán Kutalik, Felix R. Day, Marc Vaudel, John R. B. Perry, Ken K. Ong, Stefan Johansson

**Affiliations:** 1 IMS Epidemiology, Institute of Metabolic Science, University of Cambridge, Cambridge, UK; 2 Mohn Center for Diabetes Precision Medicine, Department of Clinical Science, University of Bergen, Bergen, Norway; 3 Department of Computational Biology, University of Lausanne, Lausanne, Switzerland; 4 Medical Research Council Metabolic Diseases Unit, Institute of Metabolic Science, Metabolic Research Laboratories, University of Cambridge, Cambridge, UK; 5 Cambridge Genomics Laboratory, Cambridge University Hospitals NHS Foundation Trust, Cambridge, UK; 6 Department of Histopathology, Cambridge University Hospitals NHS Foundation Trust, Cambridge, UK; 7 Department of Neuronal Control of Metabolism, Max Planck Institute for Metabolism Research, Cologne, Germany; 8 Wellcome-Wolfson Institute for Experimental Medicine, Queen’s University Belfast, Belfast, UK; 9 Human Genetics and Genomics, GSK, Collegeville, PA USA; 10 Human Genetics and Genomics, GSK, Stevenage, UK; 11 Department of Mathematics, Computer Science and Statistics, Ghent University, Ghent, Belgium; 12 PsychGen Centre for Genetic Epidemiology and Mental Health, Division of Public Health and Prevention, Norwegian Institute of Public Health, Oslo, Norway; 13 Research Department, Lovisenberg Diaconal Hospital, Oslo, Norway; 14 Promenta Research Centre, Department of Psychology, University of Oslo, Oslo, Norway; 15 Center for Precision Psychiatry, Faculty of Medicine, University of Oslo, Oslo, Norway; 16 Division of Mental Health and Addiction, Oslo University Hospital, Oslo, Norway; 17 Department of Pediatrics and Adolescent Medicine, Haukeland University Hospital, Bergen, Norway; 18 University Center for Primary Care and Public Health, Unisanté, University of Lausanne, Lausanne, Switzerland; 19 Computational Biology Unit, Department of Informatics, University of Bergen, Bergen, Norway; 20 Department of Genetics and Bioinformatics, Norwegian Institute of Public Health, Bergen, Norway

**Keywords:** Genome-wide association studies, Obesity

## Abstract

Our understanding of the genetic architecture of obesity is primarily based on studies of adults, with sparser data from childhood. Here, we conducted age-stratified genetic association studies against objectively measured (*n* = 62,276) or recalled childhood adiposity-related traits (*n*_eff_ = 599,924), identifying 624 common variants associated with childhood adiposity, with one-third having no concordant association with adult body mass index. Signals linked to the leptin–melanocortin pathway (*BSX*, *GNAS*, *LEPR* and *PCSK1*) and incretin signaling (*GIPR* and *GLP1R*) showed childhood-specific effects on adiposity. Single-nucleus RNA sequencing data identified childhood-specific adiposity-regulating cell populations in the arcuate nucleus and mammillary bodies, indicating neurocircuits that regulate adiposity specifically during childhood. Finally, sequencing in 479,615 individuals uncovered rare protein-coding variation in *ADCY3*, *CALCR*, *MC4R*, *MRAP2*, *POMC* and *MYH13*, exhibiting stronger associations in childhood than in adults. Our findings suggest that childhood provides a more sensitive window for the study of key endocrine and neuropeptide pathways that regulate energy balance.

## Abstract (web summary)

Genome-wide analyses identify common and rare variants associated with childhood adiposity, including rare protein-coding variants in *ADCY3*, *CALCR*, *MC4R*, *MRAP2*, *POMC* and *MYH13* that exhibit stronger effects in children than in adults.

## Main

The prevalence of child and adolescent obesity has more than quadrupled over the past three decades^1^. These trends are concerning, as childhood obesity is linked to many comorbidities^2,3^ and usually tracks into adulthood, when it causes long-term adverse health outcomes^4^. Obesity has a pronounced genetic component, with an estimated heritability of 40–90% (ref. ^5^). The largest genome-wide association study (GWAS) for body mass index (BMI) to date identified over 900 genetic loci in over 700,000 adults^6^ and implicated central nervous system pathways controlling the hedonic aspects of food intake as a major driver of obesity^7,8^.

Most GWAS loci identified for adult BMI are also associated with obesity in children^9–11^, but there is growing evidence that some genetic factors influence BMI differently between young age and adulthood^12–16^. Observed links between early growth and later adiposity^17^, and also growing interest in efforts to prevent overweight and obesity starting from very early in life^18^, motivate studying the genetics of early childhood adiposity development. However, the largest GWAS analyses to date on measured childhood BMI are considerably smaller than among adults, as they included discovery samples of 39,620 and 84,804 children, pooled across ages from 2 to 10 years, and found 25 and 39 significant loci, respectively^10,19^. As an alternative to measured BMI, self-recalled body size at age 10 (SAC10) from 453,169 UK Biobank participants^14^ identified 295 loci and showed high genetic correlation with measured childhood BMI, offering a substantial increase in power compared to conventional measures. Nevertheless, most studies lack specificity about the timing of association and do not address the full allelic spectrum.

To meet these challenges, we performed discovery GWAS analyses for childhood BMI at unprecedented age granularity and sample size, covering 11 timepoints from age 6 weeks to 8 years in up to 62,276 children with longitudinal data from the Norwegian Mother, Father and Child Cohort study (MoBa). We next use a genomic structural equation modeling (gSEM) approach^20^ to combine different childhood adiposity-related traits: MoBa childhood BMI at age 8, UK Biobank SAC10 and a GWAS meta-analysis of age of menarche (AAM), a trait tightly linked with early life adiposity^21,22^. This yielded an effective GWAS sample size of 599,924 individuals. We characterize the age-specific trajectories of association of identified signals on BMI, analyze single-cell sequence data to compare signal enrichment in specific hypothalamic cell types and use whole-genome sequencing (WGS) data to identify childhood-specific rare variant associations. This approach uncovers genetic determinants of childhood adiposity at an unprecedented depth.

## Results

### Novel signals for early life BMI

**Fig. 1:**
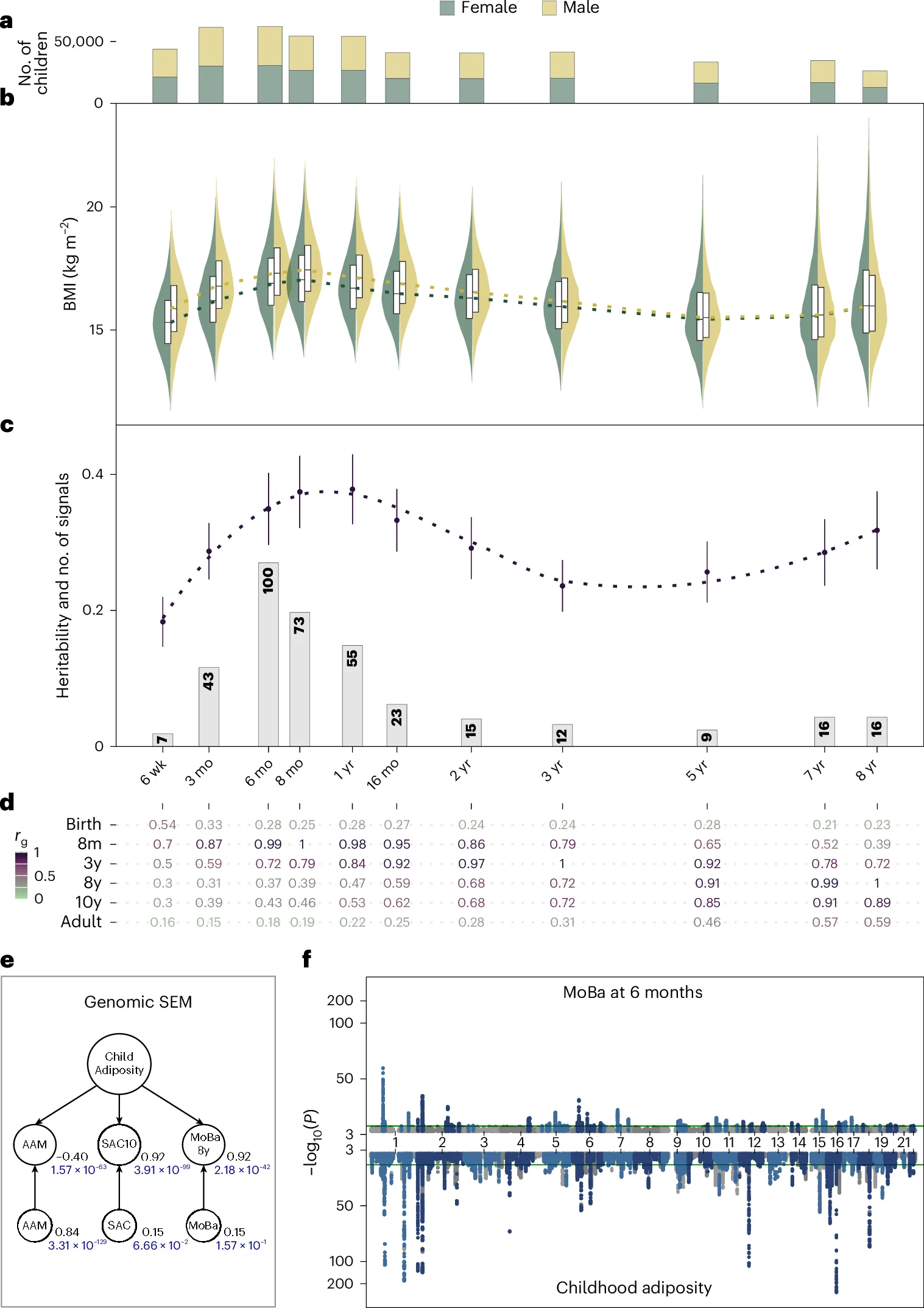
Genetic discovery of variants influencing BMI throughout childhood. **a**, Number of children included in the GWAS at each timepoint. **b**, Violin plots of BMI distributions at each timepoint with boxplots displaying first, second and third quartiles. Sample sizes for each timepoint are provided in **a** and Supplementary Data 1. **c**, SNP-based heritability estimates for each timepoint and its interpolation (dashed line). Error bars display 95% CIs. Columns with numbers indicate the number of independent signals at each timepoint. **d**, Genetic correlations for BMI at birth, 8 months, 3 years and 8 years in MoBa, recalled adiposity at 10 years (SAC) in UK Biobank (*n* = 444,345) and adult BMI^6^. **e**, gSEM common factor loadings (higher circles) and residual variance (lower circles) for each contributing factor. **f**, Miami plot of the two-sided genome-wide *P* values from the MoBa GWAS at 6 months (upper plot) and the gSEM child adiposity common factor GWAS (lower plot; *n*_eff_ = 599,924). Genome-wide significant variants are highlighted in blue.

GWAS was performed in up to 62,276 children (49.1% girls) from MoBa for age-standardized and sex-standardized BMI at 11 timepoints from ages 6 weeks to 8 years (Fig. 1a–c and Supplementary Data 1–3). We identified between seven and 100 conditionally independent genome-wide significant (*P* < 5 × 10^−8^) single-nucleotide polymorphisms (SNPs) at each timepoint (total of 369 associations). SNP-based heritability estimates varied with age, mirroring the changes in absolute BMI, increasing after birth to the ‘adiposity peak’ at age 1 year, then decreasing until the age at ‘adiposity rebound’. There was only modest genetic correlation between BMI in infancy and childhood (8 months–8 years: *r*_*g*_ = 0.39) and even lower with adult BMI (8 months–adult: *r*_*g*_ = 0.19) (Fig. 1d).

The 369 SNPs identified across the 11 timepoints mapped into 152 independent signals, of which 106 also meet the stricter *P* < 1 × 10^−8^, accounting for the multiple timepoints tested (see Methods and Supplementary Data 4). The 152 signals represent a major increase from previous results in a subsample of MoBa^13^ (*n* = 36 from the same ages) and in the largest independent GWAS of childhood BMI^10^ (*n* = 25), with 94% of the 116 novel loci identified for BMI before age 5 years and no signal showing evidence of a sex interaction (all *P*_interaction_ > 0.01).

**Extended Data Fig. 1:**
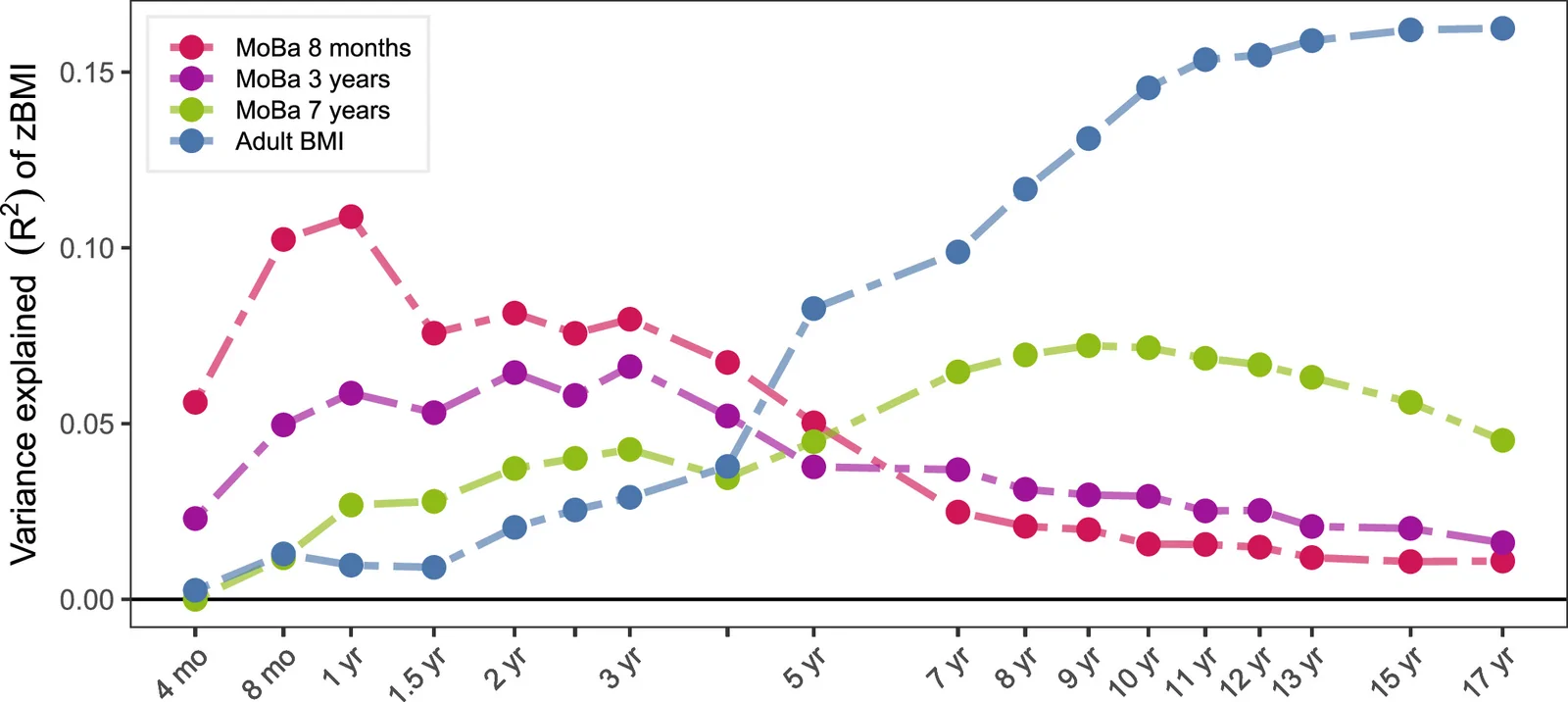
Performance of time-stratified genomic PGSs (PGS_GW) based on MoBa discovery to predict childhood BMI in ALSPAC. For comparison, performance of a PGS based on adult BMI GWAS data^6^ is also shown. Further data can be found in Supplementary Data 6.

We constructed two polygenic scores (PGS) at each timepoint, comprising the 152 independent SNPs identified in MoBa and a genome-wide score. We then compared their performance to corresponding PGSs for adult BMI^6^ in the independent Avon Longitudinal Study of Parents and Children (ALSPAC) cohort (Supplementary Data 5–7). Both childhood scores explained a greater proportion of the variance than the adult BMI-based counterpart for all timepoints below age 5 years (Extended Data Fig. 1).

### Childhood adiposity-related phenotypes increase discovery

Although the heritability of BMI increased again after age 3 years, we identified relatively few signals for BMI at ages 7–8 years because there are fewer measurements at these timepoints in MoBa (Fig. 1a). To increase power, we constructed a gSEM model by combining data from MoBa childhood BMI at age 8 (MoBa8), SAC10 and a recently published GWAS for AAM^21^ to estimate a ‘childhood adiposity’ common factor. Almost half of all genetic variants associated with AAM act through early life weight-related mechanisms. This analysis passed quality control and validation metrics and yielded an effective sample size of 599,924 (Supplementary Note). SAC10 and MoBa8 showed strong factor loadings on the childhood adiposity common factor (both 0.92; Fig. 1e), while AAM had a moderate negative loading (−0.40).

**Extended Data Fig. 2:**
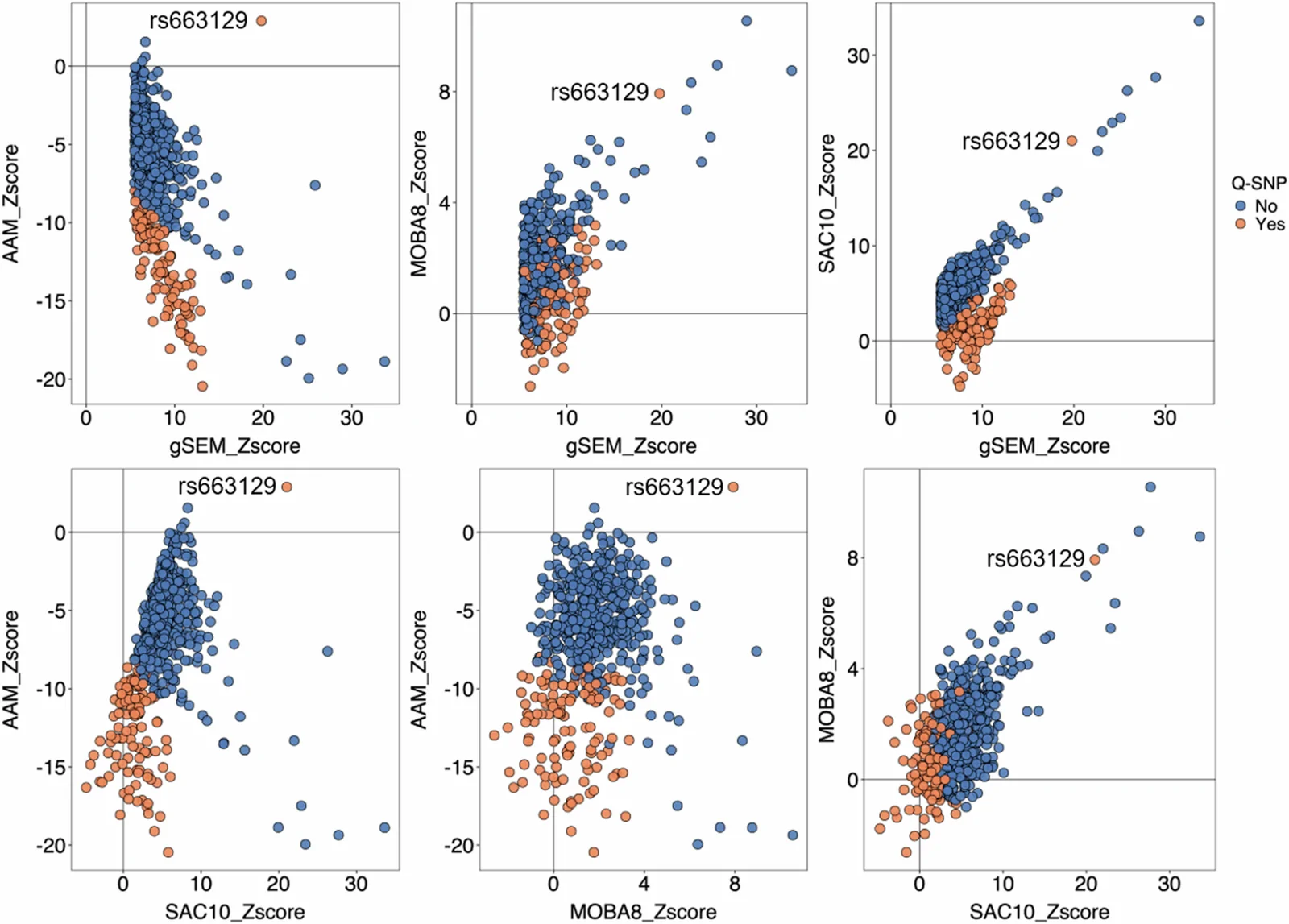
Scatterplots comparing common factor and individual trait associations (*Z*-scores) for the genomic SEM GWAS signals. Heterogeneous signals (*P*_Q_ < 5 × 10^−8^, two-tailed) are highlighted in orange; all of these except for rs663129 (which maps to *MC4R*) were excluded in subsequent genetic analyses. AAM, age at menarche; MOBA8, childhood BMI age 8; SAC10, comparative body size age 10. Further data can be found in Supplementary Data 8.

We performed a GWAS for this childhood adiposity common factor and identified 526 independent (*r*^2^ < 0.05) genome-wide significant signals (*P* < 5 × 10^−8^), after excluding 117 heterogeneous signals (Supplementary Data 8) with disproportionately large effects on AAM and relatively weak association with MoBa8 or SAC10 (Extended Data Fig. 2). The childhood adiposity common factor showed high genetic correlation with measured childhood BMI in the independent EGG sample^10^ (*r*_g_ = 0.85). The 526 GWAS signals showed high directional concordance with both measured childhood BMI (472 out of 520, 91%; *P* = 3.6 × 10^−89^) and adult BMI (488 out of 526, 93%; *P* = 1.2 × 10^−100^), and none showed convincing evidence of a sex-specific effect (Supplementary Data 9).

**Fig. 2:**
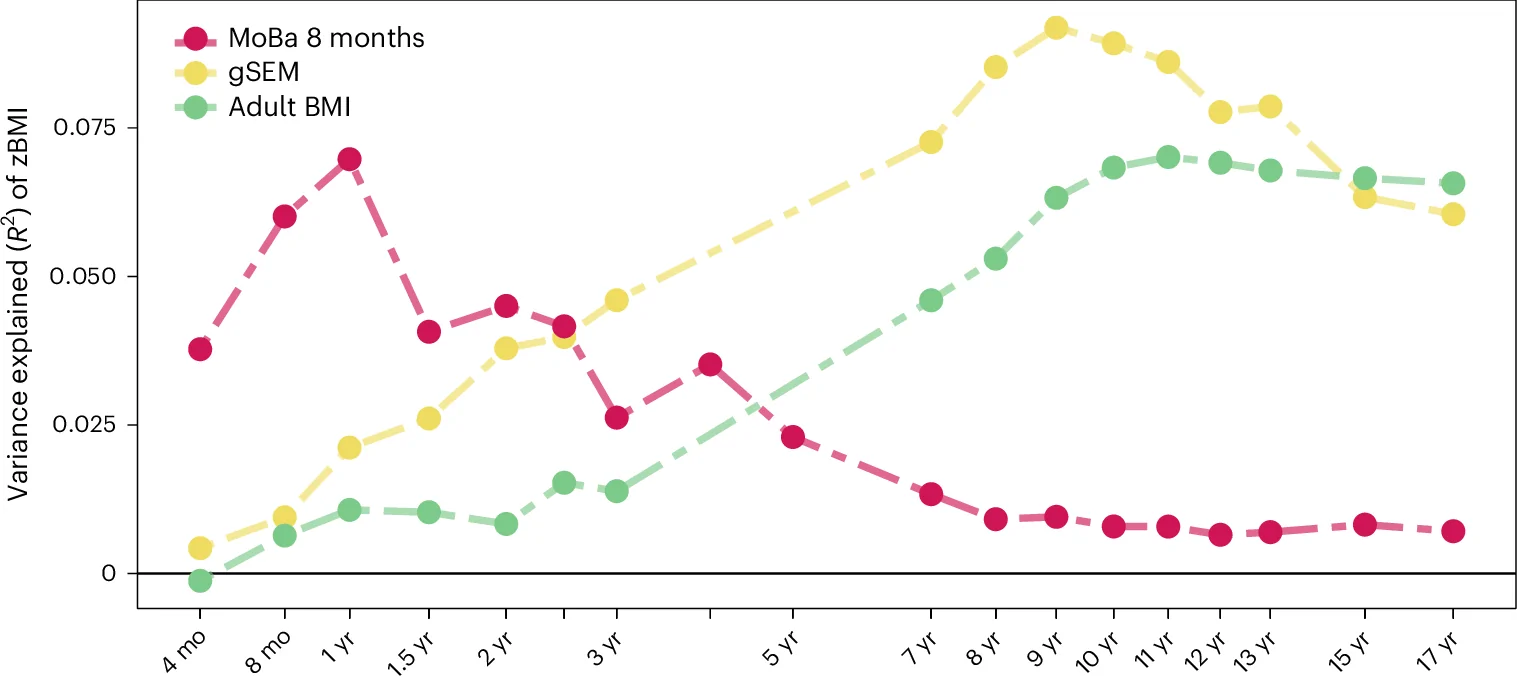
Performance of the childhood adiposity common factor PGS (gSEM) and an infancy-stratified PGS (MoBa8) on BMI across childhood in ALSPAC. Performance is indicated by the variance explained in standardized BMI (zBMI) at each age. Performance of an adult BMI PGS^6^ is also plotted for comparison. Underlying data are provided in Supplementary Data 5 and 7, and details of the ALSPAC sample, including sample size for specific time points, can be found in Supplementary Data 1.

**Extended Data Fig. 3:**
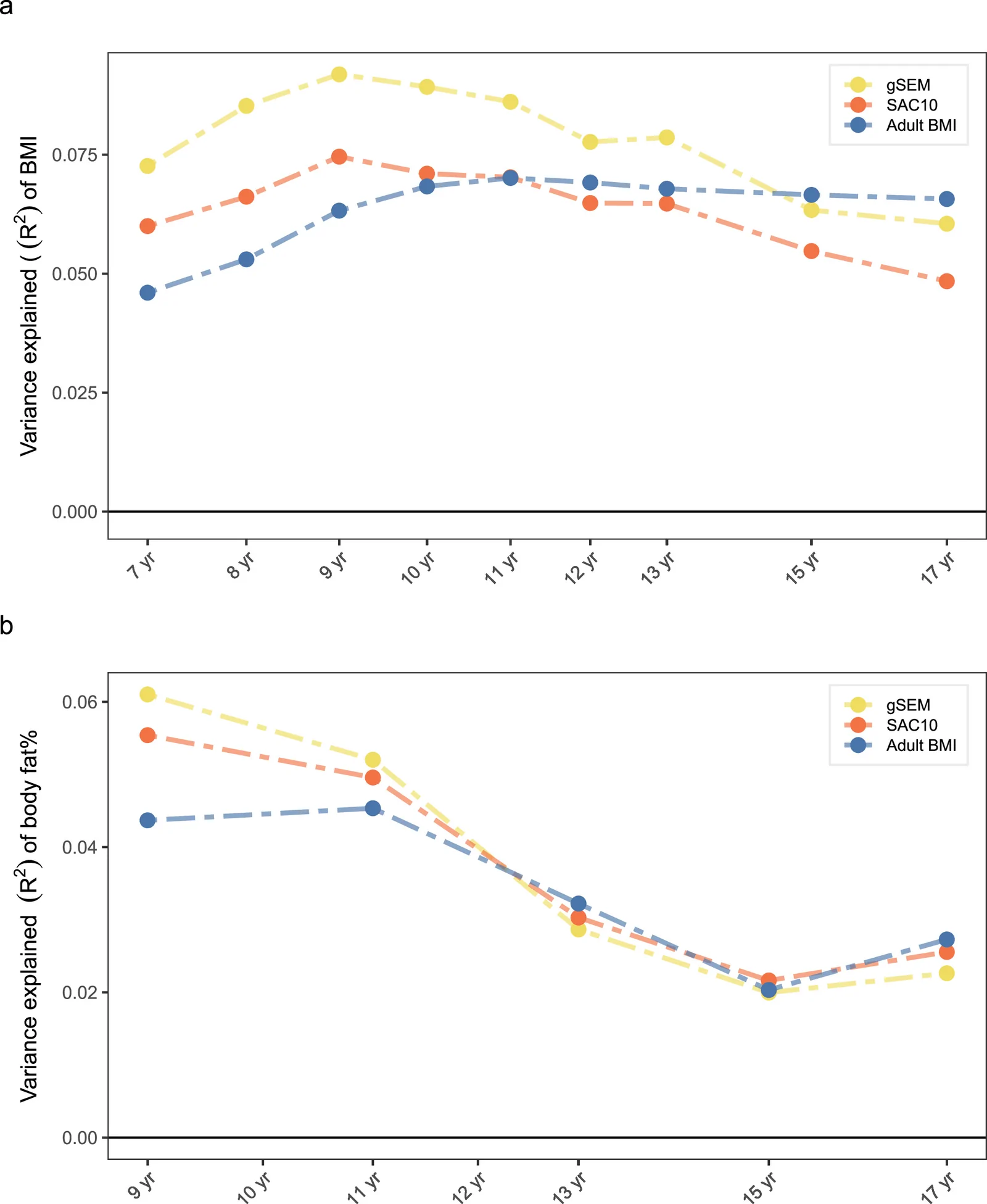
Performance of the childhood adiposity common factor PGS (‘gSEM’) to predict childhood BMI and childhood fat percentage in ALSPAC. **a**, Childhood BMI. **b**, Childhood fat percentage. For comparison, performance of a PGS based on recalled size at age 10 (SAC10), and a PGS based on adult BMI GWAS data^6^ are also shown. Further data can be found in Supplementary Data 7.

A PGS comprising the 526 signals for the childhood adiposity common factor explained up to 9.2% of childhood BMI (peak age 9) (Fig. 2) and up to 6.1% of childhood body fat percentage (peak age 9) in ALSPAC (Supplementary Data 7). This performance was higher than similar PGSs based on SAC10 alone or adult BMI on childhood BMI and childhood body fat percentage (Extended Data Fig. 3).

### Two main trajectories of genetic effect

We combined the 152 cross-sectional signals from MoBa with the 526 signals for the childhood adiposity common factor, yielding 624 independent signals (Supplementary Data 9), of which only 7% (*n* = 44) were previously discovered in the two largest studies of measured childhood BMI^10,16^. We then performed unsupervised hard clustering of their trajectories of association with BMI at 12 MoBa timepoints (including at birth) and the childhood adiposity common factor (see Methods).

**Fig. 3:**
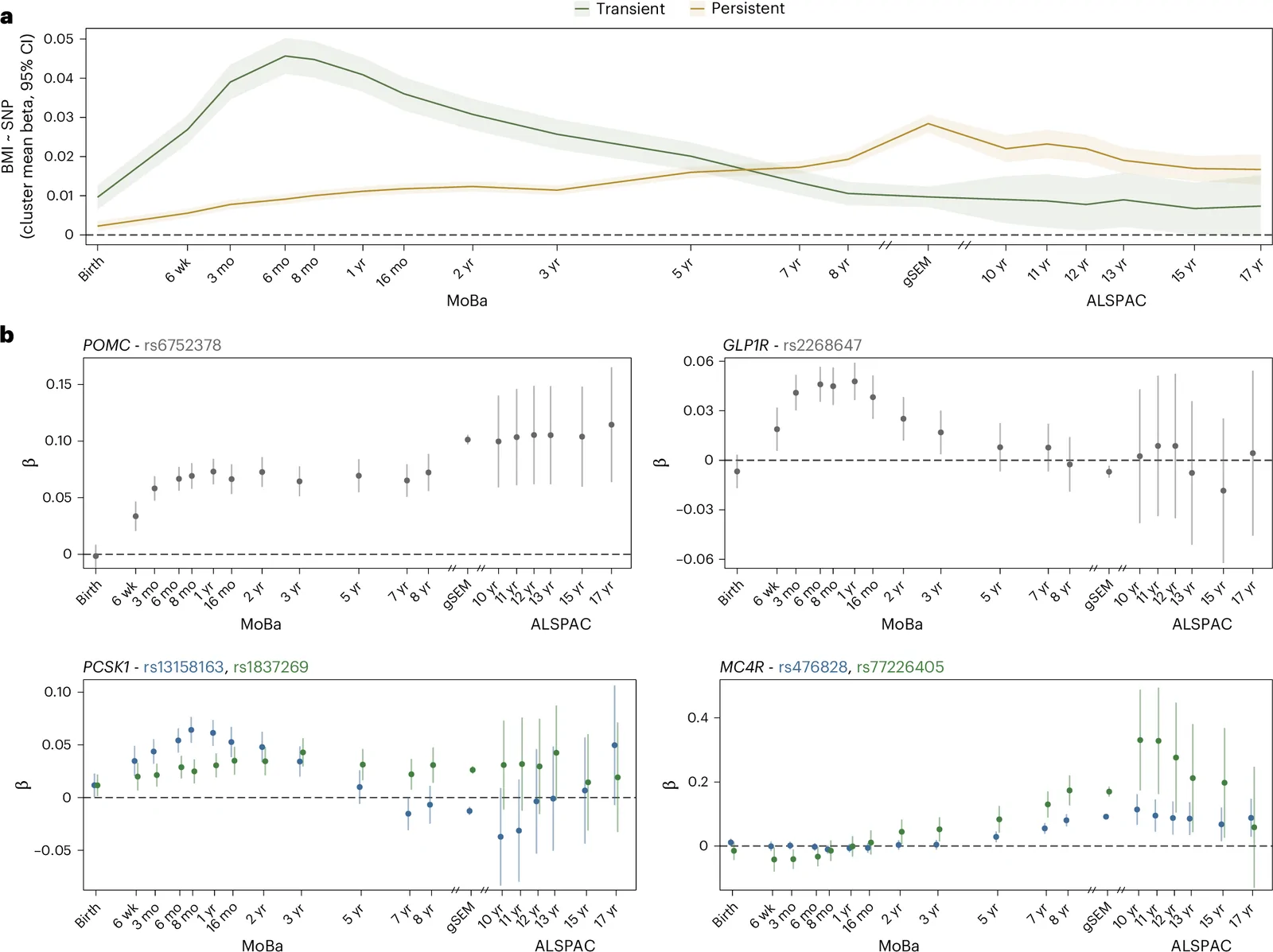
Clustering of variants by their effects on BMI across childhood. **a**, Mean effect estimates and 95% CIs for variants in each trajectory cluster at each timepoint in MoBa. **b**, Effect estimates (*z*-score of BMI) and 95% CIs for the SNP effect on childhood BMI in MoBa, the childhood adiposity common factor and on BMI in ALSPAC for selected individual variants: *POMC*, rs6752378-Persistent; *PCSK1*, rs13158163-Transient and rs1837269-Transient; *GLP1R*, rs2268647-Transient; *MC4R*, rs476828-Persistent and rs77226405-Persistent. Further data are in Supplementary Data 10 and 12, and information on sample size at each timepoint can be found in Supplementary Data 1.

**Extended Data Fig. 4:**
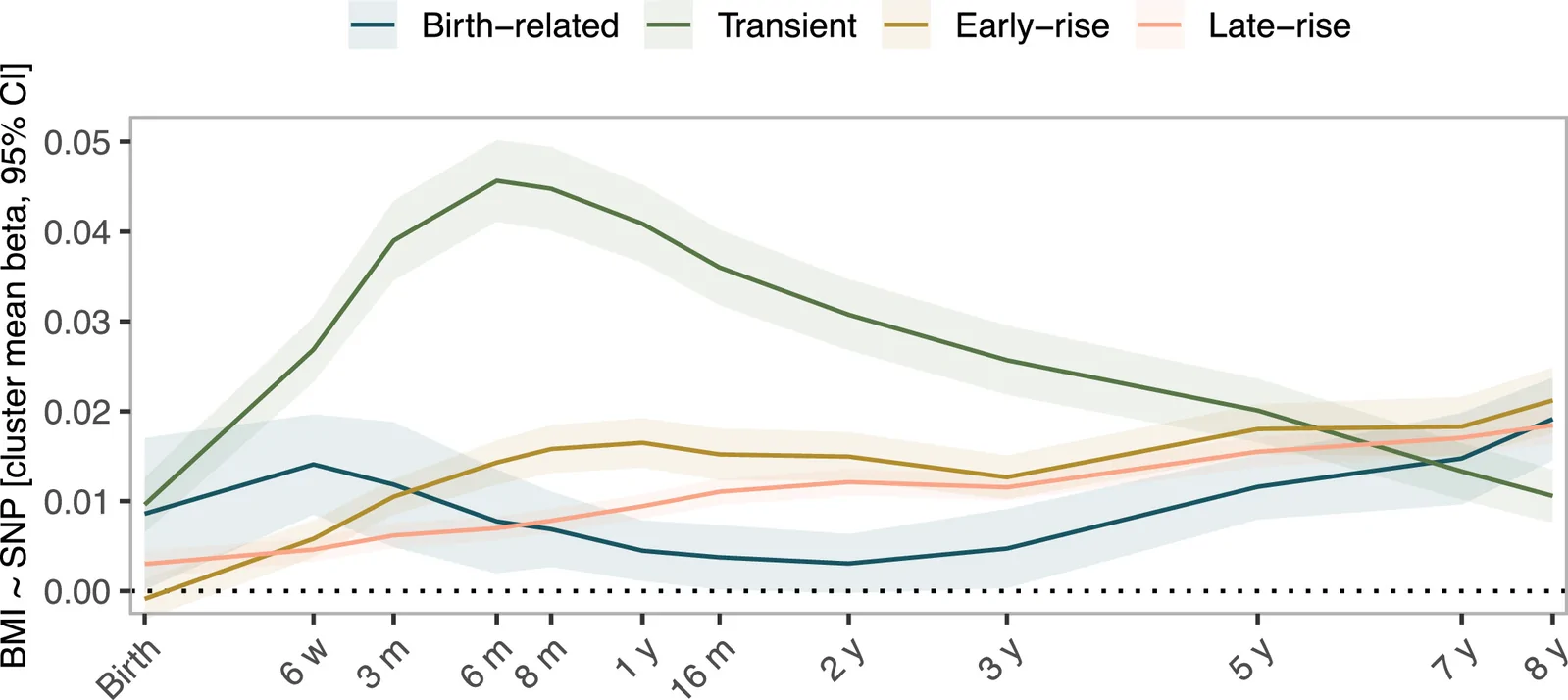
Four-cluster model. Mean effect estimates and 95 % CIs for variants according to the four-cluster model at each MoBa time-point.

A model with two clusters, ‘Persistent’ and ‘Transient’, showed the best fit (Fig. 3a and Supplementary Data 10 and 11). Signals in the Transient cluster (*n* = 115) showed associations with BMI during infancy only, peaking between 6 weeks and ~12 months. The Persistent trajectory signals (*n* = 507) were characterized by associations with BMI that continued into later childhood. Selected individual trajectories are displayed in Fig. 3b. Although the two-cluster model provided the best fit, a plausible subcategorization indicated that the Persistent cluster might be further divided based on age of onset: birth-related (32 signals), early-rise (145 signals) and late-rise (330 signals) (Supplementary Data 11 and Extended Data Fig. 4), as done previously^16^.

**Extended Data Fig. 5:**
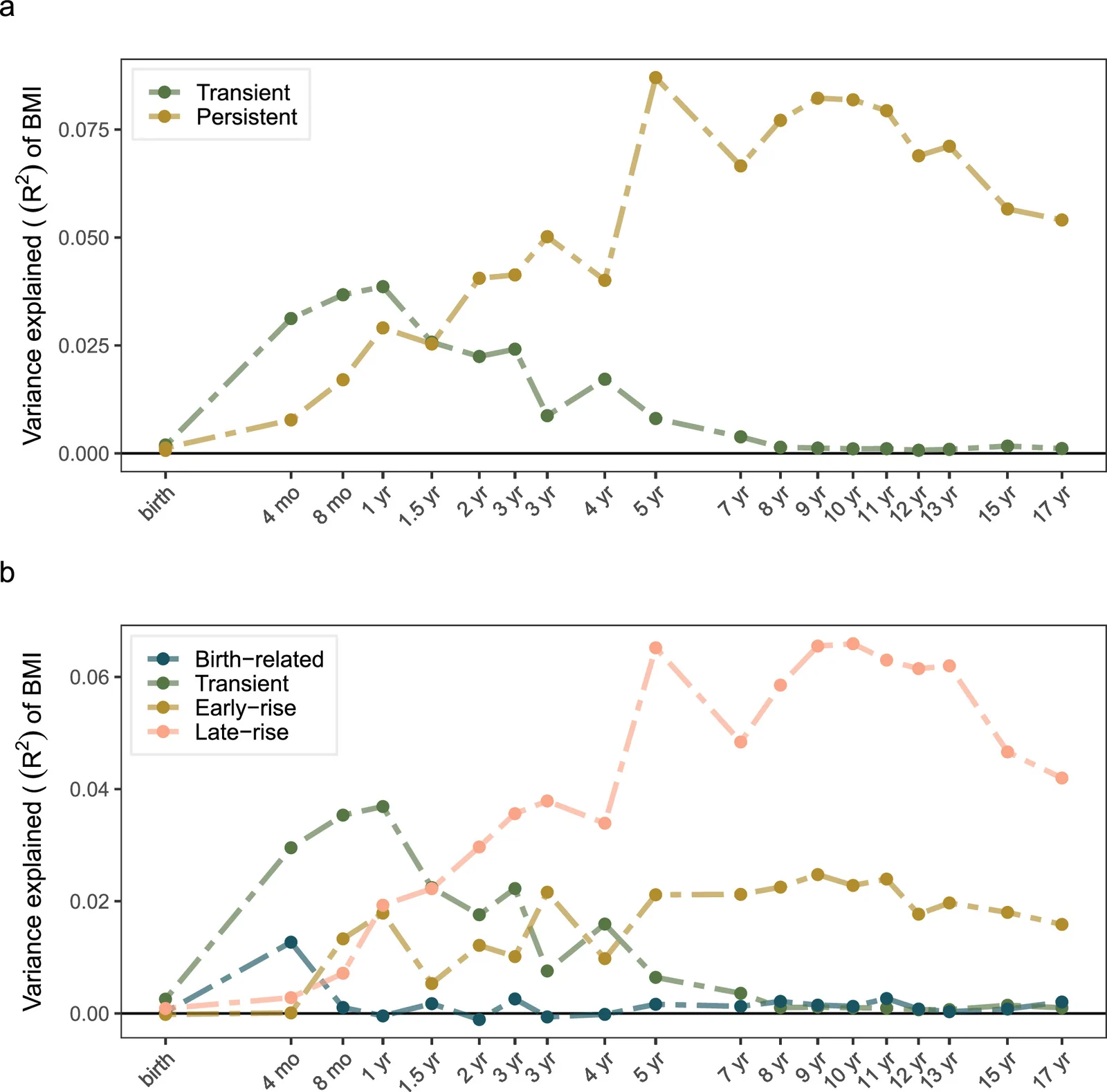
Trajectory-specific PGS performance in predicting childhood BMI in ALSPAC. Underlying data can be found in Supplementary Data 12. **a**, Performance of trajectory-specific PGSs derived from a two-cluster model. **b**, Performance of trajectory-specific PGSs derived from a four-cluster model.

We constructed trajectory-specific PGSs and observed confirmatory patterns of association with BMI across childhood in the ALSPAC cohort (Extended Data Fig. 5 and Supplementary Data 12) as well as for individual SNP trajectories (Fig. 3b).

### Variant-to-gene mapping implicates childhood adiposity genes

The GWAS to Genes (G2G) analysis framework combines data on expression and protein quantitative trait loci (eQTL and pQTL), enhancer maps and common coding variants (see Methods)^21^. This linked 434 ‘high-confidence’ genes to the 624 childhood adiposity signals (Supplementary Data 13). Among these were 11 components of the leptin–melanocortin pathway, which has an established role in appetite regulation and severe early-onset obesity^8^. The signals linked to *BDNF*, *GNAS*, *LEP*, *MC4R*, *NTRK2*, *NPY*, *PCSK2* and *POMC* mapped to the Persistent trajectory cluster, with *MC4R* and *PCSK2* harboring multiple signals, while signals linked to *BSX*, *LEPR* and *PCSK1* showed Transient BMI associations (Supplementary Data 14).

We also implicated incretin and gut peptide pathway components *GLP1R* (3× Transient and 1× unassigned signal), *GIPR* (2× Persistent) and *GRP* (1× Persistent), which are key regulators of postprandial metabolism and treatment targets for type 2 diabetes and obesity^23^. Other implicated genes are previously reported obesity susceptibility genes with roles in neuronal development and synaptic function^8^: *CADM1* (Persistent)*, CADM2* (Persistent) and *NEGR1* (Persistent) (Supplementary Data 14).

Of the 434 high-confidence childhood adiposity genes, 217 genes showed no evidence of association with adult BMI (for example, *GLP1R*, *BSX*, *GNAS*, *BBS9*) (Supplementary Data 15). A further 27 genes (for example, *LEPR*, *PCSK2*, *NEGR*, *DLK1* and *CALCR*) showed a more complex pattern, with both child-specific and shared childhood adiposity and adult BMI signals, indicating that some core obesity genes are differentially regulated between early life and later life adiposity. The remaining 190 genes have signals associated with both childhood adiposity and adult BMI.

### Putative imprinting effects resolved by trio-analysis

**Fig. 4:**
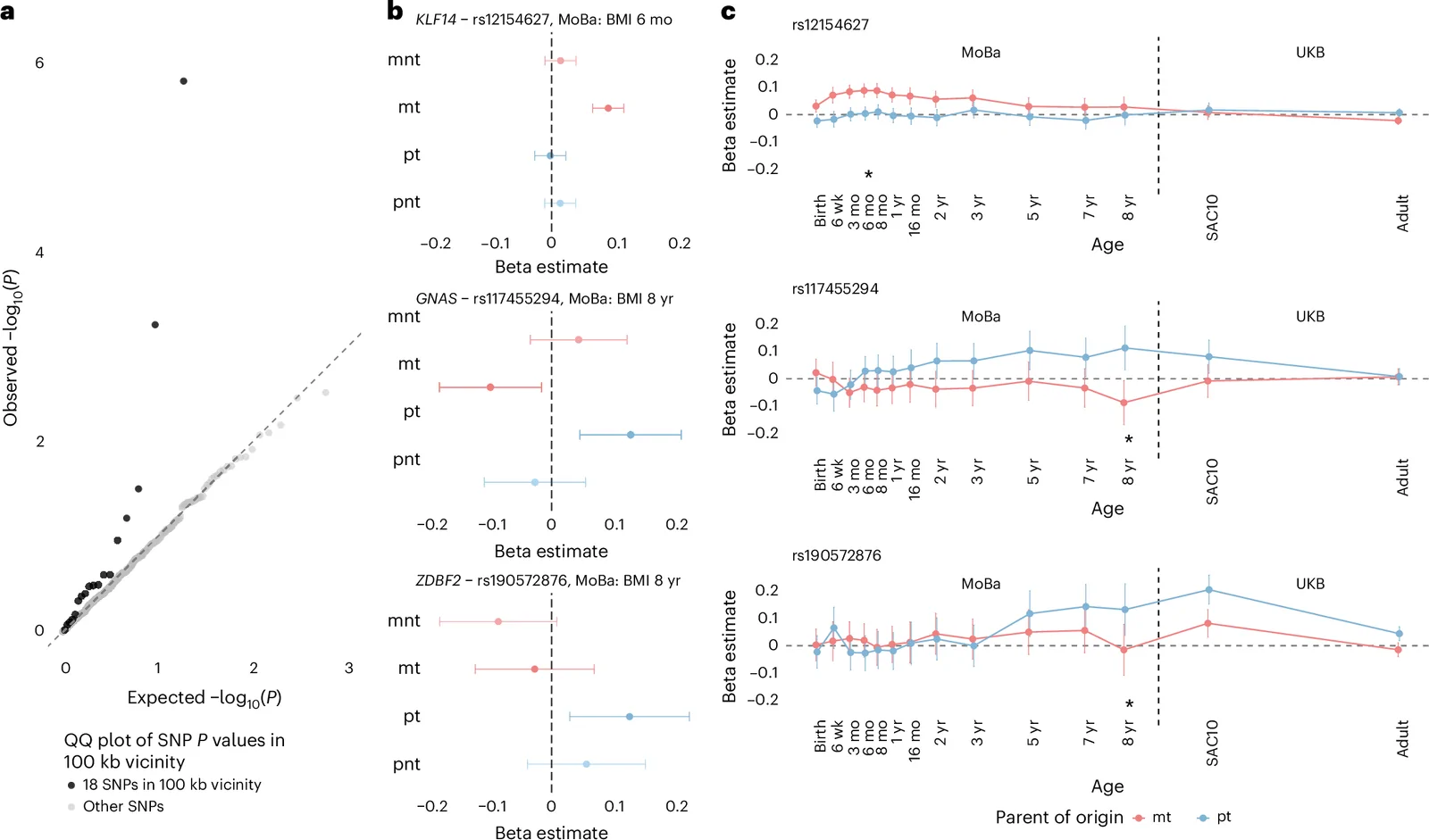
PoE for lead SNPs near known imprinted regions. **a**, Quantile–quantile (QQ) plot showing the distribution of observed versus expected *P* values for PoE analyses across all 624 GWAS signals, with 18 signals near known imprinted regions in dark and the remaining 606 signals in light gray. **b**, Forest plots showing effect estimates on BMI (*z*-score) and 95% CIs for maternally transmitted (mt), paternally transmitted (pt), maternal non-transmitted (mnt) and paternal non-transmitted (pnt) allele effects on BMI at the timepoint with the most significant PoE effect in MoBa for rs12154627 (*KLF14*, 6 months, *n* = 25,779), rs117455294 (*GNAS*, 8 years, *n* = 11,457) and rs190572876 (*ZDBF2*/*GPR1*, 8 years, *n* = 11,457). **c**, Trajectories of mt and pt associations by age from birth to adulthood in MoBa (*n* up to 25,779) and UK Biobank (UKB) (*n* up to 109,385). The asterisk highlights the timepoint with the most significant PoE association; error bars, 95% CIs. Further data are in Supplementary Data 16 and 17.

Imprinting is an epigenetic gene-silencing process resulting in parent-of-origin (PoE)-specific expression, which can be both tissue-specific and development-stage specific^24^. We compared the relative association of paternally inherited versus maternally inherited alleles with childhood BMI in up to 25,779 unrelated child–mother–father trios in MoBa to identify PoE effects (see Methods and Supplementary Data 16). Quantile–quantile plots showed greater-than-expected PoE associations among the 18 signals near known imprinted regions, and no inflation among the remaining 606 signals outside imprinted regions (Fig. 4a,b).

Evidence for PoE was found at rs12154627-*KLF14* (a maternal Transient signal) on infancy BMI, confirming our previous observations^16,25^. A previously unreported PoE was observed for rs117455294 (Persistent), located in a differentially methylated region at the *GNAS* locus, showing bipolar dominance; that is, directionally opposing effects between maternally transmitted and paternally transmitted alleles. A third signal, rs190572876 (Persistent), showed a novel paternally transmitted allele association with BMI at 8 years and was also among the strongest gSEM signals for childhood adiposity (*P* = 9.5 × 10^−49^), located near a conserved imprinted region proximal to *ZDBF2* (refs. ^26,27^). Paternally expressed *Zdbf2* is reported to regulate glucose homeostasis and neuropeptide signaling and growth^28^. Although G2G prioritized *NDUFS1* at this locus (Supplementary Data 13), the paternally transmitted effects of this signal on childhood BMI and SAC10 make *ZDBF2* a more plausible causal gene.

We extended these findings by applying a novel method to infer PoE in large cohorts without parental genotyping^29^ in up to 109,385 UK Biobank participants. This provided life trajectories of these PoEs, including opposing effects between infancy and adult BMI at the *KLF14* locus, the childhood-specific paternal effect near *GNAS* and the predominantly paternal effect near *ZDBF2* on both SAC10 and adult BMI (Fig. 4c and Supplementary Data 17).

### Biological pathways enriched for childhood adiposity genes

**Extended Data Fig. 6:**
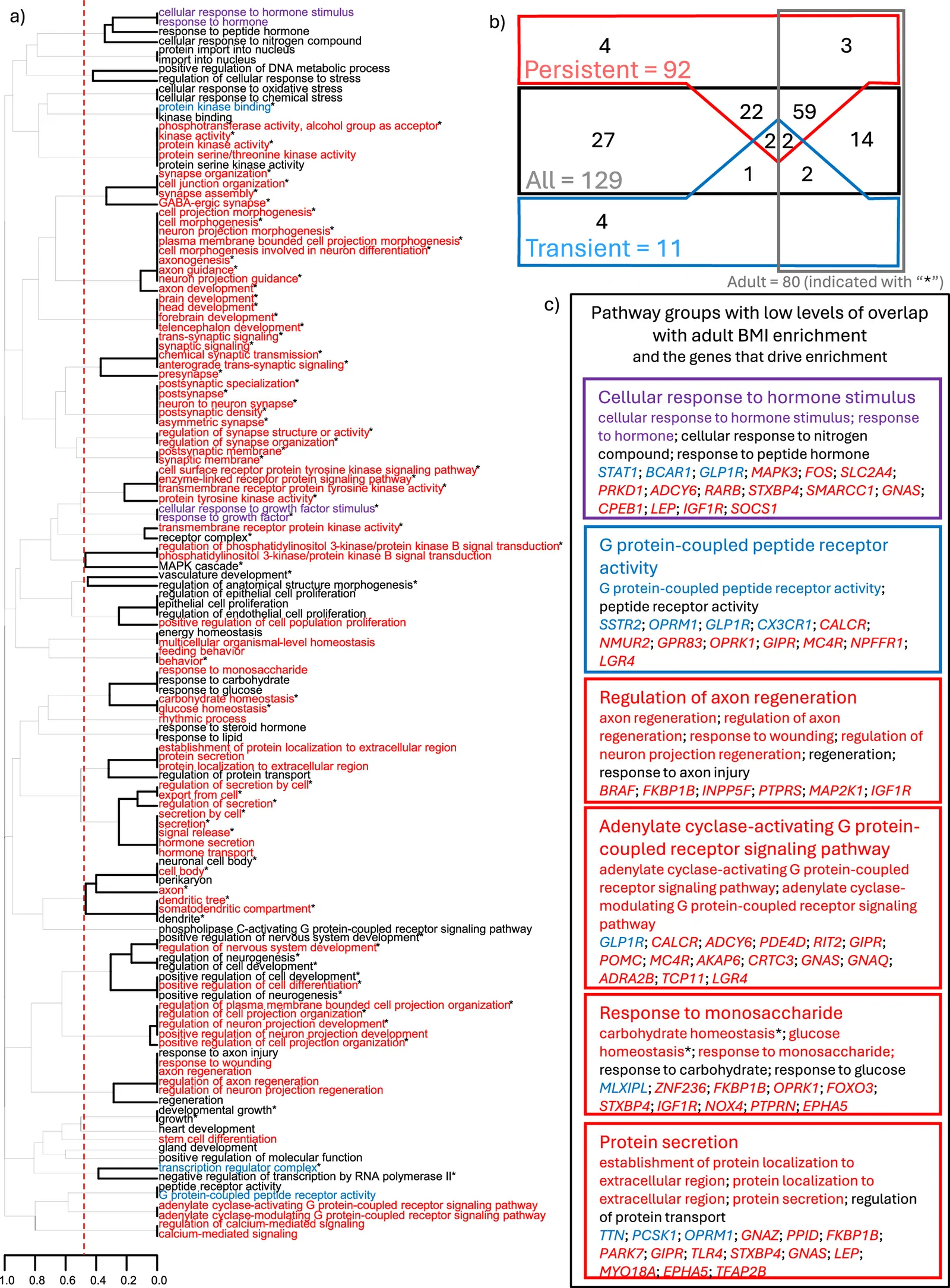
Enriched biological pathways. **a**, Dendrogram of the 129 pathways enriched for the complete list of childhood adiposity association signals, analysed in gProfiler. Clustering is scaled by our custom distance metric (see Methods). Pathway names are colored if also enriched for signals in each of the two childhood adiposity trajectories. * indicates the pathways also enriched for adult BMI signals. **b**, Numbers of enriched pathways by trajectory and the overlap. **c**, Clustered pathway groups with little or no enrichment for adult BMI. Individual pathways and genes driving enrichment are displayed in smaller font size. More details can be found in Supplementary Datas 18 and 19.

Enrichment analyses using the 434 high-confidence childhood adiposity genes identified 11 biological pathways enriched for Transient trajectory genes and 92 pathways for Persistent trajectory genes, including four pathways enriched for both trajectories (Supplementary Data 18 and Extended Data Fig. 6). A complete clustering method^21^ yielded 40 enriched pathway groups (Supplementary Data 19). Both Transient and Persistent trajectory genes were enriched in the ‘Cellular response to hormone stimulus’ pathway group. Persistent signals were enriched in the ‘feeding behavior’, ‘dendrites’, ‘synaptic signaling’, ‘neuron projection’, ‘G protein-coupled peptide receptor activity’, ‘protein kinase activity’, ‘secretion’ and ‘transmembrane receptor protein signaling’ pathway groups.

When compared to the same pathway enrichment approach using adult BMI GWAS data, we found that 80 of the childhood adiposity enriched pathways were also enriched for adult BMI associations (including four out of seven Transient pathways and 64 out of 92 Persistent pathways). Conversely, 60 individual pathways and six pathway groups appeared specifically enriched for childhood adiposity signals, including ‘cellular response to hormone stimulus’, ‘regulation of axon regeneration’ and ‘response to monosaccharide’ (Extended Data Fig. 6).

### Links to adult diseases

**Extended Data Fig. 7:**
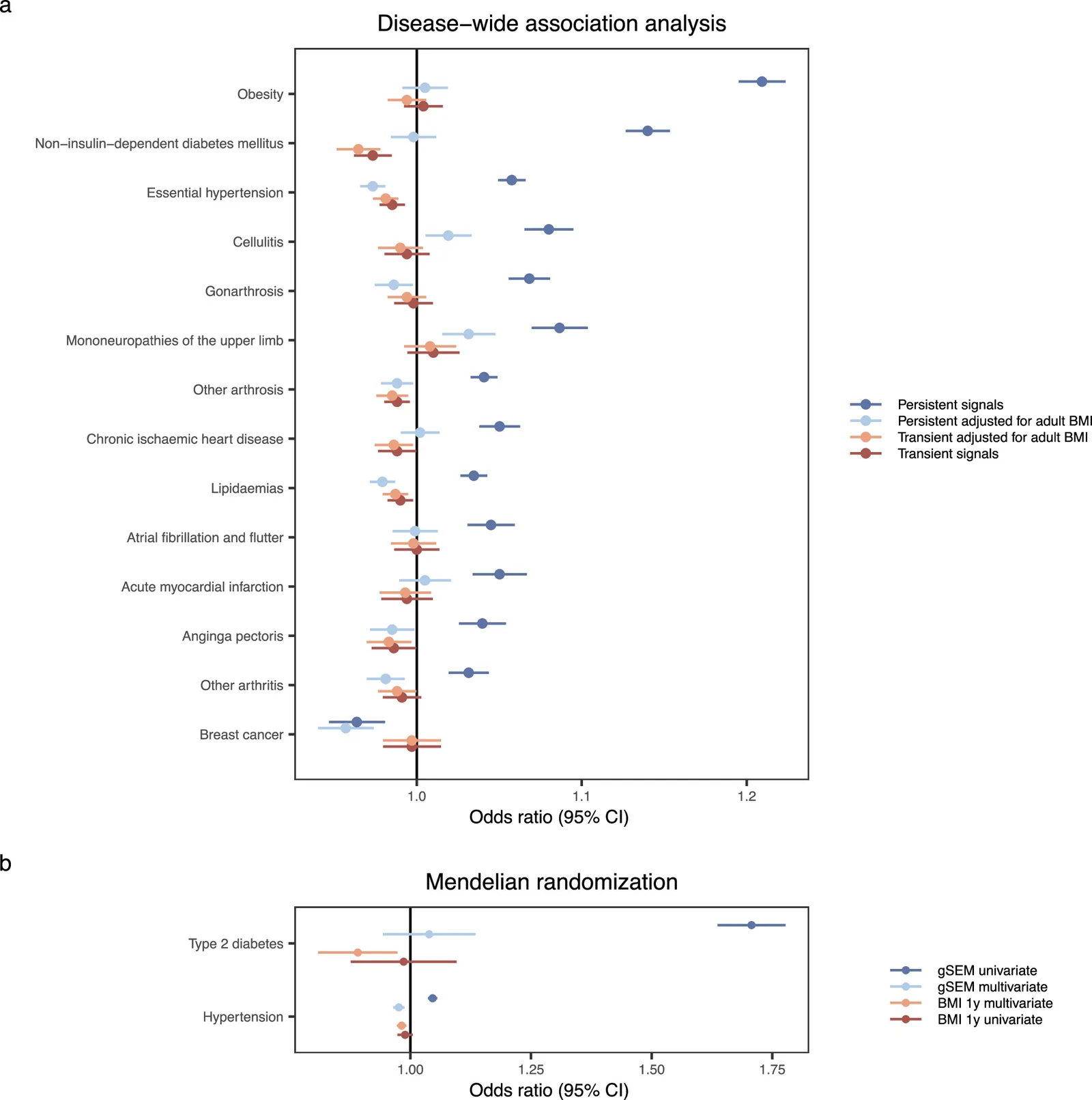
Disease-wide association analysis and Mendelian randomization analysis. **a**, Odds ratios and 95% CIs of the trajectory-specific genetic scores on selected traits from the disease-wide association analysis. **b**, Discovery-analysis specific inverse-variance weighted odds ratios and 95% CIs from Mendelian randomization of type 2 diabetes and hypertension. Further data can be found in Supplementary Datas 20 and 21.

We performed a disease-wide analysis with at least 20,000 cases in UK Biobank using PGS for the Transient and Persistent adiposity trajectories (Supplementary Data 20 and Extended Data Fig. 7). Persistent adiposity signals showed association with higher risks of several cardiometabolic outcomes that disappeared when controlling for adult BMI. This finding is consistent with prior Mendelian randomization (MR) studies indicating that childhood adiposity associations with later cardiometabolic health are predominantly mediated through association with adult BMI^14^. As previously reported, breast cancer showed a childhood adiposity-specific protective association^14^. Transient adiposity signals did not confer higher risk for any disease. Conversely, they appeared protective against non-insulin-dependent diabetes (*P* = 1.4 × 10^−5^) and essential hypertension (*P* = 1.0 × 10^−4^), and these protective effects strengthened when controlling for adult BMI.

We investigated early adiposity links with type 2 diabetes and hypertension using both univariable and multivariable MR, modeling as the exposures childhood adiposity using gSEM signals and early life adiposity using BMI at 12 months signals (Supplementary Data 21 and Extended Data Fig. 7). Childhood adiposity showed positive associations with type 2 diabetes and hypertension in univariable MR, and these were fully attenuated when controlling for adult BMI in multivariable MR. By contrast, early adiposity showed a protective association with these traits in multivariable MR accounting for adult BMI.

Together, these findings suggest that childhood adiposity increases adult cardiometabolic disease risks largely through higher adult BMI, whereas a transient rise in infancy adiposity that subsequently resolves may confer protective effects.

### Comparison of effects in childhood and adulthood

Our 624 childhood adiposity GWAS signals included 165 signals previously reported for adult BMI at *P* < 5 × 10^−8^, and a further 264 signals with at least nominally significant (*P* < 0.05) directionally concordant associations with adult BMI^6^ (Supplementary Data 22). More signals in the Persistent trajectory (77%) showed at least nominally significant (*P* < 0.05) directionally concordant associations with adult BMI than did signals in the Transient trajectory (43%).

**Fig. 5:**
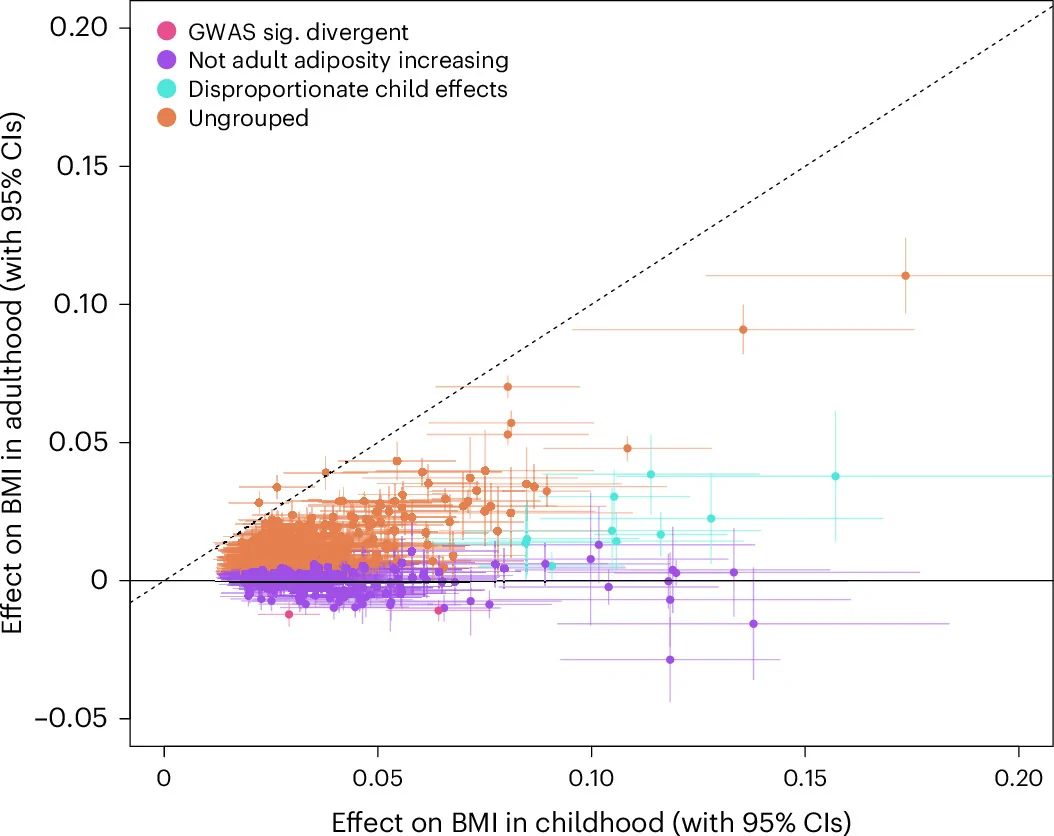
Comparison of childhood and adult BMI effects of identified GWAS signals. Signals identified, stratified by their effects on standardized BMI in childhood and in adulthood. Error bars, 95% CIs. Further data are in Supplementary Data 22.

Conversely, 194 out of our 624 childhood GWAS signals showed either no association (*P* > 0.05, *n* = 165) or a nominally significant but directionally opposite association (*n* = 29) with adult BMI (Fig. 5 and Supplementary Data 22). These signals highlighted genes in the leptin–melanocortin pathway (*BSX*, *GNAS* and *LEPR*), incretin signaling (*GIPR* and *GLP1R*) and the imprinted locus near *KLF14*. Among a further ten signals with disproportionately larger effects on childhood than adult BMI were two variants at imprinted loci (*ZDBF2* and *DLK1*).

Notably, two signals showed directionally opposite, genome-wide significant associations with BMI in children and adults: one of them at *PCSK1* (rs13158163-Transient) showing opposing associations with BMI in infancy and adulthood (β_8m_ = 0.064, *P*_8m_ = 7.3 × 10^−25^; β_adult_ = −0.011, *P*_adult_ = 1.3 × 10^−8^). The roles of common^30^ and rare variants^31^ at *PCSK1* in obesity have been previously reported; however, the signal tagged by rs13158163 is restricted to the infancy period and distinct from the other *PCSK1* signal that has a Persistent association with BMI (rs1837269) (Fig. 3b). Age-dependent patterns have also been reported in patients with rare biallelic inactivating mutations in *PCSK1* (ref. ^31^).

### Hypothalamic neuron populations enriched for childhood adiposity genes

**Fig. 6:**
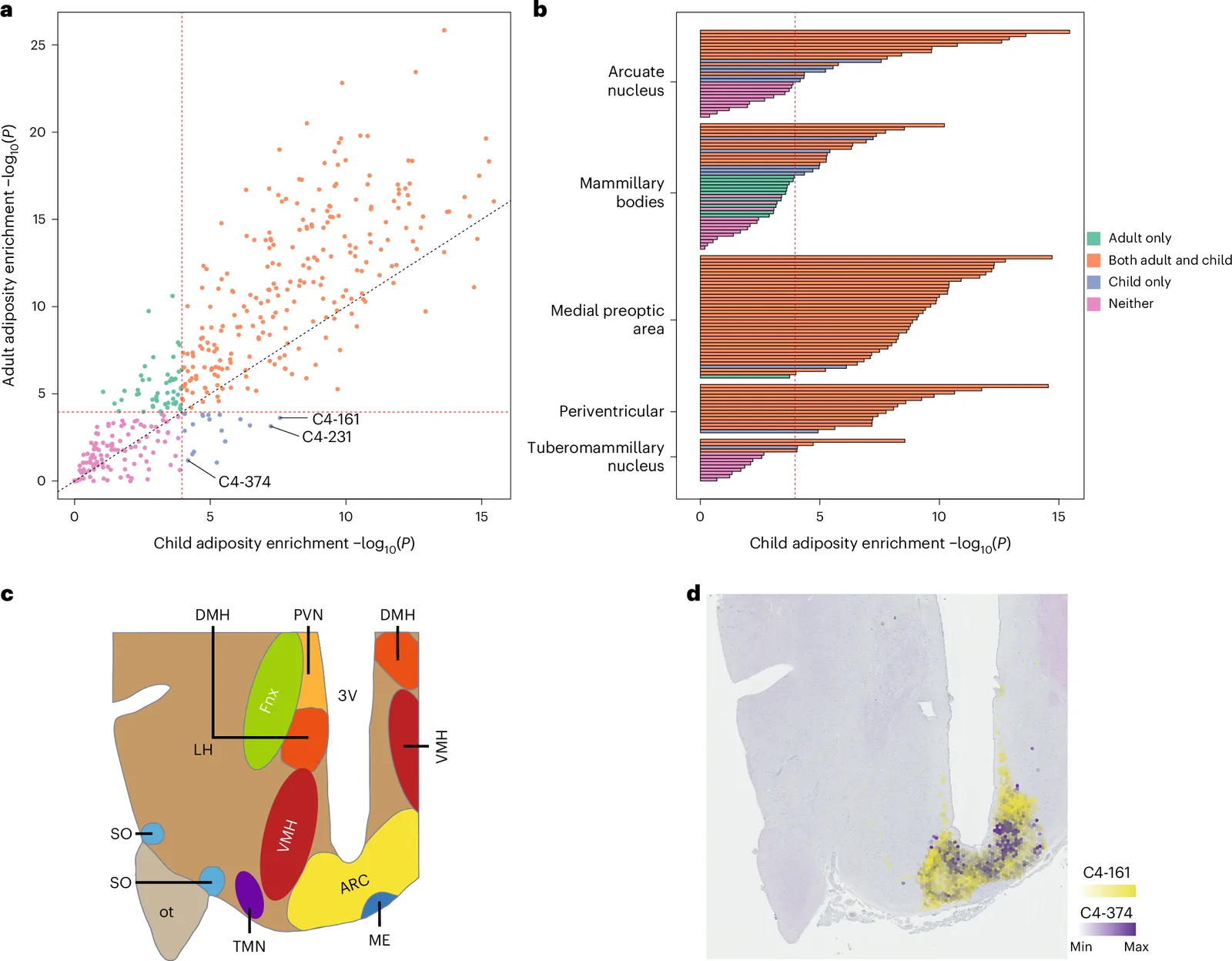
Hypothalamic cell populations specifically enriched for childhood adiposity genes. **a**, Points represent the 452 HYPOMAP-reported hypothalamic cell populations plotted by the *P* values for enrichment for adult BMI (*y* axis) and childhood adiposity associations (*x* axis). Blue points indicate the 19 cell populations enriched only for childhood adiposity. **b**, Brain regions containing the childhood-specific cell populations are shown in blue columns; other cell populations in these regions are indicated by other colors. Dashed red lines indicate the Bonferroni-corrected threshold (*P* < 0.05 / 452). **c**, Regional annotation of the human hypothalamus (DMH, dorsal medial hypothalamus; VMH, ventral medial hypothalamus; PVN, paraventricular nucleus; TMN, tuberomammillary nucleus; LH, lateral hypothalamus; SO, supraoptic nucleus; Fnx, fornix; ME, median eminence; 3V, 3^rd^ ventricle; ot, optic tract). **d**, Estimated spatial localization of C4-161 (Mid-1 GABA-6 IL13RA1 GHRH) and C4-374 neurons (Mid-2 GABA-GLU-3 POMC CALCR) within the human hypothalamic ARC. Relevant data are in Supplementary Data 23 and 24.

**Extended Data Fig. 8:**
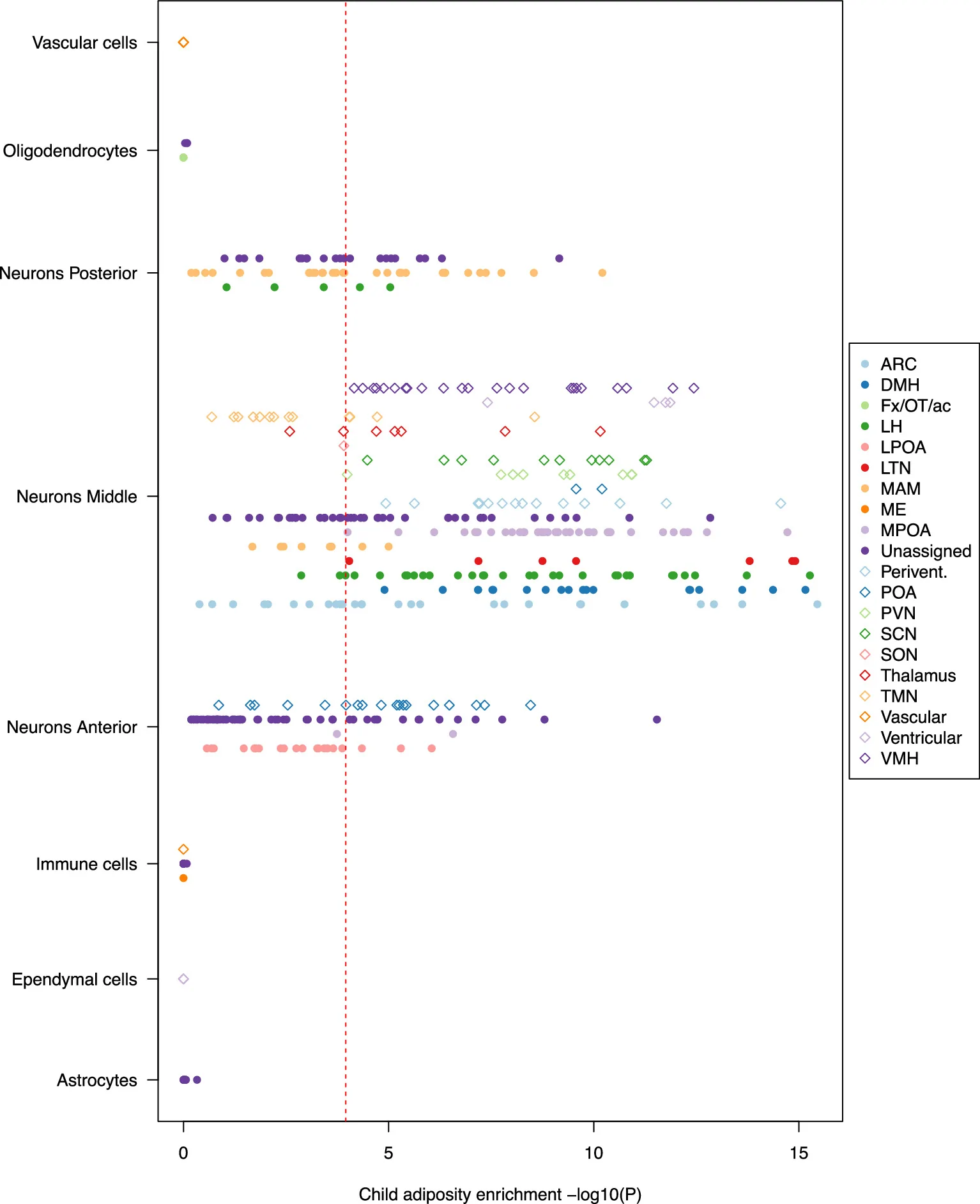
Neuronal cell populations are enriched for genes linked to childhood adiposity variation in the general population. Prioritization of 452 human hypothalamic cell types identified 251 cell types as significantly enriched for associations in the filtered gSEM GWAS. Each point represents a cell type and cell types were grouped (*y*-axis) by neuronal or non-neuronal cell types and coloured based on specific regions assigned via marker-gene expression. The enrichment was assessed using a one-tailed *P*-value calculated using MAGMA, with the dashed line indicating a Bonferroni-corrected significance threshold, *P* < 0.05/452. Further data can be found in Supplementary Data 23.

In the human hypothalamic single-nucleus RNA sequencing (snRNA-seq) data (HYPOMAP)^32^, our filtered childhood adiposity GWAS signals were enriched in 260 of the 452 reported hypothalamic cell populations (at *P* < 0.05 / 452). All of these 260 were neuronal populations, and 19 of them were not also enriched for adult BMI GWAS signals (Fig. 6, Supplementary Data 23 and Extended Data Fig. 8). Five of these 19 ‘child-specific cell populations’ mapped to the arcuate nucleus (ARC), which has key roles in energy homeostasis, somatic growth and puberty onset^33^, and a further five mapped to the mammillary bodies (MAM).

Of the high-confidence childhood adiposity genes, nine contributed strongly to cell-type classification of the five child-specific ARC cell populations (see Methods). These included components of the leptin–melanocortin (*LEPR* and *POMC*) and incretin and gut peptide pathways (*GLP1R* and *GRP*), and the imprinted gene *DLK1* (Supplementary Data 24). Similarly, 11 childhood adiposity genes were informative marker genes for the five childhood-specific MAM cell populations. These also included components of the leptin–melanocortin (*BDNF*, *MC4R* and *PCSK1*) and incretin and gut peptide pathways (*GIPR* and *GRP*).

The highest ARC cell population enriched for childhood adiposity GWAS signals was C4-161 (Mid-1 GABA-6 IL13RA1 GHRH), which is marked by expression of the childhood adiposity genes *LEPR*, *GLP1R* and *DLK1*. The highest MAM cell population enriched for childhood adiposity GWAS signals was C4-231 (Post-1 GLU-1 EBF2 PLEKHH2), which is marked by expression of *BDNF* and *GIPR* (Supplementary Data 24).

### WGS identifies rare variants affecting childhood adiposity

We performed rare variant burden tests using WGS data in up to 479,615 all-ancestry UK Biobank participants within two non-overlapping predicted functional categories: high-confidence protein truncating variants (PTVs) and predicted deleterious missense variants.

**Fig. 7:**
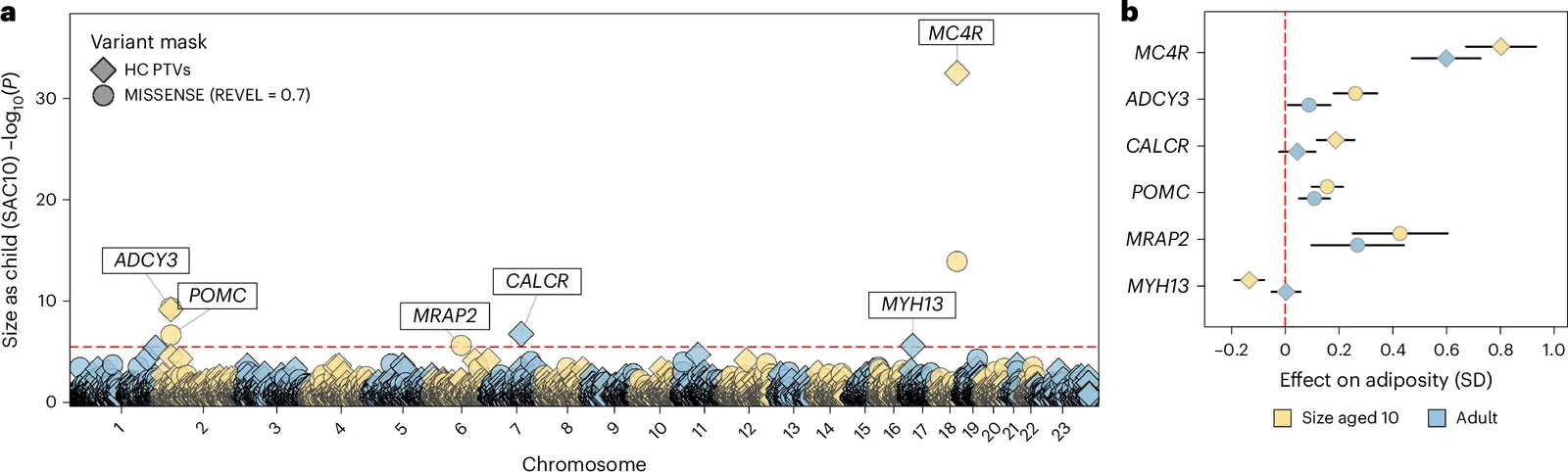
Rare variant gene burden associations with childhood adiposity (SAC10). **a**, Manhattan plot showing genome-wide gene associations with two-sided *P* values from a burden test performed using BOLT-LMM (*n* = 479,615). Genes passing the multiple-testing-corrected threshold (*P* < 2.9 × 10^−6^) are labeled. Predicted variant function masks are indicated by point shapes. **b**, Identified gene burden associations with childhood adiposity (SAC10) and their associations with adult BMI; error bars represent 95% CIs. Relevant data are in Supplementary Data 25 and 26.

**Extended Data Fig. 9:**
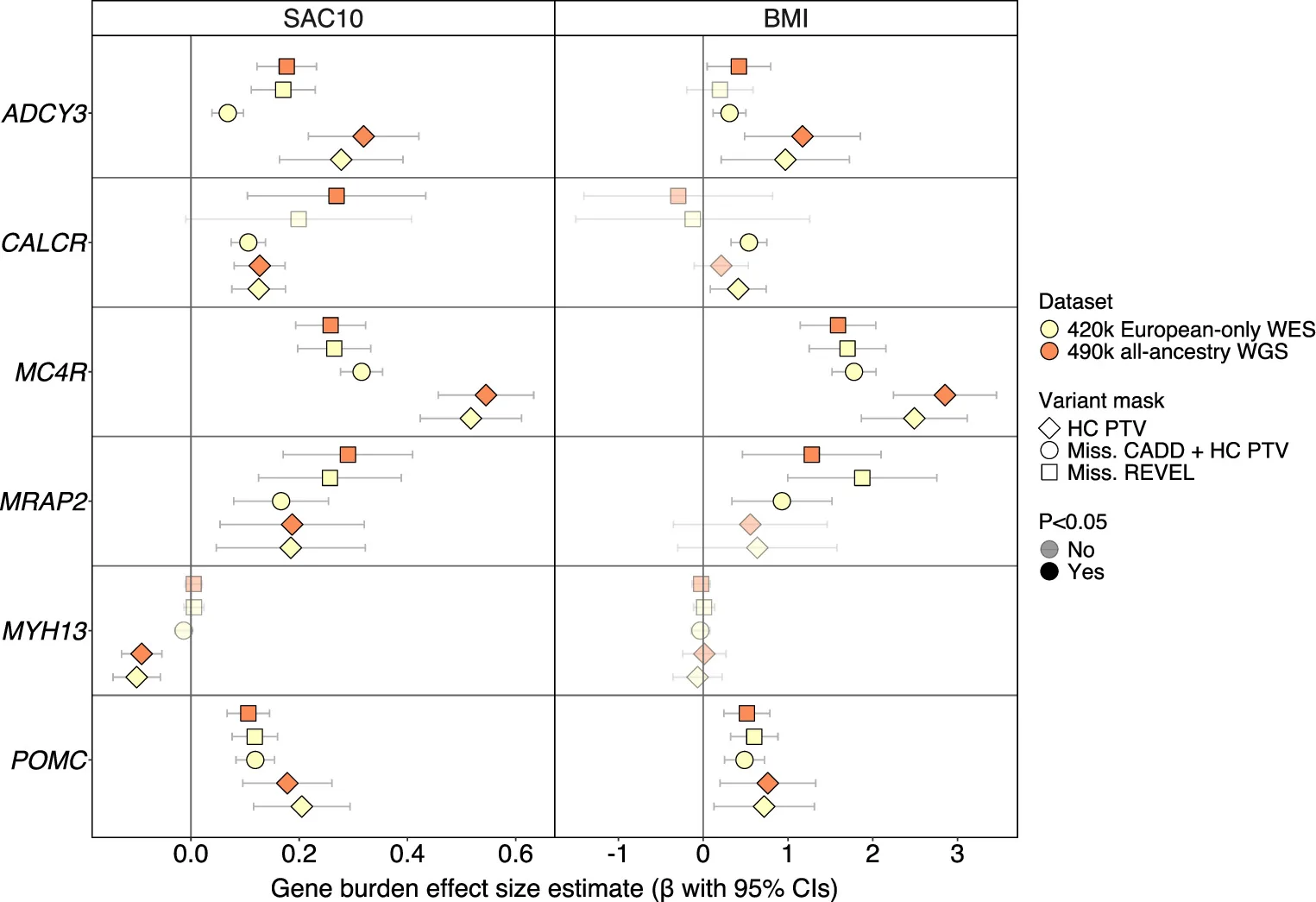
Sensitivity analysis for WGS identified genes for childhood adiposity in UK Biobank WES data. Gene burden test effect estimates from BOLT-LMM on SAC10 (three category model) and adult BMI (kg/m^2^) for the six genes identified in the UK Biobank WGS analysis of rare deleterious variants. For comparison, similar effect estimates are shown in the smaller UK Biobank WES dataset. Point opacity indicates at least nominal association (*P* < 0.05, two-tailed). Point shapes indicate the variant collapsing mask, and colors indicate the source dataset. Error bars indicate 95% confidence intervals. Further data can be found in Supplementary Datas 25 and 26.

Six genes were associated with SAC10 at a Bonferroni-corrected threshold (*P* < 2.9 × 10^−6^ (0.05 / 17,265 tests); Fig. 7, Supplementary Data 25 and Extended Data Fig. 9). These included *POMC*, *CALCR* and *MC4R*, confirming our previous report in a smaller UK Biobank sample with whole-exome sequencing^34^. Additionally, in *ADCY3*, both rare heterozygous deleterious missense variants (β = 0.177, *P* = 4.4 × 10^−10^, *n* = 575) and PTVs (β = 0.319, *P* = 6.4 × 10^−10^, *n* = 173) conferred larger SAC10. Homozygous loss-of-function of *ADCY3* was previously linked to severe obesity in adults^35,36^. Furthermore, rare heterozygous deleterious missense variants in *MRAP2* conferred larger SAC10 (β = 0.29, *P* = 2.2 × 10^−6^, *n* = 123), consistent with previous findings in hyperphagic obese children and adults^37^. Finally, we observed a novel association with SAC10 at *MYH13* (β = −0.1, *P* = 2.5 × 10^−6^, *n* = 1,237 PTV carriers), and PTVs in *MYH13* showed no association with adult BMI (*P* > 0.05). Although the units of effects are not directly comparable, stronger rare variant associations with SAC10 than adult BMI were apparent for all six genes. This observation persisted when we extended association testing at these genes to additional variant masks and other obesity-related traits in UK Biobank (Supplementary Data 26).

## Discussion

Combining time-resolved childhood BMI from MoBa with UK Biobank data on recalled childhood adiposity and puberty timing, we generated an effective sample size of 599,924 individuals for childhood adiposity. This enabled the discovery of 624 independent signals for childhood adiposity, far more than previously reported and comparable to findings for adult BMI (941 signals in >700,000 individuals). Notably, nearly one-third (196 out of 624, 31%) of these signals had no concordant effect in adulthood. The apparent differential regulation of child and adult adiposity is also supported by our demonstration of childhood-specific adiposity-regulating cell populations in the hypothalamic ARC and MAM, and the latter finding suggests a possible link between recollective memory or emotional regulation and energy homeostasis, specifically in early life. In both tissues, these childhood-specific cell populations were marked by expression of components of the leptin–melanocortin and incretin pathways. We reason that several child-specific adiposity signals may act through cell-specific regulators of gene expression, rather than through child-specific adiposity genes and pathways.

Our high-confidence childhood adiposity genes included 11 leptin–melanocortin pathway genes, as well as incretin pathway genes and other obesity susceptibility genes supported by reported evidence from animal models^8^. Two leptin–melanocortin pathway genes were highlighted only by childhood-specific signals (*BSX* and *GNAS*). Others contained multiple independent signals with differing effects on adult BMI, such as *LEPR*, in which only one of four signals had a concordant effect on adult BMI. Similarly for incretin receptor genes, only one of two signals linked to *GIPR* appeared to alter adult BMI, and none of the *GLP1R* signals was associated with BMI past early childhood. These findings of genes with multiple life-course-dependent adiposity signals support the idea of life-course-dependent regulation, whereby signals may have differential actions on cell-specific regulators of gene expression rather than on age-specific genes and pathways. Similarly, in our WGS analysis, it is notable that only one of our six rare variant-associated genes (*MC4R*) was also identified in a similar analysis of adult BMI in the UK Biobank, which identified eight other genes^38^, including novel determinants of adult-onset obesity. Four of the six genes we highlighted encode central components of the leptin–melanocortin pathway that have been implicated in severe obesity^8^. We provide robust population-based evidence for rare heterozygous variant effects (not previously reported for *ADCY3* and *MRAP2*) and note stronger associations for all genes with SAC10 than with adult BMI. These findings suggest that drugs that target this pathway (such as leptin and setmelanotide) may have stronger effects on adiposity in children than in adults.

Our findings also highlight the value of integrating evidence from multiple child obesity-related traits to produce robust findings with high concordance on independent validation. Although we excluded signals primarily driven by puberty, some remaining childhood adiposity signals were linked with high confidence to established regulators of sex hormone secretion: *GNRH1* (rs7832399, Persistent), encoding a key hypothalamic molecule in the hypothalamic-pituitary-gonadal axis, and *FGFR1* (rs881301, Persistent), in which mutations cause hypogonadotropic hypogonadism. These two signals showed weak associations with AAM and had concordant associations with childhood BMI at earlier pre-pubertal timepoints, confirming their action through childhood adiposity. A further transient adiposity signal was linked to the sex hormone receptor *ESR1* (rs1293936). These findings raise the prospect that the temporary physiological activation of sex hormone secretion during infancy (‘mini-puberty’) might have downstream effects on adiposity^39,40^.

We highlight two unique aspects of our study design. First, the multiple repeated BMI measurements in MoBa allowed us to discriminate with fine resolution between different childhood age effects. This differentiation is important, as BMI changes dramatically during early life, characterized by a peak of adiposity around age 6–9 months, thereafter declining BMI that reaches a minimum around age 5–6 years of age (the adiposity rebound). Our results show that these changes are mirrored by substantial changes in the genetic architecture between these life stages. Time-stratified PGS performance peaked in relatively narrow age windows, illustrating the value of choosing age-matched signals and weights in studies aiming to predict childhood adiposity or explore its causal relationships. A phenome-wide association study using Transient adiposity signals and a multivariate MR framework allowed isolating the effect of early life adiposity on later disease risk, suggesting that higher adiposity in infancy that later resolves has no adverse health consequences and may even have beneficial effects on risk for type 2 diabetes and hypertension.

Second, the availability of child–mother–father trios in MoBa provided unique insights into the role of genomic imprinting on childhood adiposity. It was originally proposed that imprinting evolved to allow the expression of conflicting parental-specific interests on offspring fetal growth (growth-restrictive maternal interests and growth-promoting paternal interests)^41^. This concept was recently extended to include parental conflict over puberty timing, which impacts the child’s dependence on maternal resources^42^. Our findings further extend the parental conflict hypothesis to include the regulation of childhood adiposity. The growth and metabolic effects of *GNAS* and *KLF14* are well described in humans, and our findings provide new insights into the complex life-long consequences of PoE at these loci. We demonstrate that the PoE at the *GNAS* locus seems confined to childhood, and the PoE trajectory at the maternally expressed *KLF14* locus shows opposing effects on BMI during infancy and adulthood. Although paternally expressed *Zdbf2* has been reported to regulate early feeding and growth with transient effects in mice^28^, our study is the first to suggest a paternal-only transient association at this region in humans. The imprinted *GPR1*–*GPR1AS*–*ZDBF2* region is well conserved between human and mouse genomes, and further studies are needed to clarify the role of *ZDBF2* in humans.

We acknowledge some limitations of our approach. Although the inclusion of data on puberty timing increased statistical power, larger studies with measured BMI during early childhood are needed to confirm their primary effects on adiposity and to detect sex-differential effects. The hard clustering approach assigns SNPs to a single cluster and yielded two clusters as the best fit. Although this process facilitated the identification of cluster-level associations, we note considerable variability in the age at onset and progression of effects for variants in the Persistent cluster. Larger studies and more refined clustering methods will enable more robust classifications of individual signals into early-rise and late-rise persisting clusters, as proposed previously. We included data on childhood adiposity only from northern European ancestry cohorts, and future studies with more population diversity are needed. We used BMI in infancy as a marker of adiposity. This method is supported by studies of fat mass measured by more accurate methods such as air-displacement plethysmography^43^ and total body electrical conductivity^44^. Large studies with measured infant body composition and genotypes are needed. We used the standard *P* < 5 × 10^−8^ threshold to indicate genome-wide statistical significance owing to the positive correlation in BMI across timepoints and to facilitate comparison with findings of other studies. Although this could lead to false-positive findings, there was high concordance with previous GWASs of measured childhood BMI^10^ (Supplementary Data 4 and 9). Finally, our data on HYPOMAP were derived from autopsy material from donors aged 60+ years^32^. Future samples from childhood donors or age-specific animal models would inform whether apparent childhood-specific effects are driven by changes in gene expression or other processes.

In conclusion, these findings emphasize the value of expanding research on childhood adiposity alongside studies in adults. Large-scale genetic studies of childhood adiposity provide richer evidence for energy homeostasis mechanisms than studies of adult BMI, suggesting that childhood may provide a more sensitive window for detecting variation in key endocrine and neuropeptide pathways regulating energy balance.

## Methods

### Ethics declarations

Informed consent was obtained from all MoBa study participants. The administrative board of the Norwegian MoBa study, led by the Norwegian Institute of Public Health (NIPH), approved the study protocol. The establishment of MoBa and initial data collection was based on a license from the Norwegian Data Protection Agency and approval from the Regional Committee for Medical Research Ethics. The MoBa cohort is currently regulated by the Norwegian Health Registry Act. The study was approved by the Regional Committee for Medical Research Ethics (2012/67).

Informed consent was obtained from all UK Biobank study participants. The study has approval from the North West Multicentre Research Ethics Committee as a Research Tissue Bank.

Ethical approval for the study was obtained from the ALSPAC Ethics and Law Committee and the Local Research Ethics Committees. Informed consent for the use of data collected through questionnaires and clinics was obtained from participants following the recommendations of the ALSPAC Ethics and Law Committee at the time. Consent for biological samples was collected in accordance with the Human Tissue Act (2004).

### MoBa GWAS

The Norwegian MoBa is a pregnancy-based ongoing cohort study consisting of approximately 114,500 children, 95,200 mothers and 75,000 fathers, enrolled from 50 hospitals across Norway from 1999 to 2008. In 41% of the pregnancies invited, the pregnant women consented to participation. The establishment of MoBa and initial data collection was based on a license from the Norwegian Data Protection Agency and approval from the Regional Committees for Medical and Health Research Ethics. The MoBa cohort is currently regulated by the Norwegian Health Registry Act. The current study was approved by the Regional Committees for Medical and Health Research Ethics.

Children’s height and weight were measured by trained nurses during routine checkups at 6 weeks, at 3, 6 and 8 months and at 1, 1.5, 2, 3, 5, 7 and 8 years of age. These values were recorded in parent-held booklets, and parents were then asked to transcribe these data into the study questionnaires. The data were then processed to compute standardized BMI values at the different timepoints, as previously described^16^. In brief, outliers were removed from growth curves, missing values preceded and followed by at least two measurement points were imputed and BMI values were standardized by sex, taking gestational duration as a covariate and using the generalized additive model for location, scale and shape (GAMLSS) v.5.1-7 (gamlss.com). In all figures presenting temporal trends, the *x* axis represents the square root of age.

Genotyping data were acquired collectively by multiple Norwegian research groups on various genotyping platforms and are managed by the NIPH. Information on the different genotyping batches is available from the NIPH (https://github.com/folkehelseinstituttet/mobagen); this study was conducted using version 1.5 of the genotypes. Quality control of the genotypes was conducted by the PsychGen team; details on the pipeline are available in the respective publication^45^ and code repository (https://github.com/psychgen/MoBaPsychGen-QC-pipeline). GWAS was conducted at each timepoint using Regenie (v.3.2)^46^ as available from Bioconda^47^ and operated using SnakeMake^48^.

### gSEM common factor models

gSEM was conducted using the gSEM R package^20^ (R v.4.0.2; gSEM v.0.0.5). Further details on gSEM and the rationale for the selection of included traits are described in the Supplementary Note.

To predict a single common ‘latent’ factor, childhood adiposity, we used three GWAS datasets as input: AAM from a recent European meta-analysis^21^; MoBa8 (*n* = 26,418), as described above; and recalled comparative adiposity (SAC10) in white European genetic-ancestry participants (*n* = 444,345) from the UK Biobank^34^. The trait (field 1687) is based on the question, ‘When you were 10 years old, compared to average, would you describe yourself as:’ with the following possible answers: ‘thinner’, ‘plumper’, ‘about average’, ‘do not know’ and ‘prefer not to answer’. Participants who responded ‘do not know’ or ‘prefer not to answer’ were excluded unless they subsequently provided an informative response. SAC10 was analyzed as a continuous variable (0, thinner; 1, average; 2, plumper). Association testing was conducted using BOLT LMM^49^.

We then performed a GWAS to identify genetic variants associated with the common factor. We first standardized the summary statistics for each trait using the ‘sumstats’ function, combining the rescaled and aligned data for each trait into a single, listwise-deleted file. We retained insertion–deletion variants and used the ‘commonfactorGWAS’ function to generate effect estimates for each SNP. We used DWLS estimation as recommended by the developers of gSEM^20^.

We excluded heterogeneous GWAS signals, given that some identified common factor signals may have disproportionately large effects on AAM and relatively weak associations with childhood BMI or SAC10. For each SNP, gSEM generates a *Q* index *P* value (*P*_*Q*_), indicating heterogeneity in its associations with the contributing individual traits. Almost all the signals exhibiting significant heterogeneity (*P*_*Q*_ < 5 × 10^−8^) showed highly significant association with AAM but not with any individual childhood adiposity trait (Extended Data Fig. 2). The only exception was rs663129, which showed genome-wide significant associations with SAC10 and MoBa8, but only a nominal (and directionally inconsistent) association with AAM. As rs663129 maps to *MC4R*, a well-known obesity gene, and showed strong associations with all adiposity phenotypes, we retained this signal.

We used linkage disequilibrium score regression (LDSC)^50^ to calculate the genetic correlation (*r*_*g*_) between the ‘childhood adiposity’ common factor and childhood BMI, measured in children 2–10 years of age, available in up to 39,620 European genetic-ancestry participants from the EGG consortium^10^.

### Genetic analyses in ALSPAC

PGS were calculated for participants in the ALSPAC cohort^51,52^. The study initially invited pregnant women resident in Avon, UK, with expected dates of delivery between 1 April 1991 and 31 December 1992 and includes 14,901 children alive at 1 year of age.

We calculated PGS for 7,873 unrelated children with genotyping data. Heights and weights between 4 months and 5 years of age were measured in the study clinic in a 10% subsample called the Children in Focus. For measurements from 7 years of age, the whole cohort was invited to attend the study clinic. BMI measurements were standardized against the British 1990 growth reference for BMI^53^. Fat percentage was calculated as total body fat (measured by dual-energy X-ray absorptiometry) divided by total body weight. The study website contains details of all available data through a data dictionary and variable search tool (http://www.bristol.ac.uk/alspac/researchers/our-data).

A PGS comprising conditionally independent lead SNPs (PGS_LeadSNP) was calculated for each child by summing effect size estimates multiplied by the number of alleles and dividing by the number of variants considered. For time-specific PGSs in MoBa, a set of 152 SNPs was used, corresponding to conditionally independent lead SNPs across all timepoints, and the PGS of a given timepoint was computed using the effect size estimates of these 152 SNPs in the GWAS of MoBa BMI at that timepoint. If a variant was not present in the ALSPAC dataset, a variant in high LD (*r*^2^ > 0.6) was used as a proxy if available.

Genome-wide PGS (PGS_GW) were constructed using LDpred2-auto^54^. SNPs used in the score calculation were limited to those available in the recommended reference panel (HapMap3+), the ALSPAC dataset and the base GWAS. Individual risk scores were computed for children in ALSPAC from the PGS generated by LDpred2-auto using plink2 (ref. ^55^). Associations between individual variants and childhood BMI in ALSPAC were estimated as described for the MoBa cohort above.

### Signal identification and GWAS gene prioritization

To identify independent GWAS signals and prioritize causal genes at the resulting loci, from the timepoint-specific MoBa and gSEM GWAS, we used the G2G pipeline as previously described^21^.

In brief, GWAS summary statistics were filtered at a minor allele frequency (MAF) of >0.1%, and primary genome-wide significant signals were selected in ±1 Mb windows. Secondary signals within these loci were then selected using conditional analyses (GCTA, v.1.93.2)^56^, using an LD reference derived from a random subsample of 25,000 UK Biobank participants. Primary signals were then supplemented with unlinked (*r*^2^ < 5%) secondary signals, whose association statistics did not overtly change in the conditional models.

Signals were mapped to proximal NCBI RefSeq genes, within ±500 kb. Independent signals and their closely linked SNPs (*r*^2^ > 0.8) were annotated if they were coding variants within the identified genes or if they mapped within known enhancers of the identified genes^57^. Signals were also annotated with their nearest gene. Gene-level associations were then determined with Multi-marker Analysis of GenoMic Annotation (MAGMA^58^) by collapsing all coding variants within a gene. Colocalization analyses between the GWAS and eQTL or pQTL data were also performed using SMR-HEIDI (summary data–based MR-heterogeneity in dependent instruments, v.0.68)^59^ and the ABF function in ‘coloc’ (v.5.1.0)^60^. For eQTL analyses, these were performed in all specifically enriched tissues (using LDSC-SEG)^61^, as well as the cross-tissue meta-analyzed GTEx eQTL data (v.7)^62^ and data from the eQTLGen^63^ and Brain-eMeta^64^ studies. Lastly, genes underwent prioritization using the gene-level polygenic priority score (PoPS) method^65^.

This analysis was conducted on each of the timepoint-specific MoBa and gSEM GWAS. To prioritize likely effector genes at each GWAS signal, we prioritized the highest-scoring gene at each locus, if supported by at least two concordant predictors from above.

### Identification of signals and genes across GWAS

Conditionally independent GWAS signals from each of the 11 child BMI timepoints in the MoBa cohort were combined to derive a list of independent signals across childhood, using an LD *r*^2^ matrix. We retained all LD-independent signals (*r*^2^ < 0.05), while prioritizing later MoBa timepoints (that is, 8 years over 7 years, and so on). If a signal at one timepoint was correlated (*r*^2^ > 0.05) with a signal at a different timepoint, the SNP and timepoint with the smaller *P* value was retained.

We further combined signals from the above across-timepoint list with signals from the gSEM GWAS by retaining the MoBa signals and adding any uncorrelated (*r*^2^ < 0.05) gSEM signals (excluding heterogeneous gSEM signals with *P*_Q_ < 5 × 10^−8^). Gene prioritization for each signal was derived from the G2G data in the corresponding GWAS.

In addition to the standard genome-wide significant threshold of *P* < 5 × 10^−8^, we report the number of variants passing a threshold accounting for the 11 GWAS analyses performed in MoBa. Based on phenotypic correlation among BMI measurements, we estimate 4.8 effective independent tests^66^. Therefore, the adjusted multiple-testing threshold is *P* < 1 × 10^−8^ (5 × 10^−8^ / 4.8).

### Clustering of variants into trajectories

Hierarchical clustering was performed to identify trajectories of SNPs with similar effects throughout childhood. We considered the effect of the variants across the MoBa timepoints (including birthweight, even though this was not used for discovery), as well as the re-scaled effect estimates from the gSEM. To assess the effect sizes’ trajectories rather than the absolute difference in effect sizes, correlation was used as a dissimilarity metric. This dissimilarity metric was calculated as (1 − *p*) / 2, where *p* is the correlation between two SNP effect estimates in the 12 GWAS. Complete linkage was used to determine the distance between two clusters. Prior work^16^, using supervised clustering, had suggested four trajectories, and using a cut-point resulting in four trajectories recapitulated the patterns seen there. The homogeneity of trajectories was evaluated using internal cluster validation metrics and trajectory-specific PGS in the independent ALSPAC cohort.

### Phenome-wide association analysis

We generated two scores in the UK Biobank data: one based on the Transient SNPs and one based on the Persistent SNPs. We then identified ICD10 codes in the UK Biobank dataset that had at least 20,000 cases in the complete dataset, based on either the first occurrence data (field category 1712) or the cancer registries data (field category 100092). This produced a list of 78 outcomes that were likely to be sufficiently powered to detect effects. Having filtered the data to a set of unrelated European ancestry individuals (*n* = 367,764), we ran logistic regressions across outcomes accounting for age, sex and the first ten genetic principal components with each of the two scores as the exposure. We then ran an additional model in each case, adjusting for the individual’s BMI at the first visit to the assessment center.

### Univariable and multivariable MR

Given that type 2 diabetes and hypertension were strongly associated with the childhood-derived PGS in the phenome-wide association study, we evaluated the potential differential effects of BMI across time on these outcomes using MR. We selected BMI at 1 year to represent the adiposity peak and the gSEM GWAS to represent late childhood adiposity. To obtain independent MR instruments, we performed more stringent clumping (*r*^2^ = 0.001, ±500 kb). For outcomes, we used the type 2 diabetes GWAS from a previous publication^67^ (*n*_cases_ = 428,452; *n*_controls_ = 2,107,149) and the hypertension GWAS available in the Open GWAS catalog^68^ (ID: ieu-b-5144; *n*_cases_ = 133,680; *n*_controls_ = 329,146).

For multivariable MR, we used the same exposures and outcomes as above but adjusted for adult BMI. For this procedure, we selected a BMI GWAS based on UK Biobank data (10.1038/s41588-018-0144-6) and obtained independent SNPs by clumping. For each exposure, the instrument set consisted of the combined independent SNPs associated with the selected exposure plus those for adult BMI. To reduce redundancy, this merged list was subjected to clumping using the same parameters as before. In both the univariable and multivariable analyses, the inverse-variance weighted method was the primary analytical approach, with MR-Egger and weighted median as sensitivity analyses. All MR analyses were conducted using the MendelianRandomization R package (v.0.10.0)^67,69,70^.

### Analysis of PoE effect and imprinting

We identified and extracted transmitted and non-transmitted alleles for each SNP within parent–offspring trios, classifying them into maternally transmitted, paternally transmitted, maternally non-transmitted and paternally non-transmitted alleles. We used linear regression models to evaluate the association between transmitted and non-transmitted alleles and child BMI at the most significant timepoint in the MoBa cohort, adjusting for covariates such as sex, birth cohort and the top ten principal components to control for population structure. We excluded paternally non-transmitted alleles from the model owing to both the smaller sample size and the reduced biological relevance of these alleles. We estimated the beta model parameters for maternally transmitted and paternally transmitted alleles, as well as their differences (β_MT_ − β_PT_) to quantify PoE. We assessed their statistical significance using a general linear hypothesis test for the null hypothesis (β_MT_ − β_PT_ = 0) using a *t*-distribution. We investigated SNPs located near known imprinted genes (within 100 kb)^71^ to determine whether these regions exhibited PoE. Replication was conducted in up to 123,716 unrelated individuals from the UK Biobank for SAC10 and adult BMI traits using the PoE inference method described previously^29^.

### Biological pathway enrichment analysis

We performed pathway enrichment analysis using g:Profiler^72^ (R package gprofiler2, v.0.2.242). We used pathways from the Gene Ontology database (accessed 06/11/2025) and included pathways with 1,000 genes or fewer, reasoning that these are more biologically specific. Pathway enrichment analyses were performed using the combined set of variants from either MoBa or the gSEM. Pathways with a Bonferroni-corrected *P* < 0.05 were considered enriched.

As many reference pathways are based on overlapping data, we aggregated the childhood adiposity-associated pathways based on shared genes. We used a ‘complete’ clustering algorithm and a custom distance calculated as 1 − (proportion of the overlap between any two pathways relative to the pathway with the smaller size). Therefore, between two given pathways, a value of zero indicates that all intersecting genes in the pathway with fewer genes are also present in the other pathway. To define clusters, we chose an arbitrary cut-off value of 0.5, which indicates that pairs of pathways share 50% or more genes.

### Child–adult effect comparisons

We used GWAS data on adult BMI from the GIANT consortium^6^ to explore which of our gSEM GWAS signals exert larger effects on adiposity in childhood compared to adulthood. We categorized child adiposity signals that had no association with adult BMI (*P* > 0.05) or a nominal but directionally non-concordant association with adult BMI (*P* < 0.05). We also rescaled the gSEM common factor effect estimates to a BMI scale standardized for age and sex, similar to the cross-sectional MoBa GWAS and adult BMI GWAS. To do so, we regressed the effect estimates of the 152 SNPs discovered in the MoBa GWASs against their effect estimates on the gSEM common factor and used the slope of this association (0.5365501) as a scaling factor (that is, (gSEM common factor effect estimate) / 0.5365501). We then performed a look-up of all independent child adiposity signals in the adult BMI GWAS, using the best available proxy (*r*^2^ ≥ 0.4) for any child signal unavailable in the adult BMI GWAS. We aligned all SNPs to the child adiposity-increasing allele and calculated the difference in effect estimates between childhood and adulthood (that is, β_child_ − β_adult_). We considered the top 5% of signals in the distribution of these effect differences to have disproportionately larger effects on child BMI than adult BMI.

### Integration of human hypothalamic cell data

We used the Human HYPOMAP data^32^ to identify hypothalamic cell populations specifically enriched for genes harboring either childhood or adult adiposity-associated variants. For adult adiposity, we used the GIANTmeta-analysis^6^ (*n*_max_ = 806,834), and for child adiposity, we used the filtered gSEM summary statistics as described above. As these were genome-wide data, we removed signals exhibiting significant heterogeneity (*P*_*Q*_ < 5 × 10^−8^) and all other variants in any LD with them (*r*^2^ > 0.05). We then used cell-type specificity matrices, generated with CELLEX (https://github.com/perslab/CELLEX) as previously described^32^, in CELL-type Expression-specific integration for Complex Traits (CELLECT) with MAGMA. For each trait, CELLECT-MAGMA (v.1.3.0) was run with default parameters across the 452 hypothalamic cell populations with a multiple-test corrected significance threshold of *P* < 0.05 / 452.

To identify childhood adiposity genes that contributed strongly to cell-type classification (informative marker genes), we defined genes that were both high-confidence childhood adiposity genes identified through G2G and in the top 100 genes (equivalent to the ~99.5^th^ percentile) for cell-type specificity within each identified cell cluster. This approach was used to highlight childhood adiposity genes that are specifically expressed in the childhood adiposity enriched ARC or MAM cell populations (Supplementary Data 24).

### Gene burden testing of rare variants in WGS data

WGS data in up to 490,640 participants from UK Biobank were accessed and processed as described previously^73^. In brief, 1,037,556,156 SNPs and 101,188,713 insertions and deletions were called using Graphtyper^74^. Multi-allelic sites were split and normalized in Hail (v.0.2)^75^, and variants with low quality control metrics or high missingness were filtered out. Variants were initially annotated using the Ensembl Variant Effect Predictor (VEP^76^, v.108.2) to identify missense and PTVs (stop-gained, splice acceptor and splice donor consequences), with the LOFTEE^77^ plugin for the identification of high-confidence and low-confidence PTVs. We also used REVEL^78^ (rare exome variant ensemble learner) scores to annotate missense variants. We tested associations with two variant-collapsing masks: rare (MAF < 0.1%) high-confidence PTVs and rare (MAF < 0.1%) predicted deleterious missense variants (defined by a REVEL score of ≥0.7).

We tested two outcomes: SAC10 and adult BMI (kg m^−2^ from field 23104) using BOLT-LMM (v.2.4.1)^49^. Gene burden tests were performed by generating a dummy genotype file in which each gene–mask combination was coded as a single variable. We then coded individuals with 1+ qualifying variants for each mask within a gene as heterozygous. We used the BOLT-LMM’lmmInfOnly’ flag and the following covariates: age, age^2^, sex, age × sex, the first 20 principal components and the WGS release batch. We included all samples without restricting by ancestry to maximize sample size, and we only looked at genes with at least 100 carriers of qualifying variants. Samples with withdrawn consent or missing phenotypes and covariates were excluded, resulting in 481,137 and 479,615 samples remaining for BMI and SAC10, respectively.

### Related trait WGS associations

For all identified gene burden associations with SAC10, we performed similar gene burden association testing for predefined obesity-related phenotypes. We used genetic data from the previous release of whole-exome sequencing data in UK Biobank, which included up to 450,000 participants.

We selected eight phenotypes, including SAC10, adult BMI and body fat percentage data from UK Biobank field 23099. For all phenotypes, data from subsequent visits were used if missing for a given instance. Comparative height at age 10 (HAC10, field 1697) was recoded as 0: ‘shorter’; 1: ‘about average’; and 2: ‘taller’ and was analyzed as a continuous variable. ‘Do not know’ or ‘prefer not to answer’ responses were excluded. For waist-to-hip ratio adjusted for BMI data, the waist-to-hip ratio was calculated using data from fields 48 and 49. Values greater than four standard deviations from the mean were excluded before adjusting for BMI (field 21001). Type 2 diabetes was derived as previously described^53^ and using data from fields 4041, 10844, 2443, 6177, 6153, 20002, 20003, 41202, 41204, 40001 and 40002. Lean mass index was calculated using whole-body fat-free mass, derived from bioelectrical impedance (field 23101) as the numerator, and height squared in cm (field 50) as the denominator. Hand grip strength was derived from the average of fields 46 and 47. The multiple-testing threshold (*P* <3.5 × 10^−4^; derived as 0.05 / (3 × 6 × 8)) was calculated correcting for eight phenotypes, six identified genes and three variant-collapsing masks.

We conducted gene burden tests against these eight outcomes, as previously described^79^, in up to 419,692 individuals of European ancestry from UK Biobank^80^ and collapsed rare variants into three masks: high-confidence PTVs, missense variants with a REVEL score of ≥0.7 and missense variants with a CADD score of ≥25.

### Reporting summary

Further information on research design is available in the Nature Portfolio Reporting Summary linked to this article.

## Online content

Any methods, additional references, Nature Portfolio reporting summaries, source data, extended data, supplementary information, acknowledgements, peer review information; details of author contributions and competing interests; and statements of data and code availability are available at https://doi.org/10.1038/s41588-026-02772-y.

## Supplementary information

Supplementary Information Supplementary Note.

Reporting Summary

Peer Review file

Supplementary Data 1 Supplementary Data 1–26.

## Data Availability

Data from the Norwegian Mother, Father and Child Cohort Study used in this study are managed by the Norwegian Institute of Public Health and can be made available to researchers, provided approval from the Regional Committees for Medical and Health Research Ethics (REC), compliance with the EU General Data Protection Regulation (GDPR) and approval from the data owners. The consent given by the participants does not open for storage of data at the individual level in repositories or journals. Researchers who request access to datasets for replication should apply through helsedata.no. Access to datasets requires approval from the Regional Committee for Medical and Health Research Ethics in Norway and an agreement with MoBa. Summary statistics are available for download at https://doi.org/10.5281/zenodo.19608167. The UK Biobank phenotype and genetic data described here are publicly available to registered researchers through the UK Biobank data access protocol. Information about registration for access to the data is available at https://www.ukbiobank.ac.uk/enable-your-research/apply-for-access. All bona fide researchers can apply to use ALSPAC data for health-related research that is in the public interest. Information regarding the ALSPAC cohort and data access is available at https://www.bristol.ac.uk/alspac/researchers/our-data.

## Code availability

No custom code was used in this work.
