# Supplementary material for: Genome-wide mapping of common and rare variant effects on adiposity across childhood
Source: Nat Genet. 2026 Sep 25;58(11):2794–803. doi: 10.1038/s41588-026-02772-y (PMC13754613; doi:10.1038/s41588-026-02772-y)
Supplement: Supplementary file 1 [file 41588_2026_2772_MOESM1_ESM.pdf]

nature genetics

Supplementary information

https://doi.org/10.1038/s41588-026-02772-y

Genome-wide mapping of common and rare variant effects on adiposity across childhood

In the format provided by the authors and unedited

Supplementary Note

Contents

Generation of genomic SEM common factor models for childhood adiposity 2

Rationale for age at menarche (AAM) as a childhood adiposity trait 3

Rationale for comparative body size at age 10 years (SAC10) as a childhood adiposity trait 3

Correlations between individual traits 3

Genomic SEM model fit 5

GWAS signals for each genomic SEM common factor 6

Exclusion of heterogenous GWAS signals 10

Validity of GWAS signals for childhood adiposity common factors 11

Demonstrating the impact of heterogenous signals (Q SNPs) 12

Summary 13

References 14

Generation of genomic SEM common factor models for childhood adiposity

Genomic SEM (gSEM) is a 2-step structural equation modelling approach. First, the empirical genetic covariance matrix and its sampling covariance matrix of included traits are estimated using an extended version of LD-score regression[1], which takes GWAS summary statistics as input. The regression accounts for potential sample overlap. Second, the user specifies a structural equation model that can omit or include the effects of individual SNPs. Parameters are then estimated by minimising the discrepancy between the model-implied genetic covariance matrix and the empirical covariance matrix obtained in the first step. These analyses were conducted using the gSEM R package[2] (R version 4.0.2, gSEM version 0.0.5).

For each input GWAS, we performed quality control, only including SNPs with minor allele frequency (MAF) >0.1% and imputation information (INFO) score >0.5. As the gSEM software runs in R, which can represent numbers as non-zero only if $\geq 5\times10^{-324}$, we reset the *P*-values of SNPs which were below that value, to this value. Summary statistics for each trait were reformatted using the “munge” function. We then ran multivariable LD-Score regression using the “ldsc” function using LD scores and weights for populations of European genetic ancestry[1]. Third, we used the estimated genetic covariance matrix and its associated sampling covariance matrix from the step above to fit a common factor model using Diagonally Weighted Least Squares (DWLS) estimation by applying the “commonfactor” function. Finally, we used DWLS estimation as it is recommended over maximum likelihood estimation by the developers of gSEM.

Rationale for age at menarche (AAM) as a childhood adiposity trait

We recently reported that 45% of AAM-lowering GWAS signals have a positive effect on weight during early childhood, before the age range for puberty onset, indicating that many signals impact AAM indirectly, via primary effects on early weight gain (Kentistou *et al.*[3]). That analysis was based on a subset of the MoBa study.

Figure 1 | Clustering of AAM-lowering GWAS signals by early weight trajectories in MoBa subsample – data from Kentiostou *et al* 2024.

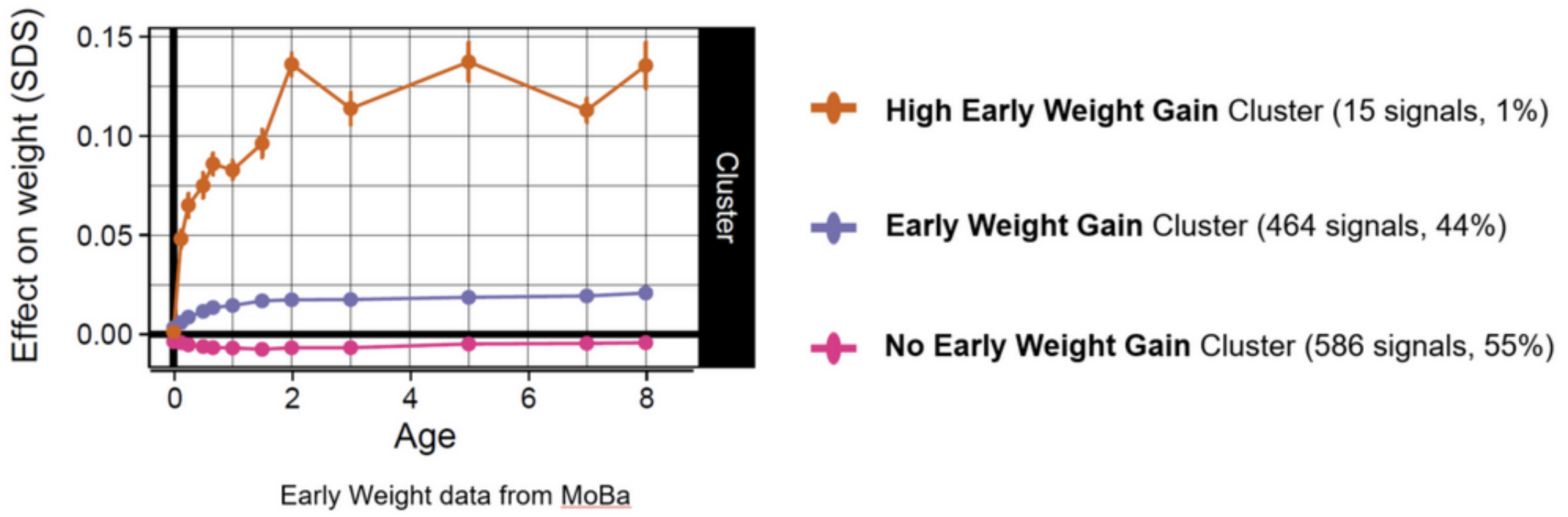

Rationale for comparative body size at age 10 years (SAC10) as a childhood adiposity trait

The validity of "comparative body size at age 10" (SAC10) for genetic associations has been well documented by ourselves and others, indicated by high genetic correlation (Rg=0.85 to 0.94) with objectively measured childhood BMI[4,5].

Correlations between individual traits

We observed a strong positive genetic correlation between SAC10 and childhood BMI (MOBA7: R_g=0.88; MOBA8: R_g=0.85) consistent with previous reports. We also observed a moderately strong inverse genetic correlation between each childhood adiposity trait and AAM (R_g:-0.36 to -0.39), similar to the reported genetic correlation between AAM and adult BMI (**Figure 2**). These genetic correlations supported the rationale to run our pre-specified genomic SEM models assuming the presence of a single "childhood adiposity" common factor.

Figure 2 | Heatmap of genetic correlations among AAM (age at menarche), SAC10 (comparative body size age 10), MOBA7 (childhood BMI age 7) and MOBA8 (childhood BMI age 8). Correlations were estimated using multivariate LDSC.

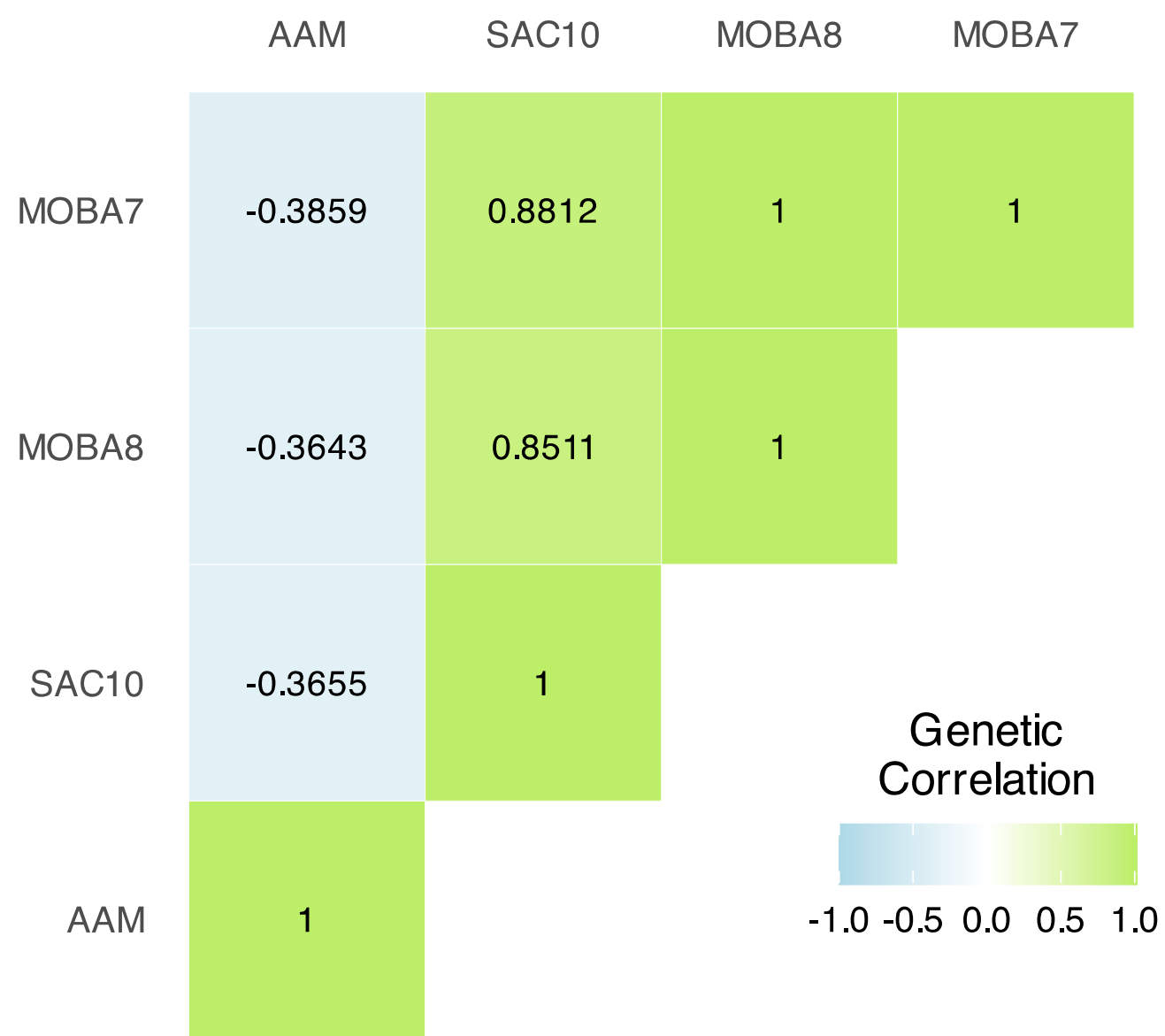

We explored 4 different gSEM models (see below) and assessed how well each model fit the assumption of a single "childhood adiposity" common factor using the Comparative Fit Index (CFI) and standardised root mean square residual (SRMR); these are the only 2 indexes computed by the genomic SEM software when the minimum of 3 traits are included. The CFI indicates the extent to which the proposed model fits better than a model that allows all phenotypes to be heritable but genetically uncorrelated. CFI values range from 0 to 1, with values >0.9 considered an acceptable model fit. SRMR is calculated as the standardised root mean squared difference between the model-implied and observed correlations in the model-implied and empirical covariance matrixes. Low SRMR values <0.1 indicate small discrepancy and acceptable model fit[6]. In some cases, the standardised factor loading for an input trait exceeds 1 (a "Heywood case") and the indicator variable has a negative residual variance. In such cases, it is assumed that the true loading lies very close to 1 and, to avoid the inclusion of impossible values, we imposed a model constraint to keep the residual variance above 0, as advised by the developers of gSEM[2].

1. AAM, SAC10, MOBA7
2. AAM, SAC10, MOBA8
3. AAM, SAC10, MOBA7, MOBA8.
4. SAC10, MOBA7, MOBA8.

Genomic SEM model fit

The model fit for the first three models was good**.** Model 4 generated a 'Heywood case' where the standardised factor loading from MOBA7 exceeded 1 and thus also had a negative residual variance. If we imposed additional model constraints to keep the residual variance >0, the degrees of freedom for Model 4 dropped to 0, and no model fit indices could be calculated.

Genomic SEM factor loadings: Model results showed strong positive loadings for all adiposity traits across all four models (0.87-1.00) (**Figure 3**). In Models 1, 3, and 4, MOBA7 showed the strongest factor loading (0.96 to 0.99). In Model 2, SAC10 and MOBA8 showed similarly strong factor loading (both 0.92). AAM showed moderate negative loadings (-0.40) on Models 1,2 and 3, consistent with the genetic correlations between individual traits (**Figure 2**).

Figure 3 | Common factor analysis of the genetic "childhood adiposity" common factor (F1) for **a**) Model 1, **b**) Model 2, **c**) Model 3 and **d**) Model 4. Standardised indicator loading estimates of each individual trait on the common factor are displayed (AAM=age at menarche, SAC10=comparative body size age 10, MOBA7=childhood BMI age 7, MOBA8=childhood BMI age 8). The "u" variables indicate residual variances in each trait.

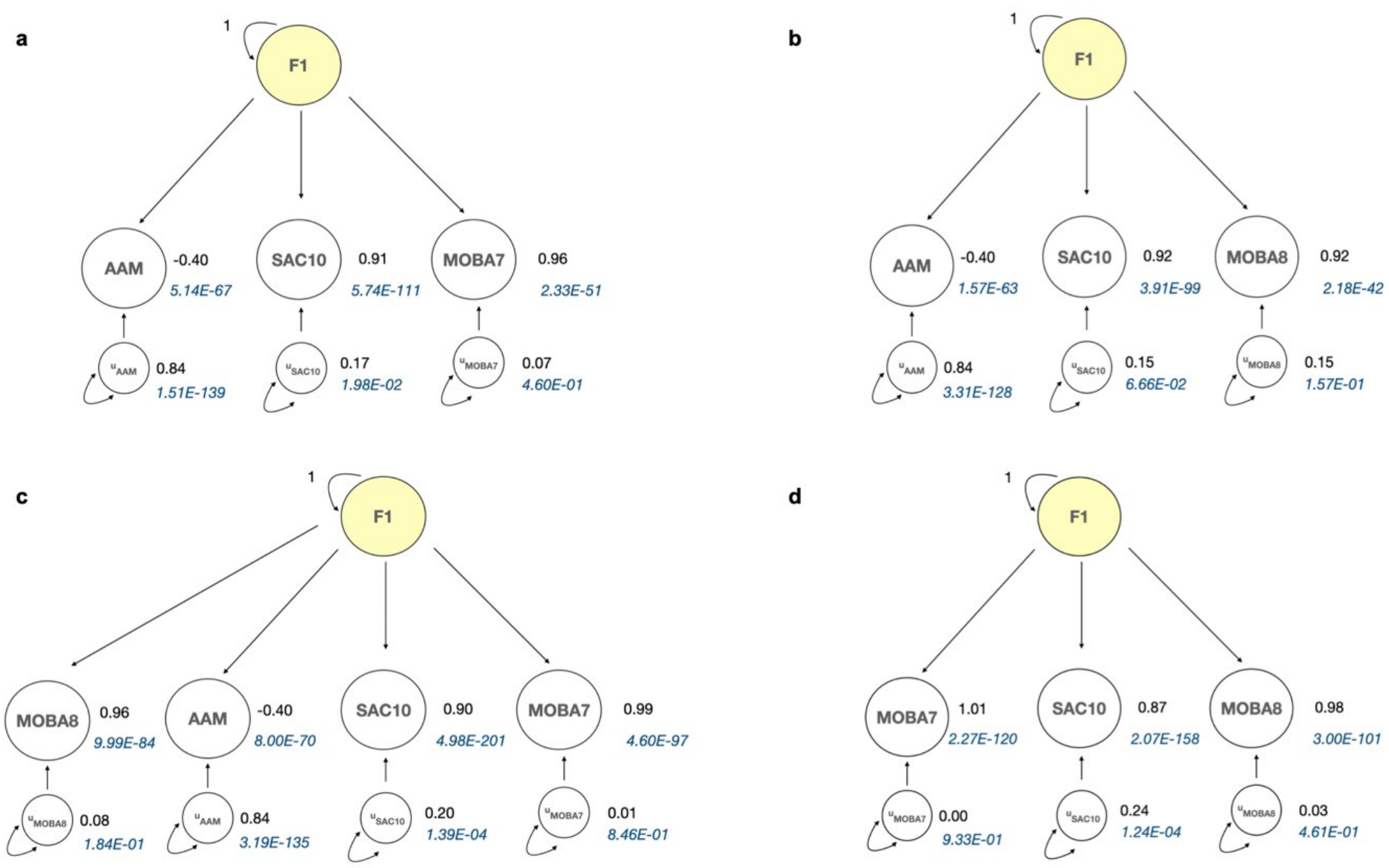

GWAS signals for each genomic SEM common factor

We incorporated genome-wide SNP effects into the genetic covariance matrix and the sampling covariance matrix to estimate the effect of each SNP on the "childhood adiposity" common factor in each model.

- Model 1 identified 510 independent GWAS signals ($p<5\times10^{-8}$) for its "childhood adiposity" common factor. Of the 510 loci, 66 (13%) did not reach genome-wide significance for association with any contributing individual trait (**Figure 4a**).
- Model 2 identified 643 independent GWAS signals. Of these, 135 (21%) did not reach genome-wide significance for association with any contributing individual trait (**Figure 4b**).
- Model 3 identified 539 independent GWAS signals. Of these 106 (20%) did not reach genome-wide significance for association with any contributing individual trait (**Figure 4c**).
- Model 4 identified 258 independent GWAS signals. Of these 35 (14%) did not reach genome-wide significance for association with any contributing individual trait (**Figure 4d**).

Following the previous examples of gSEM[7] we estimated the expected sample size for the common factor GWAS using the equation:

$$N_{eff} \approx \frac{1}{m} \sum_{MAF=a}^{b} n_j$$

Where n_j for each of the m SNPs is estimated as:

$$n_j = \frac{(\frac{Z_j}{\beta_j})^2}{\sigma_j^2}$$

We restricted MAFs to between 10% and 40% to produce stable sample size estimates as recommended by the developers of gSEM.

Model 2 produced the most GWAS signals and the largest effective sample size. This is because the trait with the highest factor loading in this model (SAC10, slightly larger than MOBA8) had a much larger sample size and thus better-powered effect estimates than MOBA7, which had the highest factor loading in Models 1, 3 and 4.

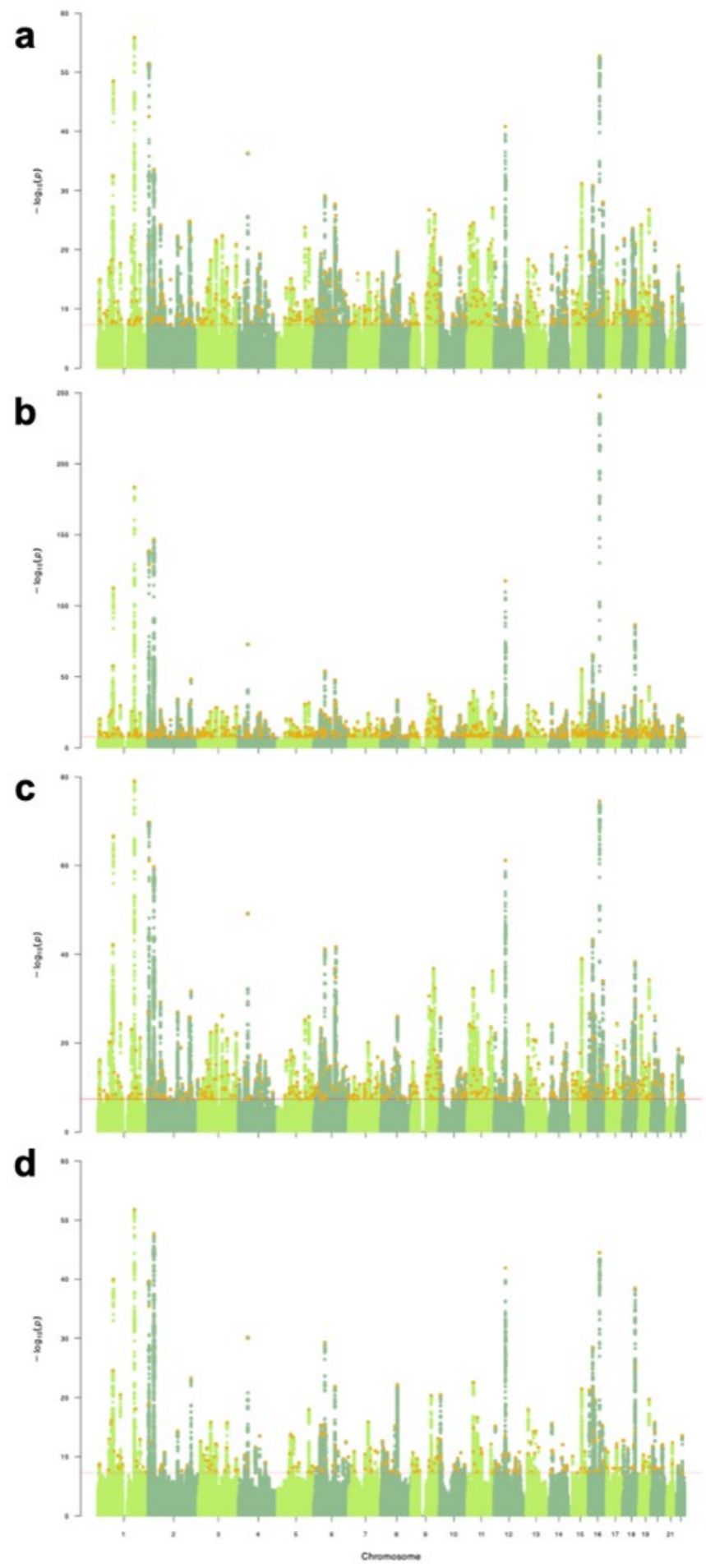

Figure 4 | Manhattan plots showing GWAS results for the "childhood adiposity" common factors generated by Models 1-4 (**a**-**d**). Red horizontal lines indicate genome-wide significance ($p<5\times10^{-8}$). Independent genome-wide significant loci are highlighted in red.

QQ plots of GWAS results for the common factors from all models showed slightly higher genomic inflation factors (λ_{GC}=1.69-2.04) compared to those for each individual trait (AAM λ_{GC}=1.72, SAC10 λ_{GC}=1.43, MOBA7 λ_{GC}=1.14, MOBA8 λ_{GC}=1.12) (**Figure 5**). Reassuringly, all LDSC intercepts were close to one, indicating that λ_{GC} values reflect polygenicity rather than uncontrolled inflation.

Figure 5 | Quantile-quantile (QQ) plots of p-values from GWAS for each "childhood adiposity" common factor generated by Models 1-4 (**a**-**d**). Expected -log10 p-values are those expected under the null hypothesis.

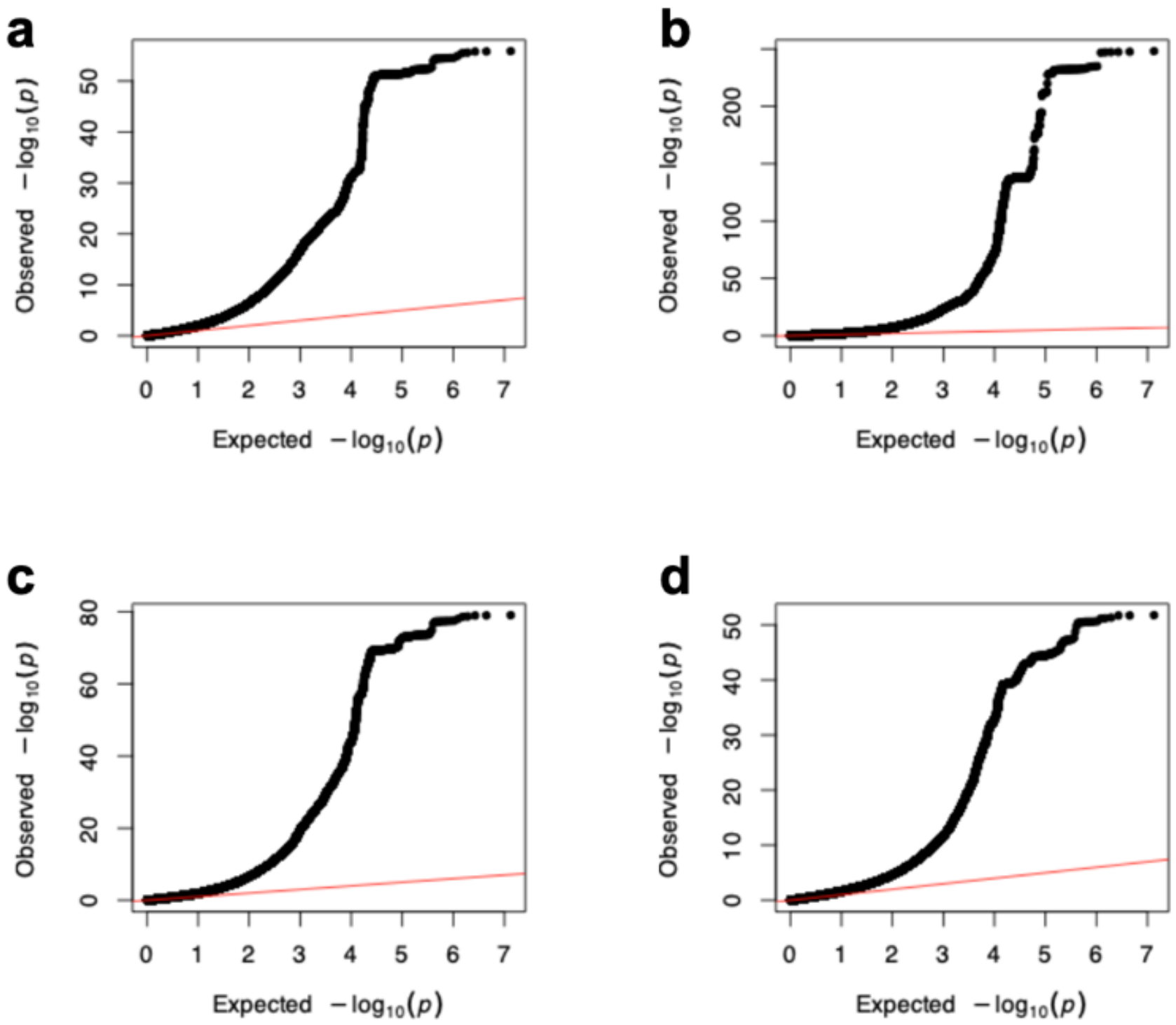

Furthermore, lead SNPs for each “childhood adiposity” common factor in Models 1-4, but which did not show genome-wide significant association ($P>5\times10^{-8}$) with any individual contributing trait, showed near but sub-threshold genome-wide significant association with individual traits (**Figure 6**). This confirms that genomic SEM was unlikely to generate artefactual SNP associations, e.g. due to collider effects.

Figure 6 | Histograms of -log10 p-values for lead SNPs for each “childhood adiposity” common factor in Models 1-4 (**a**-**d**) but which did not show genome-wide significant association ($P>5\times10^{-8}$) with any individual contributing trait. The vertical red line indicates genome-wide significance. -log10(P)=-log10 p-value for common “childhood adiposity” factor; AAM=age at menarche; SAC10=comparative body size age 10; MOBA7=childhood BMI age 7; MOBA8=childhood BMI age 8.

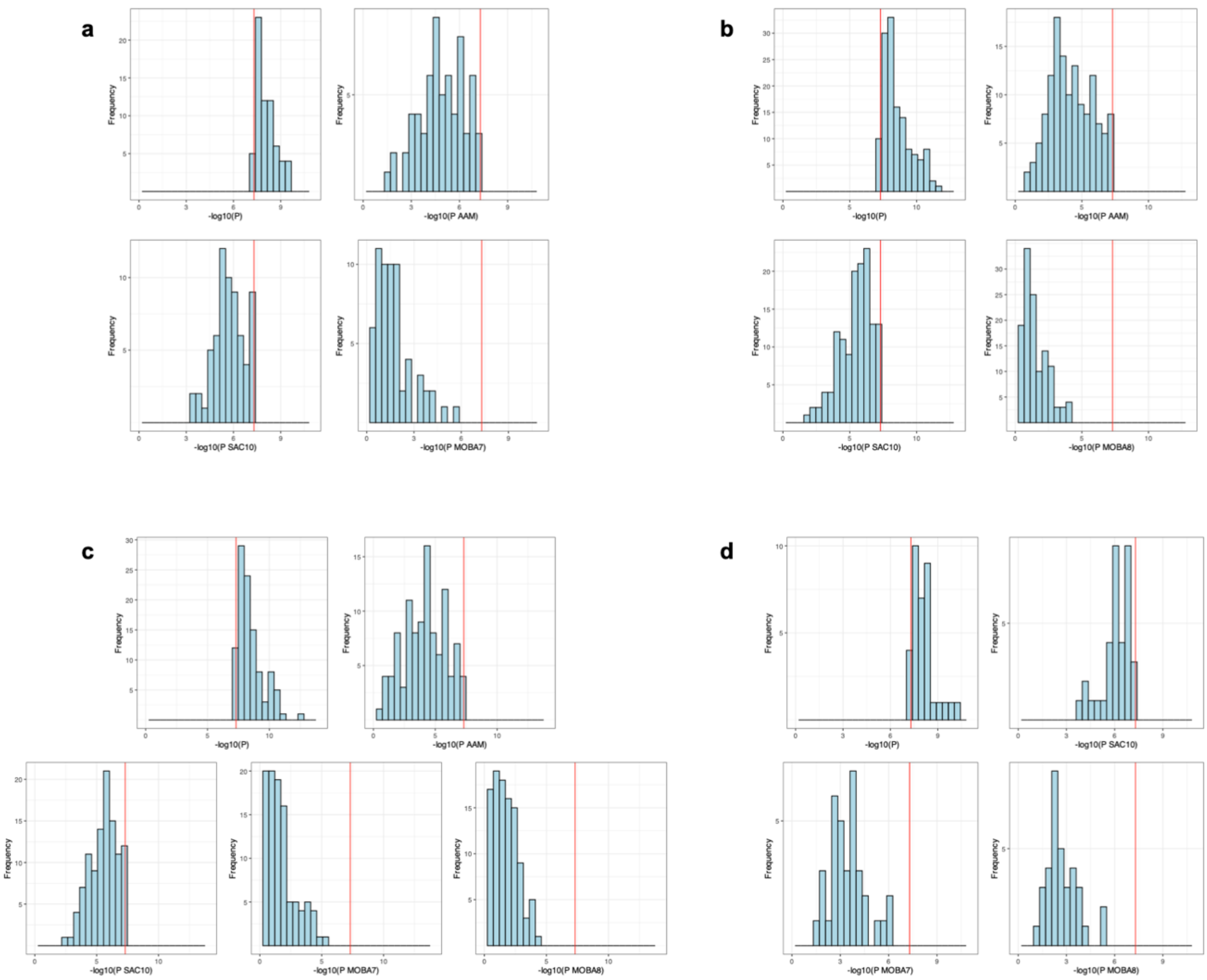

Exclusion of heterogenous GWAS signals

Some identified common factor signals may have disproportionately large effects on AAM and have relatively weak association with childhood BMI or SAC10. For each SNP, genomic SEM generates a Q index *p*-value (p_Q), indicating heterogeneity in its associations with the contributing individual traits. ~20% of signals across Models 1-3 (but none in Model 4) showed significant heterogeneity (p_Q<5×10^{-8}); almost invariably such signals showed highly significant association with AAM but not with any individual childhood adiposity trait (**Figures 7**). The only exception was rs663129 (in Models 2 and 3), which showed genome-wide significant associations with SAC10, MOBA7 and MOBA8, but only nominal (and directionally inconsistent) association with AAM (**Figures 7b, 7c**). As rs663129 maps to *MC4R*, a known obesity gene, and showed strong associations with all adiposity phenotypes, we retained this signal. By contrast, other heterogenous SNPs mapped to well-known puberty genes such as *KISS1* and *TACR3* that are reported to be disrupted in monogenic disorders of puberty as well as several retinoic acid receptors involved in retinoic acid signalling (*RORB*, *RORA* and *RXRG*) and have previously been identified in GWAS for AAM. After excluding all heterogenous SNPs (except rs663129):

- **Model 1 (AAM, SAC10, MOBA7) produced 400 signals** after excluding 110 heterogeneous signals
- **Model 2 (AAM, SAC10, MOBA8) produced 526 signals** after excluding 117 heterogeneous signals
- **Model 3 (SAC10, MOBA7, MOBA8, AAM) produced 440 signals** after excluding 99 heterogeneous signals
- **Model 4 (SAC10, MOBA7, MOBA8) produced 258 signals** after excluding no heterogeneous signals

Figure 7 | Scatterplots comparing common factor and individual trait associations (Z-scores) for the GWAS signals identified in Models 1-3 (**a**-**c**). Heterogenous signals (pQ<5×10^{-8}) are highlighted in blue. The one retained heterogenous signal, rs663129, is labelled. AAM=age at menarche, SAC10=comparative body size age 10, MOBA7=childhood BMI age 7, MOBA8=childhood BMI age 8.

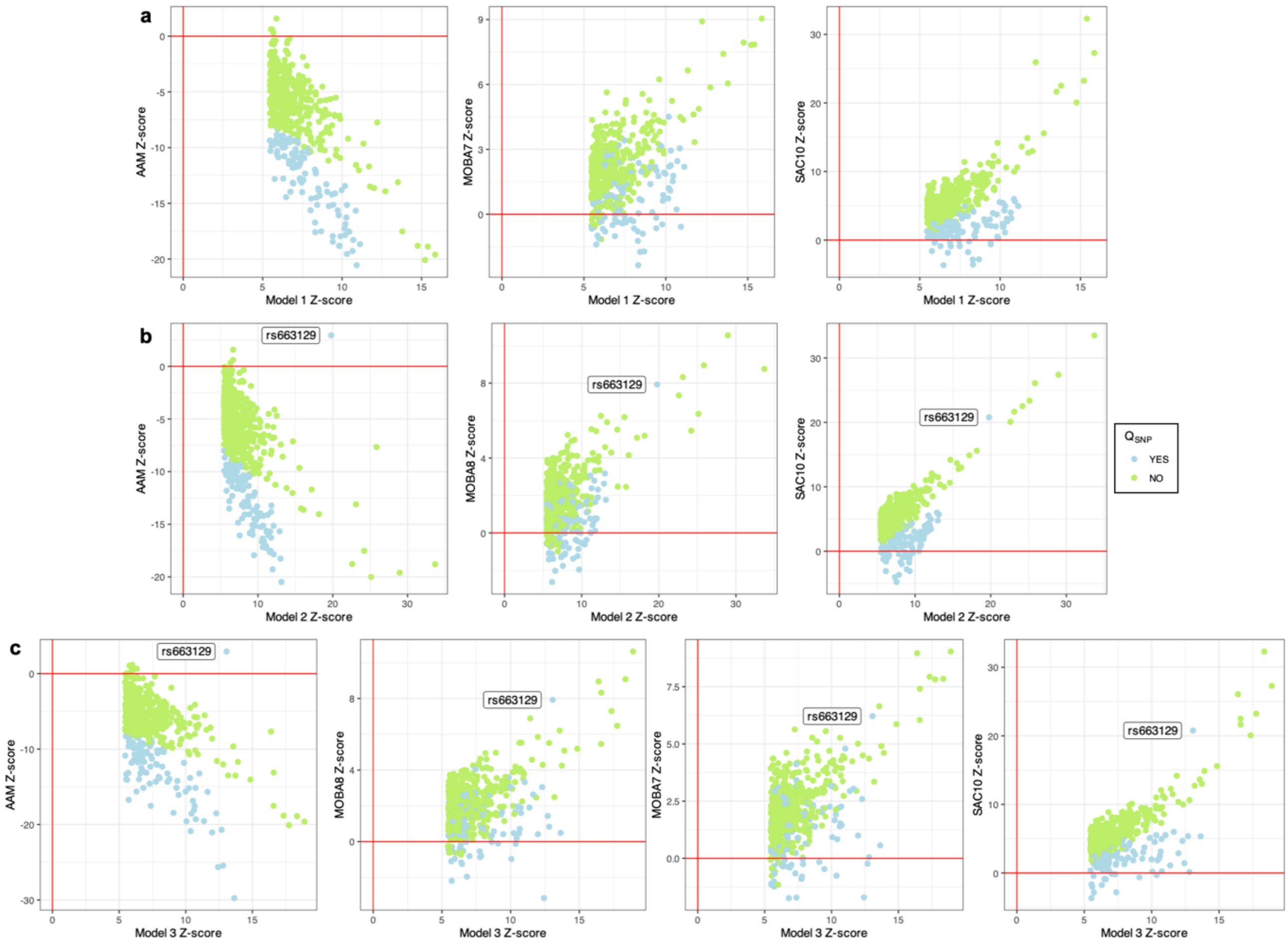

Validity of GWAS signals for childhood adiposity common factors

Genetic correlations with childhood BMI: The common factors from all four models showed high genetic correlations with childhood BMI from independent EGG data. Model 4 showed the highest genetic correlation with childhood BMI (R_g=0.97, 95% CI: 0.86-1.00) although its confidence intervals overlapped with the estimates from Models 1-3 (R_g=0.85-0.87).

Directional concordance with childhood BMI: After exclusion of heterogenous signals, GWAS signals for the common factors from all four models showed high directional concordance with effect estimates with childhood BMI (**Table 1**). Signals from Model 4 showed the highest directional concordance (97%), and concordance was also high for signals from Models 1-3 (91-93%).

Concordance with adult BMI: Using data on adult BMI from the UK Biobank, we observed consistent moderate positive genetic correlations between all common factors and adult BMI (R_g=0.54-0.57). GWAS signals for the common factors from all four models showed consistently high directional concordance in effect estimates with adult BMI (93-96%, **Table 2**).

Demonstrating the impact of heterogenous signals (Q SNPs)

Compared to inclusion of all independent signals for each model, after filtering out heterogenous signals (Q-SNPs) with disproportionately large effects on AAM compared to their effects on childhood BMI or SAC10, the concordance of the direction of effect of loci with measured childhood BMI increased by 5 to 6% across models 1-3 to 91-93% (**Table 1**). Similarly, the concordance of the direction of effect of loci with adult BMI increased by 3 to 5% across models 1-3 to 93-94% (**Table 2**).

Table 1 | Directional concordance of signals for the "childhood adiposity" common factors and measured childhood BMI (from EGG[8]).

		**Childhood BMI**					
		**Concordance of the direction of effect (all SNPs)**			**Concordance of the direction of effect (excluding Q_{SNPs})**		
**Model**	**Traits**	**%**	**N**	**P**	**%**	**N**	**P**
1	AAM, MOBA7, SAC10	86	434/507	8.0×10^{-64}	92	366/397	4.5×10^{-74}
2	AAM, MOBA8, SAC10	85	544/638	3.2×10^{-78}	91	472/520	3.6×10^{-89}
3	AAM, MOBA7, MOBA8, SAC10	88	470/535	4.8×10^{-77}	93	404/435	3.1×10^{-81}
4	MOBA7, MOBA8, SAC10	97	247/258	7.1×10^{-63}	N/A	N/A	N/A

Table 2 | Directional concordance of signals for the "childhood adiposity" common factors and measured adult BMI (from UK Biobank).

		**Adult BMI**					
		**Concordance of the direction of effect (all SNPs)**			**Concordance of the direction of effect (excluding Q_{SNPs})**		
**Model**	**Traits**	**%**	**N**	**P**	**%**	**N**	**P**
1	AAM, MOBA7, SAC10	90	457/510	1.5×10^{-81}	94	377/400	5.9×10^{-84}
2	AAM, MOBA8, SAC10	88	564/643	1.7×10^{-91}	93	487/525	6×10^{-101}
3	AAM, MOBA7, MOBA8, SAC10	91	489/539	7.4×10^{-92}	94	413/439	4.4×10^{-91}
4	MOBA7, MOBA8, SAC10	96	248/258	6.8×10^{-61}	N/A	N/A	N/A

Model; model number, Traits; as included in the model (AAM = age at menarche, SAC10 = comparative body size age 10, MOBA7 = childhood BMI age 7, MOBA8 = childhood BMI age 8), Concordance of the direction of effect (all SNPs/excluding QSNPs): %; percentage of loci with a concordant direction of effect between tested model and adult BMI, N; number of loci with a concordant direction of effect between tested model and adult BMI, P; p-value for a binomial sign test.

Summary

Leveraging AAM data increased the power to detect signals for a single “childhood adiposity” common factor (400-526 signals in Models 1-3 compared to 258 signals in Model 4). We selected **Model 2 (AAM, SAC10, MOBA8)** as it was the best-powered model, as well as performing well across all quality control and validation metrics.

References

1. Bulik-Sullivan, B. K. *et al.* LD Score regression distinguishes confounding from polygenicity in genome-wide association studies. *Nat. Genet.* **47**, 291–295 (2015).
2. Grotzinger, A. D. *et al.* Genomic structural equation modelling provides insights into the multivariate genetic architecture of complex traits. *Nat. Hum. Behav.* **3**, 513–525 (2019).
3. Kentistou, K. A. *et al.* Understanding the genetic complexity of puberty timing across the allele frequency spectrum. Preprint at https://doi.org/10.1101/2023.06.14.23291322 (2023).
4. Richardson, T. G., Sanderson, E., Elsworth, B., Tilling, K. & Davey Smith, G. Use of genetic variation to separate the effects of early and later life adiposity on disease risk: mendelian randomisation study. *BMJ* m1203 (2020) doi:10.1136/bmj.m1203.
5. Kaisinger, L. R. *et al.* Large-scale exome sequence analysis identifies sex- and age-specific determinants of obesity. *Cell Genomics* **3**, 100362 (2023).
6. Hu, L. & Bentler, P. M. Cutoff criteria for fit indexes in covariance structure analysis: Conventional criteria versus new alternatives. *Struct. Equ. Model. Multidiscip. J.* **6**, 1–55 (1999).
7. Mallard, T. T. *et al.* Multivariate GWAS of psychiatric disorders and their cardinal symptoms reveal two dimensions of cross-cutting genetic liabilities. *Cell Genomics* **2**, 100140, S2666-979X(22)00073–8 (2022).
8. Vogelezang, S. *et al.* Novel loci for childhood body mass index and shared heritability with adult cardiometabolic traits. *PLOS Genet.* **16**, e1008718 (2020).
