# Supplementary material for: Genome-wide mapping of common and rare variant effects on adiposity across childhood
Source: Nat Genet. 2026 Sep 25;58(11):2794–803. doi: 10.1038/s41588-026-02772-y (PMC13754613; doi:10.1038/s41588-026-02772-y)
Supplement: Supplementary file 2 [file 41588_2026_2772_MOESM2_ESM.pdf]

nature portfolio

Corresponding author(s): Ken K Ong & Stefan Johansson

Last updated by author(s): Apr 21, 2026

Reporting Summary

Nature Portfolio wishes to improve the reproducibility of the work that we publish. This form provides structure for consistency and transparency in reporting. For further information on Nature Portfolio policies, see our Editorial Policies and the Editorial Policy Checklist.

Statistics

For all statistical analyses, confirm that the following items are present in the figure legend, table legend, main text, or Methods section.

n/a	Confirmed	
☐	☒	The exact sample size (*n*) for each experimental group/condition, given as a discrete number and unit of measurement
☐	☒	A statement on whether measurements were taken from distinct samples or whether the same sample was measured repeatedly
☐	☒	The statistical test(s) used AND whether they are one- or two-sided<br>*Only common tests should be described solely by name; describe more complex techniques in the Methods section.*
☐	☒	A description of all covariates tested
☐	☒	A description of any assumptions or corrections, such as tests of normality and adjustment for multiple comparisons
☐	☒	A full description of the statistical parameters including central tendency (e.g. means) or other basic estimates (e.g. regression coefficient) AND variation (e.g. standard deviation) or associated estimates of uncertainty (e.g. confidence intervals)
☐	☒	For null hypothesis testing, the test statistic (e.g. *F*, *t*, *r*) with confidence intervals, effect sizes, degrees of freedom and *P* value noted<br>*Give P values as exact values whenever suitable.*
☒	☐	For Bayesian analysis, information on the choice of priors and Markov chain Monte Carlo settings
☐	☒	For hierarchical and complex designs, identification of the appropriate level for tests and full reporting of outcomes
☐	☒	Estimates of effect sizes (e.g. Cohen's *d*, Pearson's *r*), indicating how they were calculated

Our web collection on statistics for biologists contains articles on many of the points above.

Software and code

Policy information about availability of computer code

Data collection	No specific software was used in data collection.
Data analysis	Analyses were conducted using the following software: regenie v3.2, BOLT-LMM v2.4.1, PLINKv2.0, GCTA v1.93.2, PoPS v0.2, MAGMA v1.09, SMR-HEIDI v0.68, Graphtyper, Hail v0.2, Ensembl VEP v108.2, CELLEX v.1.2.2, CELLECT v1.3.0, and R v3.6.1-4.3.1 with the packages gSEM v0.0.5, gamlss v5.1-7, gprofiler2 v0.2.242, coloc v5.1.0, ldsc v1.0.1, bigsnpr v1.12.2.

For manuscripts utilizing custom algorithms or software that are central to the research but not yet described in published literature, software must be made available to editors and reviewers. We strongly encourage code deposition in a community repository (e.g. GitHub). See the Nature Portfolio guidelines for submitting code & software for further information.

Data

Policy information about availability of data

All manuscripts must include a data availability statement. This statement should provide the following information, where applicable:

- Accession codes, unique identifiers, or web links for publicly available datasets
- A description of any restrictions on data availability
- For clinical datasets or third party data, please ensure that the statement adheres to our policy

The full summary statistics of all GWAS conducted in this study will be available for download at Summary statistics are available for download here: https://doi.org/10.5281/zenodo.19608167. We refer to the manuscript for information on how to access previously published data.

Data from the Norwegian Mother, Father and Child Cohort Study used in this study are managed by the Norwegian Institute of public health and can be made available to researchers, provided approval from the Regional Committees for Medical and Health Research Ethics (REC), compliance with the EU General Data Protection Regulation (GDPR) and approval from the data owners. The consent given by the participants does not open for storage of data on an individual level in repositories or journals. Researchers who want access to data sets for replication should apply through helsedata.no. Access to data sets requires approval from The Regional Committee for Medical and Health Research Ethics in Norway and an agreement with MoBa.

The UK Biobank phenotype and genetic data described here are publicly available to registered researchers through the UKB data access protocol. Information about registration for access to the data is available at: https://www.ukbiobank.ac.uk/enable-your-research/apply-for-access. This work was conducted using UK Biobank data, application number 9905.

All bona fide researchers can apply to use ALSPAC data for health-related research that is in the public interest. Information regarding the ALSPAC cohort and data access is available at https://www.bristol.ac.uk/alspac/researchers/our-data/.

Research involving human participants, their data, or biological material

Policy information about studies with human participants or human data. See also policy information about sex, gender (identity/presentation), and sexual orientation and race, ethnicity and racism.

Reporting on sex and gender	Throughout the manuscript, the term sex is used to describe the biological attribute. Sex was genetically determined and cross-checked with reported sex. Analyses were performed jointly for males and females.
Reporting on race, ethnicity, or other socially relevant groupings	Only data from individuals of European ancestry were included in the study.
Population characteristics	Our primary analyses include data from the MoBa cohort, UK Biobank (https://doi.org/10.1038/s41586-018-0579-z) and a European meta-analysis on age at menarche (https://doi.org/10.1038/s41588-024-01798-4). We refer to Supplementary Data 1 and the beforementioned cohort descriptions for detailed population characteristics.
Recruitment	MoBa: Pregnant women attending routine ultrasound examination between October 1999 and July 2009 were invited to participate in the study. Participants were recruited from 50 of Norway's 52 hospitals with maternity units, and the participation rate was 41%.<br><br>UK Biobank: Residents in the UK from 40-69 years living within 25 miles from one of 22 assessment centres in 2006-2010 were invited to participate in the study.<br><br>Meta-analysis of age at menarche: This meta-analysis included data from numerous sources with varying recruitment procedures.<br><br>ALSPAC: Pregnant women resident in Avon, UK with expected dates of delivery between 1st of April 1991 and 31st of December 1992 were invited to participate in the study.
Ethics oversight	The administrative board of the Norwegian Mother, Father and Child Cohort Study led by the Norwegian Institute of Public Health approved the study protocol. The establishment of MoBa and initial data collection was based on a license from the Norwegian Data Protection Agency and approval from The Regional Committee for Medical Research Ethics. The MoBa cohort is currently regulated by the Norwegian Health Registry Act. The study was approved by The Regional Committee for Medical Research Ethics (#2012/67).<br>UK Biobank: Informed consent was obtained from all study participants. The study has approval from the North West Multicentre Research Ethics Committee as a Research Tissue Bank.<br>ALSPAC: Ethical approval for the study was obtained from the ALSPAC Ethics and Law Committee and the Local Research Ethics Committees. Informed consent for the use of data collected via questionnaires and clinics was obtained from participants following the recommendations of the ALSPAC Ethics and Law Committee at the time. Consent for biological samples has been collected in accordance with the Human Tissue Act (2004).

Note that full information on the approval of the study protocol must also be provided in the manuscript.

Field-specific reporting

Please select the one below that is the best fit for your research. If you are not sure, read the appropriate sections before making your selection.

☒ Life sciences ☐ Behavioural & social sciences ☐ Ecological, evolutionary & environmental sciences

For a reference copy of the document with all sections, see nature.com/documents/nr-reporting-summary-flat.pdf

Life sciences study design

All studies must disclose on these points even when the disclosure is negative.

Sample size	Sample size for each analysis was determined by the number of participants with available data and who did not meet the exclusion criteria described below. Sample size of the genomic SEM common factor GWAS was estimated as described in the Methods section.

Data exclusions	Participants who did not meet genetic or phenotypic quality control, who were of non-European ancestry or had missing data were excluded from the study.
Replication	No exact replication of the study was done. However, we have made several measures to ensure the validity of our results: gSEM showed high to moderate genetic correlation and high directional concordance with childhood and adult BMI; gSEM-PGS outperforms similar PGSs when predicting anthropometric traits in the independent ALSPAC cohort; and trajectory-specific PGSs observed confirmatory patterns of association with BMI in the independent ALSPAC cohort.
Randomization	Not relevant to the study as participants were not randomly assigned to groups.
Blinding	Not relevant to the study as it does not involve procedures that require masking of participants or researchers.

Reporting for specific materials, systems and methods

We require information from authors about some types of materials, experimental systems and methods used in many studies. Here, indicate whether each material, system or method listed is relevant to your study. If you are not sure if a list item applies to your research, read the appropriate section before selecting a response.

Materials & experimental systems

n/a	Involved in the study
☒	☐ Antibodies
☒	☐ Eukaryotic cell lines
☒	☐ Palaeontology and archaeology
☒	☐ Animals and other organisms
☒	☐ Clinical data
☒	☐ Dual use research of concern
☒	☐ Plants

Methods

n/a	Involved in the study
☒	☐ ChIP-seq
☒	☐ Flow cytometry
☒	☐ MRI-based neuroimaging

Plants

Seed stocks	*Report on the source of all seed stocks or other plant material used. If applicable, state the seed stock centre and catalogue number. If plant specimens were collected from the field, describe the collection location, date and sampling procedures.*
Novel plant genotypes	*Describe the methods by which all novel plant genotypes were produced. This includes those generated by transgenic approaches, gene editing, chemical/radiation-based mutagenesis and hybridization. For transgenic lines, describe the transformation method, the number of independent lines analyzed and the generation upon which experiments were performed. For gene-edited lines, describe the editor used, the endogenous sequence targeted for editing, the targeting guide RNA sequence (if applicable) and how the editor was applied.*
Authentication	*Describe any authentication procedures for each seed stock used or novel genotype generated. Describe any experiments used to assess the effect of a mutation and, where applicable, how potential secondary effects (e.g. second site T-DNA insertions, mosiacism, off-target gene editing) were examined.*
