# Supplementary material for: Genome-wide mapping of common and rare variant effects on adiposity across childhood
Source: Nat Genet. 2026 Sep 25;58(11):2794–803. doi: 10.1038/s41588-026-02772-y (PMC13754613; doi:10.1038/s41588-026-02772-y)
Supplement: Supplementary file 3 [file 41588_2026_2772_MOESM3_ESM.pdf]

nature portfolio

Peer Review File

Genome-wide mapping of common and rare variant effects on adiposity across childhood

Corresponding Author: Professor Stefan Johansson

This file contains all editorial decision letters in order by version, followed by all author rebuttals in order by version.

Version 0:

Decision Letter:

1st July 2025

Dear Dr. Johansson,

Your Article "The role of common and rare genetic variation on adiposity across childhood" has been seen by three referees. You will see from their comments below that, while they find your work of interest, they have raised several relevant points. We are interested in the possibility of publishing your study in Nature Genetics, but we would like to consider your response to these points in the form of a revised manuscript before we make a final decision on publication.

To guide the scope of the revisions, the editors discuss the referee reports in detail within the team, including with the chief editor, with a view to identifying key priorities that should be addressed in revision, and sometimes overruling referee requests that are deemed beyond the scope of the current study. In this case, we ask that you address all technical points related to the association analyses and their interpretation, extending the analyses where feasible, providing additional context where needed, and qualifying the conclusions accordingly. We hope you will find this high-level guidance useful when revising your study. Please do not hesitate to get in touch if you would like to discuss these issues further.

We therefore invite you to revise your manuscript taking into account all reviewer and editor comments. Please highlight all changes in the manuscript text file. At this stage, we will need you to upload a copy of the manuscript in MS Word .docx or similar editable format.

We are committed to providing a fair and constructive peer-review process. Do not hesitate to contact us if there are specific requests from the reviewers that you believe are technically impossible or unlikely to yield a meaningful outcome.

When revising your manuscript:

*1) Include a “Response to referees” document detailing, point-by-point, how you addressed each referee comment. If no action was taken to address a point, you must provide a compelling argument. This response will be sent back to the referees along with the revised manuscript.

*2) If you have not done so already, please begin to revise your manuscript so that it conforms to our Article format instructions, available
here.
Refer also to any guidelines provided in this letter.

*3) Include a revised version of any required Reporting Summary: https://www.nature.com/documents/nr-reporting-summary.pdf
It will be available to referees (and, potentially, statisticians) to aid in their evaluation if the manuscript goes back for peer review.
A revised checklist is essential for re-review of the paper.

Please be aware of our guidelines on digital image standards.

EXTENDED DATA FIGURES

When re-submitting your manuscript, please ensure that any supplementary figures and tables that are crucial to the manuscript's conclusions are converted into Extended Data figures and tables to increase visibility of these data. Extended Data figures and tables are online-only (present in the online PDF and full-text HTML versions of the paper), peer-reviewed display items that provide essential background to the article but are not included in the main article due to space constraints. A maximum of ten Extended Data display items (figures and tables) is permitted.

Please use the link below to submit your revised manuscript and related files:

Link Redacted

Note: This URL links to your confidential home page and associated information about manuscripts you may have submitted, or that you are reviewing for us. If you wish to forward this email to co-authors, please delete the link to your homepage.

We hope to receive your revised manuscript within 8-12 weeks. If you cannot send it within this time, please let us know.

Please do not hesitate to contact me if you have any questions or would like to discuss these revisions further.

Nature Genetics is committed to improving transparency in authorship. As part of our efforts in this direction, we are now requesting that all authors identified as 'corresponding author' on published papers create and link their Open Researcher and Contributor Identifier (ORCID) with their account on the Manuscript Tracking System (MTS), prior to acceptance. ORCID helps the scientific community achieve unambiguous attribution of all scholarly contributions. You can create and link your ORCID from the home page of the MTS by clicking on 'Modify my Springer Nature account'. For more information, please visit www.springernature.com/orcid.

We look forward to seeing the revised manuscript and thank you for the opportunity to review your work.

Sincerely,
Kyle

Kyle Vogan, PhD
Senior Editor
Nature Genetics
https://orcid.org/0000-0001-9565-9665

Referee expertise:

Referee #1: Genetics, obesity, metabolic disorders

Referee #2: Genetics, obesity, metabolic disorders

Referee #3: Genetics, obesity, metabolic disorders

Reviewers' Comments:

Reviewer #1 (Remarks to the Author):

The manuscript presents a series of analyses aimed at understanding the genetic drivers of childhood obesity and compare those genes and mechanisms with the more robust literature on the genetics of adult obesity/BMI. Among the major challenges in this area is the dramatically smaller availability of relevant study samples with data on body weight at early life time points. They first present updated results from one of the few longitudinal birth cohorts, MoBa, with body weight/age-scaled BMI data, comparing both heritability and discovery yield across the time periods (from a few weeks up to 8 years of age).

Acknowledging the lack of data in this space, the authors then attempt to pull in summary data from other less direct sources using genomic Structural Equation Modeling (gSEM), (a) recall data on childhood obesity at age 10 in UKB, and (b) age at menarche data due to the strong correlation between childhood obesity and early onset of menarche. Together these data dramatically increase the amount of available data for assessing the genetic contributions to childhood obesity.

Following discovery analysis across the three data sets, the authors clustered the 624 independent variants with respect to childhood BMI trajectory, identifying four main types: early-rise, birth-related, transient, and U-shaped.

Gene prioritization among the associations highlight several major established pathways in obesity, including the leptin-

melanocortin and incretin pathways. Analyses in 25,779 trios in MoBa identified parent-of-origin effects in several loci. Consistent with many of these associations, childhood obesity loci are enriched for expression in hypothalamic cell populations.

Finally, they examined gene-based associations from whole-genome sequence data for the childhood obesity recall data in UKB, identifying six associated genes, again implicating the leptin-melanocortin pathway.

The paper presents a broad and intriguing set of analyses that bring considerably more depth to the understanding of childhood obesity patterns, with interesting contrasts with established adult obesity genetics. Overall, the analyses appear to be well thought out, described, and interpreted. There does not seem to be lot of clarity of the impact of childhood obesity genetic profiles on long-term health outcomes, particularly those independent of canonical life-long obesity pathways.

Comments

1) Of primary concern is the initial discovery p-value threshold of 5E-8. In the MoBa analysis, the authors perform GWAS on 11 timepoints. Beyond that they also perform genome-wide analysis on gSEM, and gene-based tests for SAC10. It is hard to know whether the low levels of concordance between the results presented here and previous genetic assessments of childhood obesity are due to lenient multiple testing thresholds leading to false positives, increased sample sizes, or some combination of the two. Both a more stringent discovery threshold and a better assessment of the relationship between previous discoveries and new results would be useful.

2) While SAC10 is likely a rough phenotype for adiposity, it is clearly relevant. The age at menarche approach, while clearly relevant given the established relationship with obesity, is harder to evaluate. I think it was vitally important to remove the loci loading uniquely to AAM and not the other two phenotypes. There isn't a lot of information/data to evaluate the validity/value of the overall gSEM approach or the impact of removing "AAM-specific" genes/variants.

3) The vast majority of the gene-function discussion highlights relationships with known pathways in both childhood and adult obesity. While these relationships are reassuring that the results are of high quality, they don't reveal much in the way of new biological information, despite high proportion of variants and genes that differ from adult obesity genetics. A deeper dive into novel genes and pathways could be more informative in contrasting the factors at play. Similarly, contrasting the genetic drivers of each of the obesity trajectory clusters to give them better relevance would be of interest.

4) MR analysis attempts to assess the causal relationship between the childhood trajectory clusters and diseases with known relation to adult BMI, such as diabetes, with all but the "transient" cluster showing association. Given the number of unique associations for early childhood trajectories, it is a little surprising that the long-term cardiometabolic risk is essentially fully attributable the genetic aspects that are consistent throughout the lifespan. Given the number of obesity-associated disease outcomes, this exploration was fairly limited in scope, though. Would be interesting to see if that pattern is consistent across a broad array of obesity-related outcomes. Would also be interesting if there were any components of childhood obesity/BMI, be that specific genes, trajectories, or pathways, that still contribute to disease outcomes independent of, or after adjusting for adult obesity.

5) What does it mean that childhood and adult obesity analyses implicate many of the same genes, but often through different variants? Is it lack of specificity of causal variants due to power? Differential regulation over the developmental and adult time periods? Activity in different tissues/cell types? Was this detectable in the Hypomap analysis?

6) Related to the comments on the MR analyses, it is hard to understand the long term relevance/meaning of the childhood trajectories for long term outcomes.

Reviewer #2 (Remarks to the Author):

This paper presents a genome-wide association study (GWAS) of childhood adiposity traits. The meta-analysis includes data from 62,000 children from the MoBa cohort, which offers unique longitudinal data on BMI during early childhood. To expand the scope, the authors applied a genomic structural equation modeling (gSEM) approach, integrating zBMI data at age 8 from MoBa, comparative body size at age 10 from the UK Biobank, and a GWAS meta-analysis on age at menarche. This yielded a total effective sample size of nearly 600,000 individuals.

The study identified 624 genetic loci associated with childhood adiposity, one-third of which did not show directionally concordant associations with adult BMI. These loci were grouped into four distinct trajectories: “early risers,” “late risers,” “transient,” and “U-shaped” patterns of influence across early development. The authors propose that body weight regulation in childhood may be governed by distinct neurocircuitry compared to adulthood, and may provide a more sensitive window for detecting variation key in body weight regulation.

While the study reveals new information, it also raises concerns.

General comments

1. Given that a large number of newly identified loci are found for the very early age categories, it would be helpful if the authors could elaborate more on what BMI represents at these early developmental stages. Specifically, how does early-life

BMI relate to BMI at later ages, and what is its relevance to long-term obesity risk and overall health outcomes? From Figure 1D, it appears that BMI before age 5 shows minimal/modest correlation with adult BMI, and the MR analyses suggest that the so-called "transient loci" do not significantly impact future health. This raises the question of the broader significance of studying BMI at such early ages. Therefore, the authors should provide more context and interpretation to help readers understand the implications of early-life BMI findings.

2. Related to the first comment, it would be valuable to clarify whether BMI at these early stages truly reflects adiposity. Given that BMI was developed for use in adults — who have reached their final height and whose body proportions remain stable — it is not immediately clear whether the same assumptions hold in infants and young children, whose body composition and proportions change rapidly with age. Can loci identified in early infancy genuinely be described as “adiposity-related loci,” or are they more accurately described as influencing variation in BMI, which may not directly equate to adiposity at this age? A discussion on this distinction would help readers better understand the biological relevance of the findings.

3. A key strength of this study is the MoBa cohort, which offers a unique longitudinal design with repeated measurements very early in life — something that is relatively rare in childhood cohorts. This aspect adds significant value to the investigation of early-life growth and adiposity trajectories. However, I am less convinced of the added value of increasing statistical power for adiposity traits later in childhood by incorporating data from recalled phenotypes such as “comparative body size at age 10” (categorized as “plumper,” “average,” or “thinner”) and age at menarche. Furthermore, while loci associated with age at menarche often overlap with adiposity loci, it would be helpful for the authors to discuss how the inclusion of these sex-specific variables might influence the interpretation of sex effects in their analyses.

4. Adiposity rebound and adiposity peak (age and BMI) are well-established metrics that provide valuable insights into early childhood growth patterns and, at least adiposity rebound is a known predictor of future obesity risk. Surprisingly, these metrics are not mentioned or discussed in the manuscript. It would strengthen the study to include a discussion on how the current findings relate to these established growth markers. For example, do the identified loci show association with any of these growth metrics?

5. While the study identifies many new loci and includes post-GWAS analyses, including comparisons with adult data, single cell data, CSTG, and more, it remains unclear what the key novel insights are in terms of understanding or predicting future obesity risk and what the key biological factors are that distinguish the regulation of childhood and adulthood body weight. The manuscript would benefit from a clearer articulation of how these findings advance our knowledge beyond what is already known from previous studies. Clarifying the translational significance would help underscore the broader impact of the study.

6. Given the well-documented differences in growth patterns and changes in fat and lean mass between boys and girls, it would be important to assess whether any sex-specific genetic effects were observed.

Specific comments

ABSTRACT

L48: Split sample size across measured and recalled data, allowing readers to better assess quality of data.

L62: Add significance level for the “stronger adiposity associations” in adults.

RESULTS

L101: How many boys and girls?

L114: Were there any signals reported in the previous paper that were not identified here? It seems that the previous paper reported 46 loci.

L156-164: I have read through the methods, but it remains unclear to me how effect sizes of these difference GWAS (SAC10, AAM) can be compared as they do not seem to be on the same scale.

Figure 3A: The effect sizes of the transient loci are--between 6 weeks and 5 years--about 2 to 5 times larger than that of the other clusters. How can this be explained?

Figure 3B: Isn’t it surprising that the variant in MC4R is a “late-rising” one, as rare variants in MC4R are known to cause early-onset obesity?

Figure 3B: Is it correct that rs77226405 an early rise locus? It does not seem to differ from the other MC4R locus, which is considered a late rising one.

L207: Does that variant-to-gene mapping reveal new insights not seen for loci identified in adults?

L282: Clusters are not super clear. My impression was that the GLP1R locus was transient, where it is here described as an early-rising one.

L327: How do the observations in hypothalamic neurons for childhood-associated loci provide new insights beyond what has already been reported in adult data?

L340: CALCR is only distantly part of the leptin-melanocortin pathway; it seems unfair to consider it at the same levels as LEPR and POMS.

L344: GRP is not an incretin or incretin-related.

DISCUSSION

L431-444: The inclusion of the AAM data to boost the power to identify more adiposity loci relies on the assumption that adiposity affects AAM. Can it be tested whether these variants indeed affect BMI in the first place?

Reviewer #3 (Remarks to the Author):

This is a very interesting paper that sets a new benchmark in GWAS approaches to complex traits. It is the best example I can think of that shows how the genetic architecture of the “same” trait can be different between two life-stages. The fact that there are so many fascinating differences between the causes and consequences of excess weight in childhood – including protective effects on conditions such as breast cancer – and excess weight in adulthood, adds to the importance and interest. I have a few observations and comments, none of which are major concerns but may help the reader if addressed:

1. For readers familiar with GWAS and genetics of obesity and puberty timing, the genomic SEM approach will make some sense. It seems to me pretty convincing that the previous AAM GWASs were in effect GWAS for two traits – “pure” puberty and adiposity that leads to variation in pubertal timing. However, to some readers this may take some extra brain gymnastics and it may be worth spelling out even more. For example, looking at Supp Figure 2, I think it is clear that the homogenous gSEM variants are more specific for adiposity than AAM, but plots of AAM vs MOBA8 and AAM vs SAC10 could show a more striking separation? Similarly, it may help the reader if the authors could substantiate the statement in the results (from lines 147), about half the AAM signals acting through childhood adiposity. How does this predefined list of 50% match up to those that were GWAS significant in the genomic SEM model bit did not showing evidence of heterogeneity in the QSNP test? An extra supp table may be useful?

2. Trajectory specific PRS and methods lines 662 - 673 – to what extent are these the result of dividing up continuous variation in how an overall PGS affects growth, and genuine heterogeneity? I do not see the stats in STs 10 and 11 that show the elbow effects or other metrics that provide evidence of genuine differences. I have no doubt about the heterogenous effects between birth and childhood and between childhood and adulthood but forcing all variants into one of 4 groups may introduce some arbitrariness. The fact that the clusters are similar to an earlier analysis in MOBA and are “homogeneous” after splitting up does not provide much reassurance that these 4 clusters are really representing something separate as, respectively, the training and test data overlap and once a continuous variable is split up into 4 groups the effects will look homogenous and similar in other data. The potential arbitrariness of the assignment into clusters is shown in Figure 3 where the two PCSK1 signals are assigned to the same cluster but appear to swap over effect sizes between early and later childhood. In contrast, the 2 MC4R signals are assigned to different clusters but look quite similar in pattern with potential differences in when they appear explained by one having a weaker effect size?

3. There appear to be signals in same gene/locus doing different things (although see point 2 maybe this is chance?). If the signals are non-coding, it is easier to imagine how that could happen – different regulatory elements being affected at different times of life. For coding variants, this is harder to imagine unless obvious differences in the location of the variants in relation to different transcripts expressed at different times of life. Or maybe just an interesting mystery and worth mentioning in the limitations?

Minor points

1. Abstract. Although the word “upto” technically covers it, a large proportion of the power of the GWAS discovery component to the study comes from the cross-sectional UK biobank age 10 and AAM data. So “longitudinal study of upto 599,000 people” is a little misleading. Maybe “including the largest genetic study of longitudinal...” - however see next point, there is a recently published study the authors should place their study in this new context.

2. Introduction line 79. Given the Richardson et al SAC and AAM GWAS data are “childhood adiposity related” traits critical to the study, and there is a cross-sectional BMI in childhood GWAS of 84k people, very recently published (Trans-ancestry genome-wide association study of childhood body mass index identifies novel loci and age-specific effects - PubMed) maybe reword "However, previous GWAS of directly measured BMI in childhood have been limited to a GWAS of single time point measures..."

3. Introduction. Probably worth a more explicit sentence on the Richardson et al study that did show very nicely the differences between childhood and adult BMI on major outcomes.

4. Results line 116. Of the 152 signals, how many overlap with GIANT 2018 data?

5. Methods line 491. There were some measures reported by parents rather than measured? This sentence does not flow well from the preceding one.

6. Line 562 an apostrophe missing from SNPs (sorry)

7. Line 423 comparing WES based signals and the striking difference between the 8 discovered in adults and 6 in children that have little overlap. It could be more helpful to reader to talk about differences in effect sizes than whether or not discovered at a threshold. I think many of the childhood ones will have effects in adults, but just weaker?

8. Line 553 A Heywood case? worth a few more words to explain that

9. Discussion opening line. The signals and effective sample size are heavily driven by the age at 10 size in UK Biobank (looking at SF2) and the AAM GWAS. Given both of these GWAS are previously published (the first in the Richardson study), it is probably worth toning down the "by far the largest ever" wording.

10. Discussion paragraph starting on line 406. I am not sure the second sentence follows from the first? Perhaps some more nuanced wording needed?

Version 1:

Decision Letter:

23rd October 2025

Dear Dr. Johansson,

Your revised Article "The role of common and rare genetic variation on adiposity across childhood" has been seen by the original referees. You will see from their comments below that, while they find the study improved, they have raised a few ongoing concerns. We remain interested in the possibility of publishing your study in Nature Genetics, but we would like to consider your response to these remaining concerns in the form of a further revision before we make a final decision on publication.

We therefore invite you to further revise your manuscript taking into account these comments. Please highlight all changes in the manuscript text file. At this stage, we will need you to upload a copy of the manuscript in MS Word .docx or similar editable format.

We are committed to providing a fair and constructive peer-review process. Do not hesitate to contact us if there are specific requests from the reviewers that you believe are technically impossible or unlikely to yield a meaningful outcome.

When revising your manuscript:

*1) Include a "Response to referees" document detailing, point-by-point, how you addressed each referee comment. If no action was taken to address a point, you must provide a compelling argument. This response will be sent back to the referees along with the revised manuscript.

*2) If you have not done so already, please begin to revise your manuscript so that it conforms to our Article format instructions, available
here.
Refer also to any guidelines provided in this letter.

*3) Include a revised version of your Reporting Summary: https://www.nature.com/documents/nr-reporting-summary.pdf
It will be available to referees (and, potentially, statisticians) to aid in their evaluation if the manuscript goes back for peer review.
A revised checklist is essential for re-review of the paper.

Please be aware of our guidelines on digital image standards.

EXTENDED DATA FIGURES

When re-submitting your manuscript, please ensure that any supplementary figures and tables that are crucial to the manuscript's conclusions are converted into Extended Data figures and tables to increase visibility of these data. Extended Data figures and tables are online-only (present in the online PDF and full-text HTML versions of the paper), peer-reviewed display items that provide essential background to the article but are not included in the main article due to space constraints. A maximum of ten Extended Data display items (figures and tables) is permitted.

Please use the link below to submit your revised manuscript and related files:

Link Redacted

Note: This URL links to your confidential home page and associated information about manuscripts you may have submitted, or that you are reviewing for us. If you wish to forward this email to co-authors, please delete the link to your homepage.

We again hope to receive your revised manuscript within 8-12 weeks. If you cannot send it within this time, please let us know.

Please do not hesitate to contact me if you have any questions or would like to discuss these revisions further.

Nature Genetics is committed to improving transparency in authorship. As part of our efforts in this direction, we are now requesting that all authors identified as 'corresponding author' on published papers create and link their Open Researcher and Contributor Identifier (ORCID) with their account on the Manuscript Tracking System (MTS), prior to acceptance. ORCID helps the scientific community achieve unambiguous attribution of all scholarly contributions. You can create and link your ORCID from the home page of the MTS by clicking on 'Modify my Springer Nature account'. For more information, please visit www.springernature.com/orcid.

We look forward to seeing the revised manuscript and thank you for the opportunity to review your work.

Sincerely,
Kyle

Kyle Vogan, PhD
Senior Editor
Nature Genetics
https://orcid.org/0000-0001-9565-9665

Referee expertise:

Referee #1: Genetics, obesity, metabolic disorders

Referee #2: Genetics, obesity, metabolic disorders

Referee #3: Genetics, obesity, metabolic disorders

Reviewers' Comments:

Reviewer #1 (Remarks to the Author):

The authors have made a concerted effort to address the many concerns addressed by all the reviewers, and as such have done much to improve the quality of the manuscript. I appreciate the additional clarity the authors brought to the work with respect to replication of previous MoBa results and also from unrelated data. These comparisons are much more convincing now. The added information with respect to the gSEM in both the main and supplementary text are also helpful in convincing the reader that this approach was meaningful and appropriate. Despite the considerable added information, I do think two main areas are preventing the paper from reaching its full potential.

A few comments:

1) Multiple reviewers noted the valuable missing piece of understanding the long-term impact or significance of childhood obesity, particularly as it relates to the genetic contribution. Exploring this more broadly than the extremely limited set of cardiometabolic traits currently mentioned would dramatically enhance the value of the paper. While I do appreciate the potential scale of additional work, and I could be convinced there is more value in examining these relationships in an entirely different paper, suggesting they are beyond relevance for the paper is not convincing.

2) It appears, through the review process, the authors feel their key discovery in this work is the apparent differential regulation of core obesity genes/pathways between early life obesity and adult obesity. The authors do make this broad conclusion more clear, but I both feel this "key finding" is under-represented in the text and that the data supporting this conclusion, as opposed to other alternate hypotheses, could be shown more clearly and discussed in more detail. It is mostly presented as conjecture in the discussion. If well supported, I do think this is a particularly interesting and important conclusion from this work, and probably should be mentioned in the abstract as well.

Reviewer #2 (Remarks to the Author):

A few concerns/comments remain:

Response to comment 1:
In their response, the authors focus on weight gain, which was not examined in the current study. Moreover, the cited papers report evidence of weight gain only within the first year of life, with no data or findings presented for the subsequent early years. As such, I still find there to be limited clarity around what cross-sectional BMI at these very early ages actually represents, and whether it can be reliably defined as a measure of adiposity.

The proposed PheWAS analysis could help clarify this, and I would suggest including at least a table to provide further insight. In my view, understanding what early BMI truly reflects is central to the study's aims.

Response to comment 2:
The Olga et al. study does not examine BMI, nor does the Catalano et al. study. Both focus on other anthropometric traits, but not BMI specifically. Similarly, the authors' response does not directly address BMI, instead concentrating on weight and length. To strengthen the argument, it would be helpful to include more appropriate references that specifically quantify the relationship between BMI and adiposity in very early life.

Response to comment for L114:
Are there any insights into the 10 loci (out of 46, or approximately 20%) reported by Helgeland et al. that were not replicated in the current study?

Reviewer #3 (Remarks to the Author):

Many thanks for the clarifications. The metrics on clustering are a very useful addition, but really point to, at best, evidence that signals can be split into two groups, not four (based on the silhouette metrics that relate to the average distance of individual datapoints to all those in the same cluster compared to all those in different clusters). I know it will be a little painful to reformat these results, but it would be a much better representation of the evidence to talk about 2 clusters. By splitting into 4, there is a risk of over interpretation by readers and all those researchers wishing to follow up what is otherwise a fascinating paper.

Version 2:

Decision Letter:

Our ref: NG-A69162R1

10th February 2026

Dear Dr. Johansson,

Your revised manuscript "The role of common and rare genetic variation on adiposity across childhood" (NG-A69162R1) has been seen by the original reviewers. As you will see from their comments below, they find that the paper has improved in revision, and therefore we will be happy in principle to publish it in Nature Genetics as an Article pending final revisions to address their remaining minor points and to comply with our editorial and formatting guidelines.

We are now performing detailed checks on your paper, and we will send you a checklist detailing our editorial and formatting requirements soon. Please do not upload the final materials or make any revisions until you receive this additional information from us.

Thank you again for your interest in Nature Genetics. Please do not hesitate to contact me if you have any questions.

Sincerely,
Kyle

Kyle Vogan, PhD
Senior Editor
Nature Genetics
https://orcid.org/0000-0001-9565-9665

Reviewer #1 (Remarks to the Author):

The authors continue to make a concerted effort to improve their work based on reviewer feedback. Both the shift to a two-cluster model and the further characterization of the two-cluster model with a more comprehensive phewas and MR analysis have made the work much clearer and more compelling.

As with reviewer #3, I find the two-cluster model much more consistent with the data and the results and conclusions easier to understand and interpret.

The expanded phewas and comparison of transient vs persistent associations reveals both a rather expected outcome and one that is potentially less expected. The strong relationship between persistent childhood associations and adult BMI and their lifelong disease risk reflects what might have been expected a priori. Of potential interest is whether these are more likely to be shared genetic signals as opposed to unique or differentially regulated loci. I would assume so.

I also appreciate the deeper analysis of the shared vs unique associations between childhood and adult BMI and the potential for differential regulation. Despite strong pathway overlap with adult BMI, the analysis exemplifies what appears to be stronger power in children for a large number of genes/variants (childhood "unique" genes), while only a small portion appear to be differentially regulated.

While the expanded phewas in some ways diminishes the pure novelty of the work because the majority of associations with future disease risk match well with "persistent" genes discovered by adult BMI/obesity, making the relationship between persistent and transient genes and subsequent long-term risk is critically important and valuable. Of potentially more interest is the relevance of protective signals observed for transient obesity genes/variants. What could account for this effect? Regardless, seeing this interesting contrast between the groups, and understanding the relationship to adult BMI/obesity is valuable and a useful addition to the manuscript.

Overall, the authors have sufficiently resolved my major concerns/questions with the work. Thank you for the attentiveness with which you addressed each concern and push for clarity in the analysis and results.

Reviewer #2 (Remarks to the Author):

The paper has improved substantially and results and message of more informative and clear. I have no further major comments, however, I suggest reviewing the genes listed for consistency; e.g. at P10 LEPR is defined as "transient", whereas at P13 is is defined as persistent.

Reviewer #3 (Remarks to the Author):

Many thanks for the additional work. The two clusters look much more convincing than the four. Bring on the next batch of 80k to sort that out.

Open Access This Peer Review File is licensed under a Creative Commons Attribution 4.0 International License, which permits use, sharing, adaptation, distribution and reproduction in any medium or format, as long as you give appropriate credit to the original author(s) and the source, provide a link to the Creative Commons license, and indicate if changes were made.
In cases where reviewers are anonymous, credit should be given to 'Anonymous Referee' and the source.
The images or other third party material in this Peer Review File are included in the article’s Creative Commons license, unless indicated otherwise in a credit line to the material. If material is not included in the article’s Creative Commons license and your intended use is not permitted by statutory regulation or exceeds the permitted use, you will need to obtain

permission directly from the copyright holder.
To view a copy of this license, visit https://creativecommons.org/licenses/by/4.0/

Kyle Vogan, PhD
Senior Editor
Nature Genetics

Dear Editor,
We thank the editor and reviewers for the positive assessment of our work and for giving us the opportunity to submit a revised manuscript.

Please, find below our point-by-point response to all of the comments and questions raised by the reviewers. We have included a number of new analyses and illustrations and revised and clarified the text. Changes in the manuscript are clearly marked and highlighted in red.

We are grateful to all reviewers for their comments and suggestions, and believe that the manuscript has improved considerably due to the review process. We hope that the manuscript can now be considered for publication in Nature Genetics, and we look forward to hearing from you.

On behalf of all authors,

Stefan Johansson PhD
Professor and Head of the Diabetes Research Groups
Department for Clinical Science, University of Bergen
and
Clinical Laboratory Geneticist at
The Department for Medical Genetics
Haukeland University Hospital
stefan.johansson@uib.no

“Response to referees” letter:

Reviewer #1 (Remarks to the Author):

The manuscript presents a series of analyses aimed at understanding the genetic drivers of childhood obesity and compare those genes and mechanisms with the more robust literature on the genetics of adult obesity/BMI. Among the major challenges in this area is the dramatically smaller availability of relevant study samples with data on body weight at early life

time points. They first present updated results from one of the few longitudinal birth cohorts, MoBa, with body weight/age-scaled BMI data, comparing both heritability and discovery yield across the time periods (from a few weeks up to 8 years of age).

Acknowledging the lack of data in this space, the authors then attempt to pull in summary data from other less direct sources using genomic Structural Equation Modeling (gSEM), (a) recall data on childhood obesity at age 10 in UKB, and (b) age at menarche data due to the strong correlation between childhood obesity and early onset of menarche. Together these data dramatically increase the amount of available data for assessing the genetic contributions to childhood obesity.

Following discovery analysis across the three data sets, the authors clustered the 624 independent variants with respect to childhood BMI trajectory, identifying four main types: early-rise, birth-related, transient, and U-shaped.

Gene prioritization among the associations highlight several major established pathways in obesity, including the leptin-melanocortin and incretin pathways. Analyses in 25,779 trios in MoBa identified parent-of-origin effects in several loci. Consistent with many of these associations, childhood obesity loci are enriched for expression in hypothalamic cell populations.

Finally, they examined gene-based associations from whole-genome sequence data for the childhood obesity recall data in UKB, identifying six associated genes, again implicating the leptin-melanocortin pathway.

The paper presents a broad and intriguing set of analyses that bring considerably more depth to the understanding of childhood obesity patterns, with interesting contrasts with established adult obesity genetics. Overall, the analyses appear to be well thought out, described, and interpreted. There does not seem to be lot of clarity of the impact of childhood obesity genetic profiles on long-term health outcomes, particularly those independent of canonical life-long obesity pathways.

Comments

1) Of primary concern is the initial discovery p-value threshold of 5E-8. In the MoBa analysis, the authors perform GWAS on 11 timepoints. Beyond that they also perform genome-wide analysis on gSEM, and gene-based tests for SAC10. It is hard to know whether the low levels of concordance between the results presented here and previous genetic assessments of childhood obesity are due to lenient multiple testing thresholds leading to false positives, increased sample sizes, or some combination of the two. Both a more stringent discovery threshold and a better assessment of the relationship between previous discoveries and new results would be useful.

Response: We chose to report all loci exceeding the $P<5\times10^{-8}$ threshold for all the 11 childhood timepoints in MoBa and the gSEM model as is standard in the field and makes the results easily comparable across studies. We have now also added in the main text that 106 of the 152 MoBa lead SNPs pass a more stringent $P<1.04\times10^{-8}$, calculated

accounting for multiple timepoints and the correlations in BMI across timepoints in MoBa (added to Results Line 128-129 and Methods).

We apologise for the incorrect impression that there was low concordance between the current and previous genetic findings for childhood obesity in MoBa. On the contrary, we find high concordance with those findings, which supports the standard $P<5\times10^{-8}$ threshold used in both studies. 36 of the 46 SNPs reported in Helgeland et al. (2022) are relevant for replication here (excluding 9 SNPs for birth weight, and 1 X-chromosome SNP). Of these 36 SNPs, 32 (the same variant or a proxy $R^2>0.5$) show genome-wide significant ($P<5\times10^{-8}$) association with a childhood adiposity trait here. Of the other 4 SNPs in Helgeland et al. (2022), 3 show directionally consistent sub-genome-wide significant associations ($P<6\times10^{-5}$) with childhood adiposity here: *PLCE1* (Helgeland SNP: rs1830890), *LCORL* (rs2610989), *KLF14* (rs12672489). Only, *RIN2* (rs148252705) shows a more modest but directionally consistent association in the current data ($P=4\times10^{-3}$).

Similarly, we also find high concordance with the reported findings of Vogelzang et al (EGG consortium, 2020) who also used the standard $P<5\times10^{-8}$ threshold. All 24 of their reported signals with testable data in our sample were represented by a signal in our current data (sTable 9).

To meet these concerns we have 1) rephrased the results section to increase clarity, 2) added a comment on the use of this GWAS threshold and the possibility of false positive findings, and 3) described the comparison to the Helgeland 2022 and Vogelzang 2019 studies (Discussion Page 21, para 2, rows 525-532).

2) While SAC10 is likely a rough phenotype for adiposity, it is clearly relevant. The age at menarche approach, while clearly relevant given the established relationship with obesity, is harder to evaluate. I think it was vitally important to remove the loci loading uniquely to AAM and not the other two phenotypes. There isn't a lot of information/data to evaluate the validity/value of the overall gSEM approach or the impact of removing "AAM-specific" genes/variants.

Response: We have now added more details on the development and validation of the gSEM approach in the supplementary file that now comprises 12 pages with 7 figures and two tables (pages 10-21). We have here also quantified the benefit of removing the 117 heterogenous variants (Q-SNPs) with disproportionately large impact on AAM compared to adiposity. For the chosen model 2, after excluding the Q-SNPs the effect direction concordance with the independent child BMI EGG-discovery sample increases from 85% to 91%, and effect direction concordance with adult BMI (in UK Biobank) increases from 88% to 93%. These data are added to the Supplementary Information (Page 19-20).

3) The vast majority of the gene-function discussion highlights relationships with known pathways in both childhood and adult obesity. While these relationships are reassuring that the results are of high quality, they don't reveal much in the way of new biological information, despite high proportion of variants and genes that differ from adult obesity genetics. A deeper dive into novel genes and pathways could be more informative in contrasting the factors at play. Similarly, contrasting the genetic drivers of each of the obesity trajectory clusters to give them better relevance would be of interest.

Response: Exploration of the specific neuronal cells in the hypothalamus provides new biology linked to childhood obesity. We identified specific cell types in the hypothalamus arcuate nucleus and mammillary bodies that appear to be enriched for

genes with child-specific effects on adiposity. Furthermore, both of these groups of cells show high expression of leptin-melanocortin pathway genes.

We find no obvious mechanistic distinction between those leptin-melanocortin pathway genes that show Transient effects on adiposity (*BSX*, *LEPR* and *PCSK1*) and those that show Early rise/Late rise or both patterns (*BDNF*, *GNAS*, *LEP*, *MC4R*, *NTRK2*, *NPY*, *PCSK2* and *POMC*). Our rare variant WGS analysis further indicates that the identified 4 leptin-melanocortin genes (*ADCY3, MC4R, MRAP2* and *POMC*) have stronger effects in childhood than in adults. We therefore conjecture that some child or adult adiposity GWAS signals may be explained by 'life-course dependent regulation', i.e. life-course dependent signals have differential actions on cell-specific regulators of gene expression.

We have added further comment on the likely mechanism of child-specific adiposity signals to the Discussion (Page 19, Para 1-2).

4) MR analysis attempts to assess the causal relationship between the childhood trajectory clusters and diseases with known relation to adult BMI, such as diabetes, with all but the "transient" cluster showing association. Given the number of unique associations for early childhood trajectories, it is a little surprising that the long-term cardiometabolic risk is essentially fully attributable the genetic aspects that are consistent throughout the lifespan. Given the number of obesity-associated disease outcomes, this exploration was fairly limited in scope, though. Would be interesting to see if that pattern is consistent across a broad array of obesity-related outcomes. Would also be interesting if there were any components of childhood obesity/BMI, be that specific genes, trajectories, or pathways, that still contribute to disease outcomes independent of, or after adjusting for adult obesity.

Response: Previous papers (f ex Richardson 2020) have also addressed this question, reporting that child adiposity may have independent effects on breast cancer and type 1 diabetes, but not on cardiometabolic traits after accounting for adulthood adiposity, which aligns with our findings. We agree that a broader approach would be interesting, such as PheWAS and also exploration of instrumental variables for specific pathways. However, this would require much more comprehensive analyses that we feel would fit better in subsequent projects and papers.

5) What does it mean that childhood and adult obesity analyses implicate many of the same genes, but often through different variants? Is it lack of specificity of causal variants due to power? Differential regulation over the developmental and adult time periods? Activity in different tissues/cell types? Was this detectable in the Hypomap analysis?

Response: We conjecture that in these cases differences between child or adult adiposity GWAS signals may be explained by their differential actions on cell-specific regulators of gene expression, rather than the presence of child or adult adiposity genes and pathways. Unfortunately, the limited number of samples available in Hypomap limited the ability to examine changes in expression with age, or to link expression to specific variants.

6) Related to the comments on the MR analyses, it is hard to understand the long term relevance/meaning of the childhood trajectories for long term outcomes.

Response: We agree that it is not clear yet how these trajectories translate into long-term health and that this should be the focus on further research. However, the lack of independent MR effects in our restricted set of MR-analyses does not mean that these timepoints are unimportant for understanding fundamental biology beyond childhood as highlighted by the significant associations with known obesity drug targets that are stronger in childhood than in adults. In response to Reviewer 2 comment #1 below, we have expanded the rationale for the study of early life adiposity (Introduction Page 4, Para 2), explaining links between early growth and later adiposity, and also growing interest in efforts to prevent overweight and obesity starting from very early in life. Hence, the MR findings of later metabolic consequences of early adiposity driven by later adiposity are relevant and consistent with other types of evidence.

Reviewer #2 (Remarks to the Author):

This paper presents a genome-wide association study (GWAS) of childhood adiposity traits. The meta-analysis includes data from 62,000 children from the MoBa cohort, which offers unique longitudinal data on BMI during early childhood. To expand the scope, the authors applied a genomic structural equation modeling (gSEM) approach, integrating zBMI data at age 8 from MoBa, comparative body size at age 10 from the UK Biobank, and a GWAS meta-analysis on age at menarche. This yielded a total effective sample size of nearly 600,000 individuals.

The study identified 624 genetic loci associated with childhood adiposity, one-third of which did not show directionally concordant associations with adult BMI. These loci were grouped into four distinct trajectories: "early risers," "late risers," "transient," and "U-shaped" patterns of influence across early development. The authors propose that body weight regulation in childhood may be governed by distinct neurocircuitry compared to adulthood, and may provide a more sensitive window for detecting variation key in body weight regulation.

While the study reveals new information, it also raises concerns.

General comments

1. Given that a large number of newly identified loci are found for the very early age categories, it would be helpful if the authors could elaborate more on what BMI represents at these early developmental stages. Specifically, how does early-life BMI relate to BMI at later ages, and what is its relevance to long-term obesity risk and overall health outcomes? From Figure 1D, it appears that BMI before age 5 shows minimal/modest correlation with adult BMI, and the MR analyses suggest that the so-called "transient loci" do not significantly impact future health. This raises the question of the broader significance of studying BMI at such early ages. Therefore, the authors should provide more context and interpretation to help readers understand the implications of early-life BMI findings.

Response: Interest in the regulation of early adiposity is motivated by observed links between early growth and later adiposity and efforts to prevent overweight and obesity starting from early in life, e.g. by targeting infant feeding (Ekelund *et al.*, 2006; Savage *et al.*, 2016)). Such preventive efforts are typically evaluated by their persisting effects on adiposity (Paul *et al.*, 2018, 2025).

In this regard, it is relevant that effects of our Transient adiposity signals peak at age 6 to 8 months, when genetic correlations with adult BMI are modest but detectable (0.18-0.19), and 43% of these Transient signals show directionally-concordant effects on adult BMI. We agree that it is less clear what is the relevance to early and later health of the 57% of Transient signals with no evidence of directionally-concordant associations in adults (based on P>=0.05). This should be a topic for subsequent comprehensive assessment e.g. by PheWAS. We have added further comment on the rationale for this study in the Introduction (Page 4, para 2).

2. Related to the first comment, it would be valuable to clarify whether BMI at these early stages truly reflects adiposity. Given that BMI was developed for use in adults — who have reached their final height and whose body proportions remain stable — it is not immediately clear whether the same assumptions hold in infants and young children, whose body composition

and proportions change rapidly with age. Can loci identified in early infancy genuinely be described as "adiposity-related loci," or are they more accurately described as influencing variation in BMI, which may not directly equate to adiposity at this age? A discussion on this distinction would help readers better understand the biological relevance of the findings.

Response: BMI is considered to be a marker of adiposity in infants. This is supported by positive loading of Weight and negative loading of Length on prediction of Fat mass when measured by more accurate and reliable methods such as air-displacement plethysmography (Olga *et al.*, 2021) and total body electrical conductivity (Catalano et al 1995). By contrast, Fat-free mass shows positive loadings with both Weight and Length. Unfortunately, we know of no sufficiently large studies with measured infant body composition and GWAS data. We have added comment on this issue in the Discussion (Page 21, para 2).

3. A key strength of this study is the MoBa cohort, which offers a unique longitudinal design with repeated measurements very early in life — something that is relatively rare in childhood cohorts. This aspect adds significant value to the investigation of early-life growth and adiposity trajectories. However, I am less convinced of the added value of increasing statistical power for adiposity traits later in childhood by incorporating data from recalled phenotypes such as "comparative body size at age 10" (categorized as "plumper," "average," or "thinner") and age at menarche. Furthermore, while loci associated with age at menarche often overlap with adiposity loci, it would be helpful for the authors to discuss how the inclusion of these sex-specific variables might influence the interpretation of sex effects in their analyses.

Response: The validity of "comparative body size at age 10" for genetic associations has been well documented by ourselves and others, indicated by high genetic correlation (R_g=0.85 to 0.94) with objectively measured childhood BMI (Richardson et al.,2020; Kaisinger et al.,, 2023). This information is added to Supplementary Information (Page 10, Para 2).

We acknowledge that the inclusion of AAM, a female trait, could potentially favour female adiposity signals. However, our previous work reported substantial overlapping genetic architecture between sexes for puberty timing; 83% of AAM signals showed concordant effects on male puberty (Kentistou et al., 2024).

As described below (response to Q6), we find no genome-wide significant SNP*Sex interaction effects for our 624 lead SNPs in either MoBa (now added row 134-135) or UK Biobank (added on row 183-184), and only two at a weaker multiple test corrected threshold (with rs545608 at *SEC16B* and rs7749433 at *SIM1*. Both are stronger in girls but have concordant and at least nominally significant effects in boys. Both signals also show tendency to stronger effects on childhood BMI in MoBa age 8 years. Therefore, we conclude that inclusion of AAM has not led to the identification of sex-specific signals.

	Comparative size at age 10	Childhood BMI in MoBa-8Y
***SEC16B* rs545608**	**Boys: B=0.038, p=4.5×10^{-51}**	**Boys: 0.095, p=3×10^{-11}**
	**Girls: B=0.058, p=5.2×10^{-119}**	**Girls: 0.125, p=7×10^{-18}**
***SIM1* rs7749433**	**Boys = 0.007, p=4.0×10^{-3}**	**Boys: 0.023, p=0.1**
	**Girls = 0.021, p=1.7×10^{-21}**	**Girls: 0.052, p=0.0003**

4. Adiposity rebound and adiposity peak (age and BMI) are well-established metrics that provide valuable insights into early childhood growth patterns and, at least adiposity rebound is a known predictor of future obesity risk. Surprisingly, these metrics are not mentioned or discussed in the manuscript. It would strengthen the study to include a discussion on how the current findings relate to these established growth markers. For example, do the identified loci show association with any of these growth metrics?

Response: Thanks for this important comment. We have now related our findings to these two key measures in the discussion (page 20, para 2). However, it will require extensive methodological development, and it is far from trivial to estimate and formally analyse these parameters in our data, as well as performing validation in other cohorts. Hence, more formal investigations will be the focus of separate future work planned in collaboration with other studies.

5. While the study identifies many new loci and includes post-GWAS analyses, including comparisons with adult data, single cell data, CSTG, and more, it remains unclear what the key novel insights are in terms of understanding or predicting future obesity risk and what the key biological factors are that distinguish the regulation of childhood and adulthood body weight. The manuscript would benefit from a clearer articulation of how these findings advance our knowledge beyond what is already known from previous studies. Clarifying the translational significance would help underscore the broader impact of the study.

Response: We agree and have edited several places in the Discussion to highlight the key factors between child and adult adiposity and points of potential translational significance including:

- **i) Life-course dependent signals act through life-course dependent regulation', i.e. through differential actions on cell-specific regulators of gene expression (rather than via age-specific genes and pathways). The implications are that life-course dependent signals may still reveal intervention targets that are effective across the life-course (added to Page 19, para 3).**
- **ii) Stronger effects of leptin-melanocortin genes in childhood than in adults may predict stronger effects of drugs that target this pathway (leptin, setmelanotide) (added to Page 20, para 1).**
- **iii) Possible indirect implication of MAM-related memory & emotions (Page 19, para 2)**
- **iv) Our findings extend the parental conflict hypothesis to childhood adiposity (Page 21, para 1)**
- **v) Influence of established regulators of sex hormone secretion/action to early adiposity (GNRH1, FGFR1, ESR1) (Page 20, para 2)**
- **vi) MR shows relevance of early adiposity to later obesity-related disease, and hence supports the value of early intervention.**
- **vii) The discovery of variants with time-specific effects provides instruments for studies aimed at separating the effects of early from later life adiposity on later health (Page 20, para 3).**

6. Given the well-documented differences in growth patterns and changes in fat and lean mass

between boys and girls, it would be important to assess whether any sex-specific genetic effects were observed.

Response: Although biologically and clinically interesting, it is challenging to identify true sex-specific signals due to lower statistical power. We have now performed explorative analyses but do not find compelling evidence for individual SNPs with sex-specific effects:

- **None of the 152 lead SNPs detected in MoBa show evidence of SNP*Sex interaction (all P>0.01) and there was no overall inflation in test statistics.**
- **None of our 624 signals show evidence of SNP*Sex interaction at genome-wide significance, neither across MoBa timepoints nor with SAC10 in UK Biobank.**
- **Only two of the 624 SNPs (rs545608 at *SEC16B* and rs7749433 *at SIM1*) meet suggestive evidence for SNP*Sex interaction at the weaker Bonferroni corrected threshold (p<0.05/624). Both are stronger in girls but have concordant and at least nominally significant effects in boys. Both signals also show tendency to stronger effects on MoBa childhood BMI in girls than in boys (rs545608 $P_{interaction}$=0.06; rs7749433 $P_{interaction}$=0.099).**

	**Comparative size at age 10**	**Childhood BMI in MoBa**
***SEC16B* rs545608**	**Boys: B=0.038, $p=4.5\times10^{-51}$**	**Boys: 0.095, $p=3\times10^{-11}$**
	**Girls: B=0.058, $p=5.2\times10^{-119}$**	**Girls: 0.125, $p=7\times10^{-18}$**
***SIM1* rs7749433**	**Boys = 0.007, $p=4.0\times10^{-3}$**	**Boys: 0.023, p=0.1**
	**Girls = 0.021, $p=1.7\times10^{-21}$**	**Girls: 0.052, p=0.0003**

We have mentioned the overall lack of convincing sex-interaction tests to the Results (Line 134-135 and 183-184) and presented the single SNP interaction results in Supplementary Table 9.

Specific comments

ABSTRACT

L48: Split sample size across measured and recalled data, allowing readers to better assess quality of data.

Response: We now describe sample sizes split between those with objectively measured (N=62,276), and recalled childhood adiposity-related traits (N_{eff}=599,924).

L62: Add significance level for the “stronger adiposity associations” in adults.

Response: We have rephrased this statement to “Signals linked to the leptin-melanocortin pathway (*BSX*, *GNAS*, *LEPR* and *PCSK1*) and incretin-signalling genes (GIPR and GLP1R) show childhood-specific effects on adiposity”

RESULTS

L101: How many boys and girls?

Response: The proportion of girls and boys in MoBa varies slightly between the different timepoints and is described in Supplementary Table 1. We have now added the

frequency of girls corresponding to the time-point with the largest number of children which is 49.1% (now Page5, L116)

L114: Were there any signals reported in the previous paper that were not identified here? It seems that the previous paper reported 46 loci.

Response: As described above (Reviewer 1, comment #1), 36 of the 46 SNPs reported in Helgeland et al. (2022) are relevant for replication here (excluding 9 SNPs for birth weight, and 1 X-chromosome SNP). Of these 36 SNPs, 32 (the same variant or a proxy R2>0.5) show genome-wide significant ($P<5\times10^{-8}$) association with a childhood adiposity trait here. A further 3 SNPs show directionally consistent sub-genome-wide significant associations ($P<6\times10^{-5}$) with childhood adiposity here: PLCE1 (Helgeland SNP: rs1830890), LCORL (rs2610989), KLF14 (rs12672489). Only, RIN2 (rs148252705) shows a more modest but directionally consistent association in the current data ($P=4\times10^{-3}$). We have now described the overlap with previous studies in the discussion (page 529-535, sTable4 and 9).

L156-164: I have read through the methods, but it remains unclear to me how effect sizes of these difference GWAS (SAC10, AAM) can be compared as they do not seem to be on the same scale.

Response: Genomic structural equation models compare effects of each SNP on the included traits by generating standardised (P-value and effect direction-based) Z-scores (see scatterplots in Extended Data Figure 2).

Figure 3A: The effect sizes of the transient loci are--between 6 weeks and 5 years--about 2 to 5 times larger than that of the other clusters. How can this be explained?

Response: We believe that there are two possible reasons. Firstly, we have lower sample size and statistical power for Transient signals (in MoBa only) compared to signals with persisting effects (captured by the much larger genomic SEM). We could therefore detect only those Transient signals with relatively large effects (as can be seen from the plot below contrasting effect allele size in MoBa for variants discovered in MoBa vs in gSEM). Secondly, this might be a true biological feature possibly related to higher SNP-based heritability of BMI at this age. However, this remains speculative until larger studies have been performed to better characterize these effects across the full allelic spectrum.

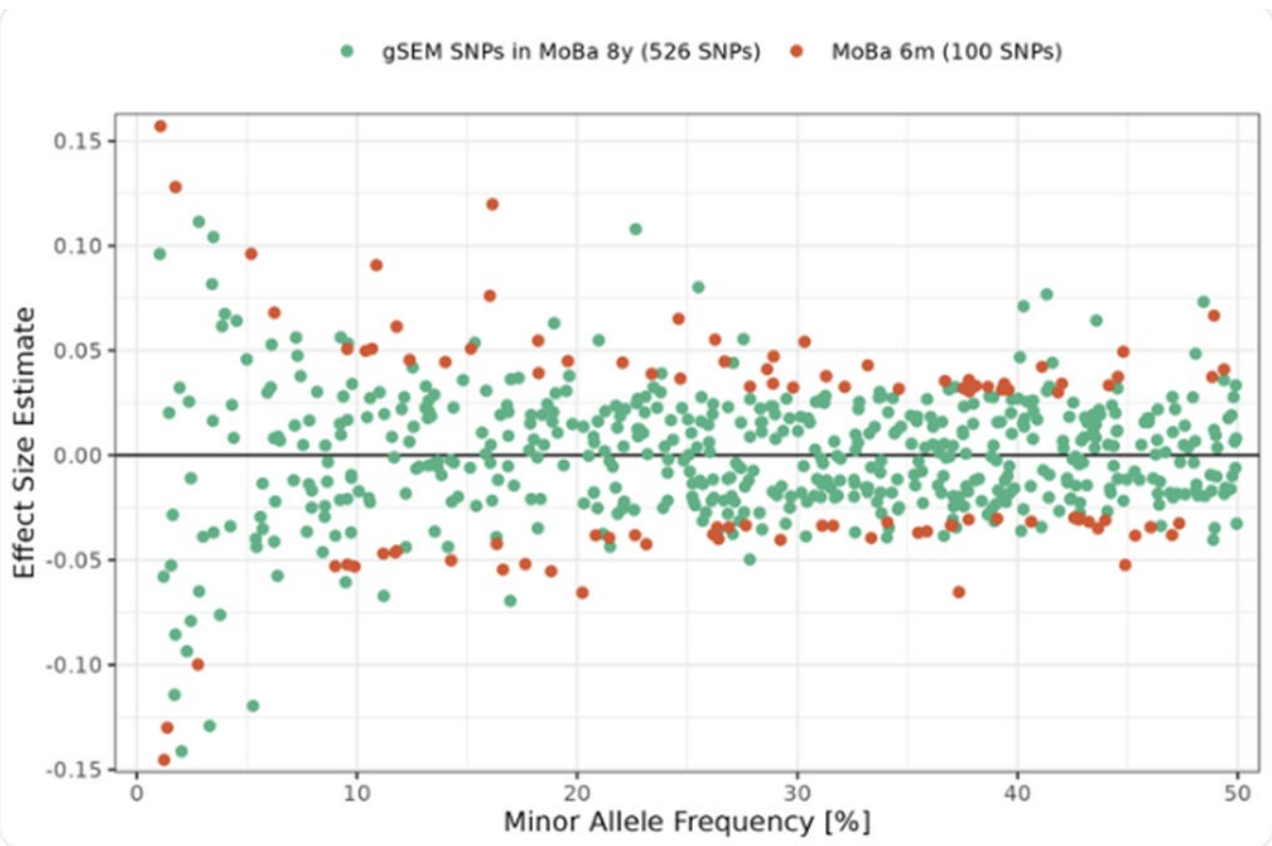

Figure 3B: Isn't it surprising that the variant in MC4R is a "late-rising" one, as rare variants in MC4R are known to cause early-onset obesity?

Figure 3B: Is it correct that rs77226405 an early rise locus? It does not seem to differ from the other MC4R locus, which is considered a late rising one.

Response: We identified 2 childhood adiposity signals linked to MC4R: 1 categorised as Early-rise (rs77226405), and 1 as Late-rise (rs476828). In Figure 3B, rs77226405 does appear to effect BMI earlier than rs476828 (note that ALSPAC study timepoints from age 10 onwards were not used for clustering). This suggests the presence of temporally-specific regulatory effects at this locus.

However, we acknowledge that our hard clustering approach was designed to facilitate cluster-level associations and may lead to imperfect classifications of individual signals. As we described in more detail below (Reviewer 3, Comment 2), while the Transient cluster separates well from the other clusters, there is weaker discrimination between Early-rise and Late-rise clusters. This is now discussed as a limitation (Page 21, para 2).

L207: Does that variant-to-gene mapping reveal new insights not seen for loci identified in adults?

Response: Results section (Page 14) highlights the 194 childhood GWAS signals not seen (or directionally discordant) for adult BMI and a number of notable genes linked to these signals.

L282: Clusters are not super clear. My impression was that the GLP1R locus was transient, where it is here described as an early-rising one.

Response: We apologise that GLP1R was incorrectly listed (now L302, Biological pathways) as having an Early-rising signal (it was correctly described as 'Transient' in Fig 3 and Table S10). This wording has been corrected.

L327: How do the observations in hypothalamic neurons for childhood-associated loci provide new insights beyond what has already been reported in adult data?

Response: While most associated cell groups were enriched in both adults and children, several appeared to be child specific. As mentioned in the Discussion, the location of the cell groups in the mammillary bodies may suggest a role for pathways related to recollective memory and emotional regulation in early adiposity (page 19, para 1).

L340: CALCR is only distantly part of the leptin-melanocortin pathway; it seems unfair to consider it at the same levels as LEPR and POMS.

Response: We agree and have removed *CALCR* from the lists of genes in the leptin-melanocortin pathway throughout the paper.

L344: GRP is not an incretin or incretin-related.

Response: We have corrected the description of GRP to "gut peptide".

DISCUSSION

L431-444: The inclusion of the AAM data to boost the power to identify more adiposity loci relies on the assumption that adiposity affects AAM. Can it be tested whether these variants indeed affect BMI in the first place?

Response: We did indeed test this question in our recent paper on AAM (Kentistou et al,., 2024)). There, we reported that 45% of AAM-lowering GWAS signals have a positive effect on weight during early childhood, before the age range for puberty onset, indicating that they impact AAM indirectly, via primary effects on early weight gain (see Figure below).

We have added a clearer description of the rationale for inclusion of AAM as a child adiposity-related trait in the Supplementary Information (Page 9 onwards).

Clustering of AAM-lowering GWAS signals by Early Weight Gain

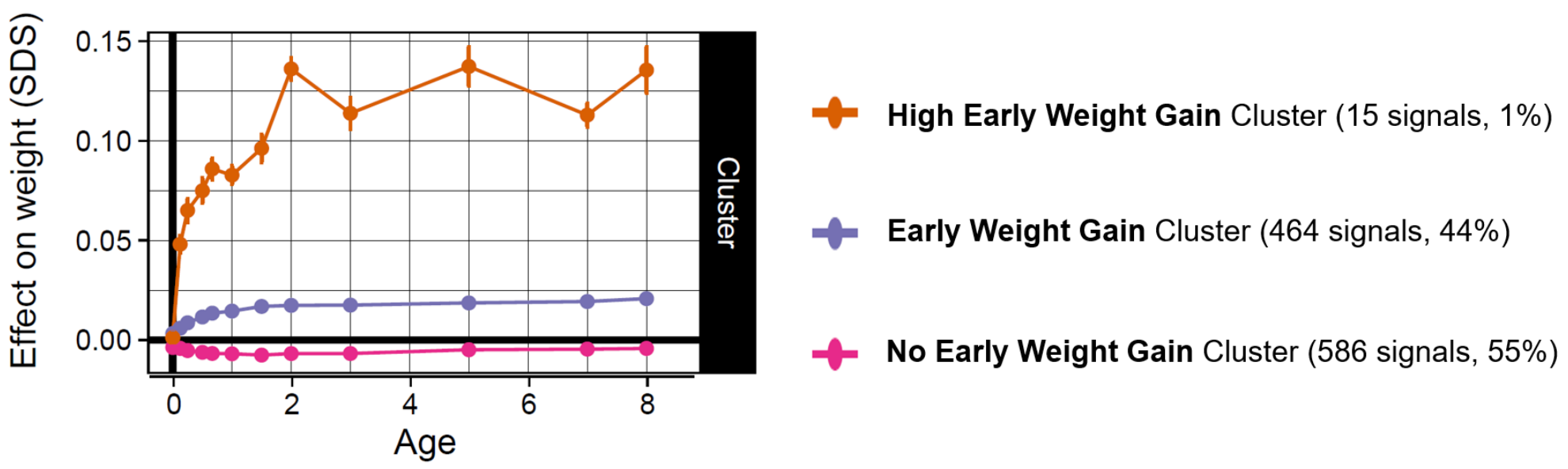

Early Weight data from MoBa

gSEM relies on genome-wide data (rather than GWAS significant AAM signals as we had reported previously, above), therefore we did not attempt to filter the genome-wide AAM summary statistics by their effects on early weight gain. Instead, we identified and excluded any gSEM-significant SNPs with stronger effects on AAM than other child adiposity traits (Q SNPs, Blue dots). By definition, the remaining included gSEM-significant SNPs had detectable effects on childhood adiposity.

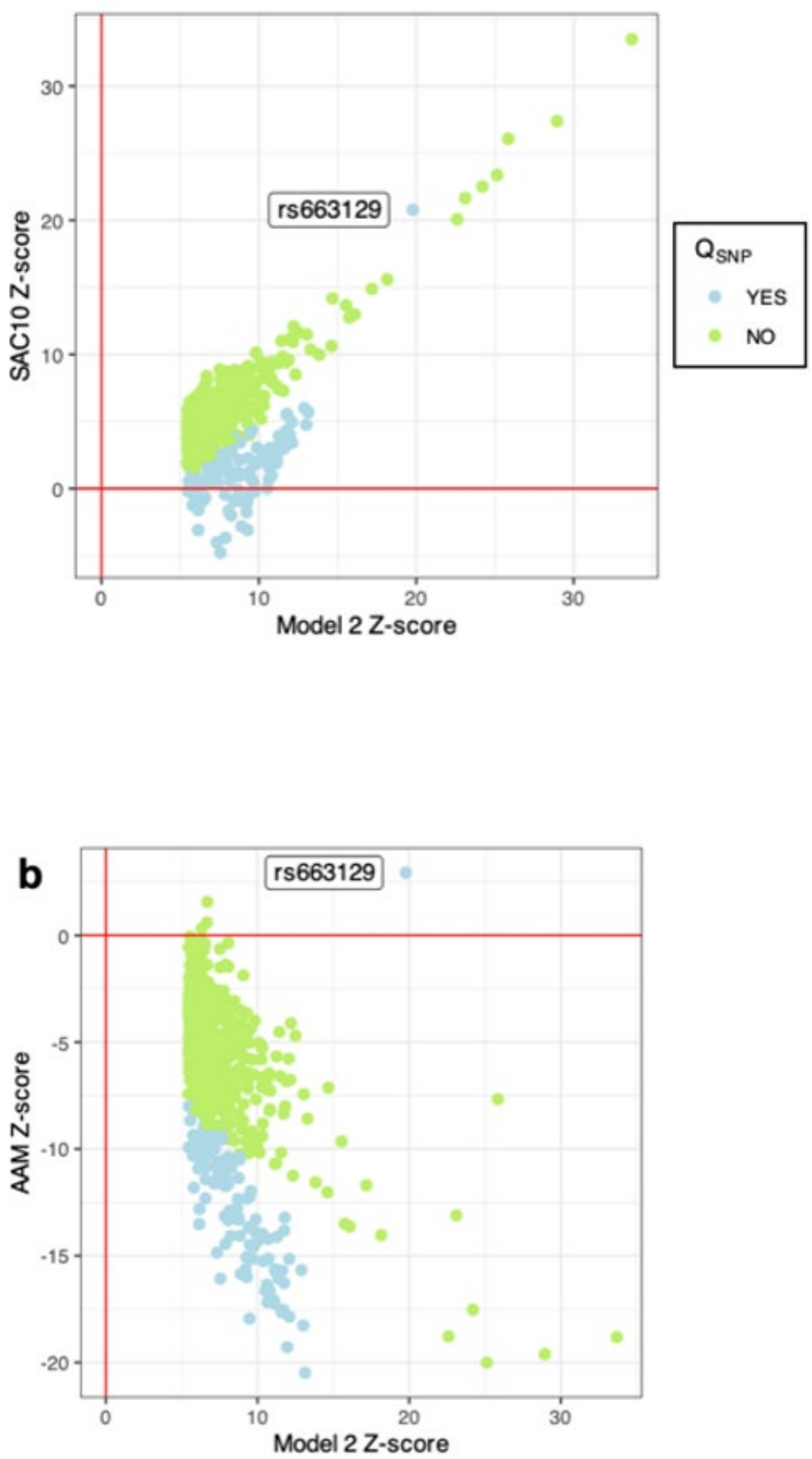

b
rs663129
SAC10 Z-score
AAM Z-score
Model 2 Z-score
Q_{SNP}
YES
NO

Reviewer #3 (Remarks to the Author):

This is a very interesting paper that sets a new benchmark in GWAS approaches to complex traits. It is the best example I can think of that shows how the genetic architecture of the "same" trait can be different between two life-stages. The fact that there are so many fascinating differences between the causes and consequences of excess weight in childhood – including protective effects on conditions such as breast cancer – and excess weight in adulthood, adds to the importance and interest. I have a few observations and comments, none of which are major concerns but may help the reader if addressed:

1. For readers familiar with GWAS and genetics of obesity and puberty timing, the genomic SEM approach will make some sense. It seems to me pretty convincing that the previous AAM GWASs were in effect GWAS for two traits – "pure" puberty and adiposity that leads to variation in pubertal timing. However, to some readers this may take some extra brain gymnastics and it may be worth spelling out even more. For example, looking at Supp Figure 2, I think it is clear that the homogenous gSEM variants are more specific for adiposity than AAM, but plots of AAM vs MOBA8 and AAM vs SAC10 could show a more striking separation? Similarly, it may help the reader if the authors could substantiate the statement in the results (from lines 147), about half the AAM signals acting through childhood adiposity. How does this predefined list of 50% match up to those that were GWAS significant in the genomic SEM model bit did not showing evidence of heterogeneity in the QSNP test? An extra supp table may be useful?

Response: We have added a clearer description of the rationale for AAM as a child adiposity-related trait in Supplementary Information (Page 10 onwards) with a figure to substantiate the statement that around half of AAM signals act through childhood adiposity.

We have added the suggested plots of AAM vs MOBA8 and AAM vs SAC10 to Extended Data Figure 2 to support the separation of genomic SEM signals with apparent AAM-predominant effects from those that also influence childhood adiposity.

As an external validation, we also now quantify the benefit of removing the 117 heterogenous variants (Q-SNPs) with disproportionately large impact on AAM compared to adiposity. After excluding the Q-SNPs the effect direction concordance with the independent child BMI EGG-discovery sample increases from 85% to 91%, and effect direction concordance with adult BMI (in UK Biobank) increases from 88% to 93%. These data are added to the Supplementary Information (Page 19).

A direct comparison between our previous and current categorisation of AAM signals is limited because the previous categorisation was based on early weight data from less than half of the current MoBa sample – hence we expect the current categorisation of gSEM signals to be more robust. Using the current larger MoBa data on early growth, we find that a further 65 of the previously reported AAM signals (reported as "pure puberty") now show positive effects on early BMI. These are now labelled (as 'not excluded' & 'AAM_nonweight' signals) in the new columns added to Supplementary Table 8.

2. Trajectory specific PRS and methods lines 662 - 673 – to what extent are these the result of dividing up continuous variation in how an overall PGS affects growth, and genuine heterogeneity? I do not see the stats in STs 10 and 11 that show the elbow effects or other

metrics that provide evidence of genuine differences. I have no doubt about the heterogenous effects between birth and childhood and between childhood and adulthood but forcing all variants into one of 4 groups may introduce some arbitrariness. The fact that the clusters are similar to an earlier analysis in MOBA and are “homogeneous” after splitting up does not provide much reassurance that these 4 clusters are really representing something separate as, respectively, the training and test data overlap and once a continuous variable is split up into 4 groups the effects will look homogenous and similar in other data. The potential arbitrariness of the assignment into clusters is shown in Figure 3 where the two PCSK1 signals are assigned to the same cluster but appear to swap over effect sizes between early and later childhood. In contrast, the 2 MC4R signals are assigned to different clusters but look quite similar in pattern with potential differences in when they appear explained by one having a weaker effect size?

Response: We acknowledge that our hard clustering approach was designed to facilitate cluster-level associations, and this may lead to some imperfect classifications of individual signals.

It is highly reassuring that the unsupervised clustering model used here shows consistent patterns both to our previous report and in the independent ALSPAC cohort (see Fig below). Helgeland et al,. 2022 used a crude model based on discovery data only for MoBa BMI (birth to age 8 years). Our current unsupervised model is based on a 2x larger MoBa dataset and includes gSEM signals, derived using much greater power to detect signals for later childhood BMI. In both these approaches, the clusters divide following the same sequence: 1st Transient, then Birth-related, and then Late-rise and Early-growth clusters.

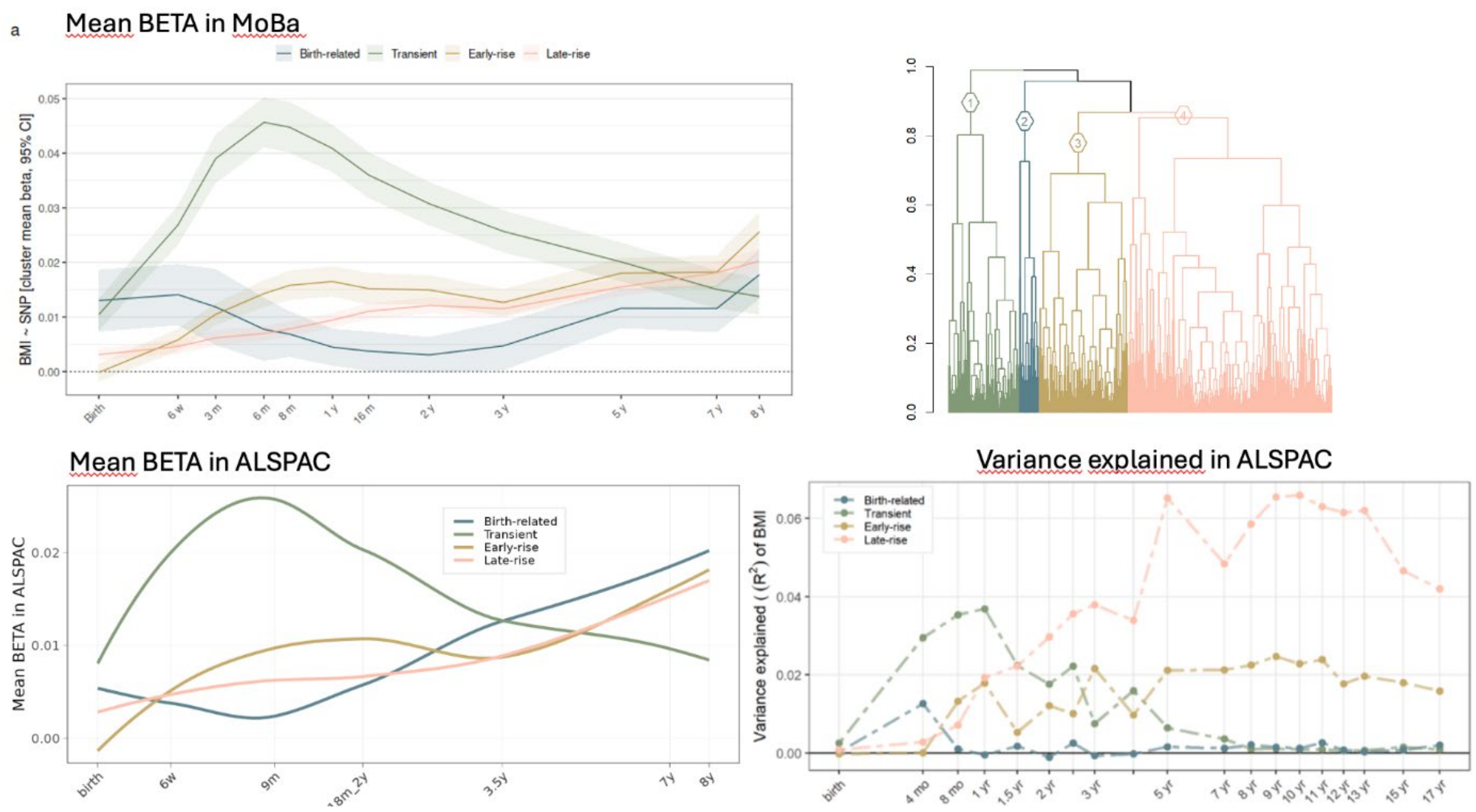

We now provide performance indicators for the current clustering approach:

- **The Transient cluster shows the most robust separation from the others (average stability = 0.89 for the 2-cluster model; and the Transient cluster has a stability of 0.79 in the 4-cluster model).**

- **Average stability decreases to 0.73 with 3 clusters (where the "birth-cluster" appears) and 0.51 with 4 clusters (which separates Early-rise from Late-rise).**

Therefore, there is greater confidence in assigning individual signals to the Transient cluster, and greater propensity for imperfect classifications of individual signals particularly between the Early-rise and Late-rise clusters. This issue is now mentioned in the Results section (Line 210-211 and 215-216) and is also now discussed as a limitation (Page 21, para 2).

N clusters	Average silhouette width	Average stability
2	0.56	0.89
3	0.29	0.73
4	0.16	0.51
5	0.20	0.48
6	0.16	0.43

Cluster performance statistics per cluster size.

Cluster/trajectory	Size	Silhouette width	Stability
Birth-related	32	0.22	0.15
Transient	115	0.62	0.79
Early-rise	145	0.02	0.25
Late-rise	330	0.06	0.56

Cluster-specific performance statistics for the four cluster model. Now added as Supplementary Table 11.

3. There appear to be signals in same gene/locus doing different things (although see point 2 maybe this is chance?). If the signals are non-coding, it is easier to imagine how that could happen – different regulatory elements being affected at different times of life. For coding variants, this is harder to imagine unless obvious differences in the location of the variants in relation to different transcripts expressed at different times of life. Or maybe just an interesting mystery and worth mentioning in the limitations?

Response: We also find this very interesting. Despite the above limitations of assigning individual signals to trajectories (particularly between Early-rise and Late-rise trajectories), the presence of differing age-specific signals at the same gene is also supported our finding of both childhood-specific and adult BMI-associated signals at the same gene. For example, at *LEPR* we identify 4 signals, only 1 of which is associated with adult BMI. Together with our hypothalamus cell population data, our findings support the hypothesis of age-specific regulators of common genes and pathways that regulate adiposity. We have now commented on these aspects in the discussion, (page 19 para 1 and 2)

Minor points

1. Abstract. Although the word “upto” technically covers it, a large proportion of the power of the GWAS discovery component to the study comes from the cross-sectional UK biobank age 10 and AAM data. So “longitudinal study of upto 599,000 people” is a little misleading. Maybe “including the largest genetic study of longitudinal...” - however see next point, there is a recently published study the authors should place their study in this new context.

Response: In the abstract we now report separately the sample size with objective BMI data and the effective sample size from the genomic-SEM model.

2. Introduction line 79. Given the Richardson et al SAC and AAM GWAS data are “childhood adiposity related” traits critical to the study, and there is a cross-sectional BMI in childhood GWAS of 84k people, very recently published (Trans-ancestry genome-wide association study of childhood body mass index identifies novel loci and age-specific effects - PubMed) maybe reword "However, previous GWAS of directly measured BMI in childhood have been limited to a GWAS of single time point measures..."

Response: We now describe this recent meta-analysis of child BMI and have reworded the introduction (Page 4, para 2).

3. Introduction. Probably worth a more explicit sentence on the Richardson et al study that did show very nicely the differences between childhood and adult BMI on major outcomes.

Response: We now provide more detail of the findings of Richardson et al in the Introduction (Page 4, para 2).

4. Results line 116. Of the 152 signals, how many overlap with GIANT 2018 data?

Response: Of the 152 MoBa child BMI signals, 26 were associated with adult BMI (at $P<5\times10^{-8}$) in the 2018 GIANT data. This is added to line 322-323 (Adult-child comparison).

5. Methods line 491. There were some measures reported by parents rather than measured? This sentence does not flow well from the preceding one.

Response: We have clarified this description (now Line 539). In MoBa, children were measured at routine checkups by trained nurses. These values were recorded in parent-held booklets and parents were then asked to transcribe these data into the study questionnaires.

6. Line 562 an apostrophe missing from SNPs (sorry)

Response: This has now been added.

7. Line 423 comparing WES based signals and the striking difference between the 8 discovered in adults and 6 in children that have little overlap. It could be more helpful to reader to talk about differences in effect sizes than whether or not discovered at a threshold. I think many of the childhood ones will have effects in adults, but just weaker?

Response: We do present a comparison of the associations between children and adults in the results section (line 403-408 and Figure 7b). The effect sizes are presented in Figure 7B, but are not easily comparable between measured adult BMI and the categorical retrospectively recalled SAC10. Nevertheless, the lower effects and much weaker p-values

(despite same sample size) suggest that effects are attenuated in adulthood for the variants detected in childhood.

8. Line 553 A Heywood case? worth a few more words to explain that

Response: We have clarified that a Heywood case occurs when the standardised factor loading for an input trait exceeds 1 (now Line 600).

9. Discussion opening line. The signals and effective sample size are heavily driven by the age at 10 size in UK Biobank (looking at SF2) and the AAM GWAS. Given both of these GWAS are previously published (the first in the Richardson study), it is probably worth toning down the "by far the largest ever" wording.

Response: We have removed "by far" from this sentence.

10. Discussion paragraph starting on line 406. I am not sure the second sentence follows from the first? Perhaps some more nuanced wording needed?

Response: We have rephrased this text to clarify that these two findings both support the apparent differential regulation of child and adult adiposity.

References:

Catalano, P.M. *et al.* (1995) 'Anthropometric estimation of neonatal body composition', *American Journal of Obstetrics and Gynecology*, 173(4), pp. 1176–1181. Available at: https://doi.org/10.1016/0002-9378(95)91348-3.

Ekelund, U. *et al.* (2006) 'Upward weight percentile crossing in infancy and early childhood independently predicts fat mass in young adults: the Stockholm Weight Development Study (SWEDES)', *The American Journal of Clinical Nutrition*, 83(2), pp. 324–330. Available at: https://doi.org/10.1093/ajcn/83.2.324.

Helgeland, Ø. *et al.* (2022) 'Characterization of the genetic architecture of infant and early childhood body mass index', *Nature Metabolism*, 4(3), pp. 344–358. Available at: https://doi.org/10.1038/s42255-022-00549-1.

Kaisinger, L.R. *et al.* (2023) 'Large-scale exome sequence analysis identifies sex- and age-specific determinants of obesity', *Cell Genomics*, 3(8), p. 100362. Available at: https://doi.org/10.1016/j.xgen.2023.100362.

Kentistou, K.A. *et al.* (2024) 'Understanding the genetic complexity of puberty timing across the allele frequency spectrum', *Nature Genetics*, 56(7), pp. 1397–1411. Available at: https://doi.org/10.1038/s41588-024-01798-4.

Olga, L. *et al.* (2021) 'Anthropometry-based prediction of body composition in early infancy compared to air-displacement plethysmography', *Pediatric obesity*, 16(11), p. e12818. Available at: https://doi.org/10.1111/ijpo.12818.

Paul, I.M. *et al.* (2018) 'Effect of a Responsive Parenting Educational Intervention on Childhood Weight Outcomes at 3 Years of Age: The INSIGHT Randomized Clinical Trial', *JAMA*, 320(5), pp. 461–468. Available at: https://doi.org/10.1001/jama.2018.9432.

Paul, I.M. *et al.* (2025) 'Long-Term Effects of a Responsive Parenting Intervention on Child Weight Outcomes Through Age 9 Years: The INSIGHT Randomized Clinical Trial', *JAMA pediatrics*, 179(8), pp. 827–835. Available at: https://doi.org/10.1001/jamapediatrics.2024.6897.

Richardson, T.G. *et al.* (2020) 'Use of genetic variation to separate the effects of early and later life adiposity on disease risk: mendelian randomisation study', *BMJ*, 369, p. m1203. Available at: https://doi.org/10.1136/bmj.m1203.

Savage, J.S. *et al.* (2016) 'Effect of the INSIGHT Responsive Parenting Intervention on Rapid Infant Weight Gain and Overweight Status at Age 1 Year: A Randomized Clinical Trial', *JAMA pediatrics*, 170(8), pp. 742–749. Available at: https://doi.org/10.1001/jamapediatrics.2016.0445.

Vogelezang, S. *et al.* (2020) 'Novel loci for childhood body mass index and shared heritability with adult cardiometabolic traits', *PLOS Genetics*. Edited by G.P. Copenhaver, 16(10), p. e1008718. Available at: https://doi.org/10.1371/journal.pgen.1008718.

Cover letter

Kyle Vogan, PhD
Senior Editor
Nature Genetics

Dear Editor,
We thank the editor and reviewers for the positive assessment of our work and for giving us the opportunity to submit a revised manuscript.

Please, find below our point-by-point response to all of the comments and questions raised by the reviewers. We have added several new analyses, including an extensive phewas of 79 traits in the UK-biobank, and new mendelian randomization analyses to better illustrate the relationship between childhood BMI and later disease risk as asked for by the reviewers. We have changed to a two-cluster model instead of the four-cluster model as suggested by reviewer 3 and adapted the downstream analyses accordingly. Changes in the manuscript are highlighted in red.

We are grateful to all reviewers for their comments and suggestions, and believe that the manuscript has improved considerably due to the review process. We hope that the manuscript can now be considered for publication in Nature Genetics, and we look forward to hearing from you.

On behalf of all authors,

Stefan Johansson PhD
Professor and Head of the Diabetes Research Groups
Department for Clinical Science, University of Bergen
and
Clinical Laboratory Geneticist at
The Department for Medical Genetics
Haukeland University Hospital
stefan.johansson@uib.no

Responses to the Reviewers' Comments:

Reviewer #1 (Remarks to the Author):

The authors have made a concerted effort to address the many concerns addressed by all the reviewers, and as such have done much to improve the quality of the manuscript. I appreciate the additional clarity the authors brought to the work with respect to replication of previous MoBa results and also from unrelated data. These comparisons are much more convincing now. The added information with respect to the gSEM in both the main and supplementary text are also helpful in convincing the reader that this approach was meaningful and appropriate. Despite the considerable added information, I do think two main areas are preventing the paper from reaching its full potential.

A few comments:

1) Multiple reviewers noted the valuable missing piece of understanding the long-term impact or significance of childhood obesity, particularly as it relates to the genetic contribution. Exploring this more broadly than the extremely limited set of cardiometabolic traits currently mentioned would dramatically enhance the value of the paper. While I do appreciate the potential scale of additional work, and I could be convinced there is more value in examining these relationships in an entirely different paper, suggesting they are beyond relevance for the paper is not convincing.

RESPONSE We appreciate the reviewer's concerns and have now devoted a new results section to this topic (rows 320-345). We first performed a 1-sample 'Phewas' in the UK Biobank to explore the potential relevance of our 'Transient' and 'Childhood Persistent' adiposity signals on later diseases, i.e. the 78 ICD10 codes with at least 20,000 cases in UK Biobank. The results are shown in Supplementary Table 20 and Extended Data Figure 7 and show that:

- Childhood Persistent Adiposity signals collectively associate with higher risks of a wide range of cardiometabolic outcomes. When controlling for adult BMI, most of these associations attenuate towards the null (notably: non-insulin-dependent diabetes and chronic ischaemic heart disease). As previously reported by others, breast cancer is an exception by showing a childhood adiposity-specific (protective) association (Ref 14; Richardson et al, 2020).
- Transient Adiposity signals collectively do not appear to increase the risk of any of the tested later disease outcomes. Conversely, they appear to be protective for later non-insulin-dependent diabetes and essential hypertension. When controlling for adult BMI, these protective associations modestly strengthen.

To confirm and extend these findings, we additionally explored the Phewas associations with T2D and Hypertension using a 2-sample Mendelian randomization

approach (Supplementary Table 21 and Extended Data Figure 7), similar to that in (Ref 14; Richardson et al, 2020), modelling as the exposures: i) BMI at 12 months (at the infant adiposity peak) and ii) Childhood Adiposity (signals from our gSEM model). The main results from these analyses are:

- Univariable MR – confirms that pre-pubertal Childhood Adiposity is associated with higher risk of T2D. MVMR - confirms that this association is fully attenuated when controlling for adult BMI.
- Univariable MR – shows that BMI at 12 months has no association with T2D. MVMR – shows that a protective effect of early adiposity on T2D appears when controlling for adult BMI.

In summary, we find no evidence for a later adverse effect of early adiposity (either transient or BMI at 12 months). Conversely, a transiently elevated infant adiposity that subsequently resolves may have protective effects against later T2D and hypertension. For Childhood Adiposity, we find adverse effects on a wide range of cardiometabolic outcomes, which appear fully mediated by its persisting effects on adult BMI. These results are commented in the discussion (rows 510-514) and described in detail in a new section of the manuscript that replaces the previous limited set of MR-analyses that were performed in prior versions of the manuscript (new suppl Tables 20, 21 and Extended Data Figure 7 - also shown below).

Extended Data Figure 7 | Disease-wide association analysis and mendelian randomization analysis. a) Odds ratios of selected traits from disease-wide association analysis and b) inverse-variance weighted odds ratios from mendelian randomization of type 2 diabetes and hypertension. Further data can be found in **Supplementary tables 20** and **21**.

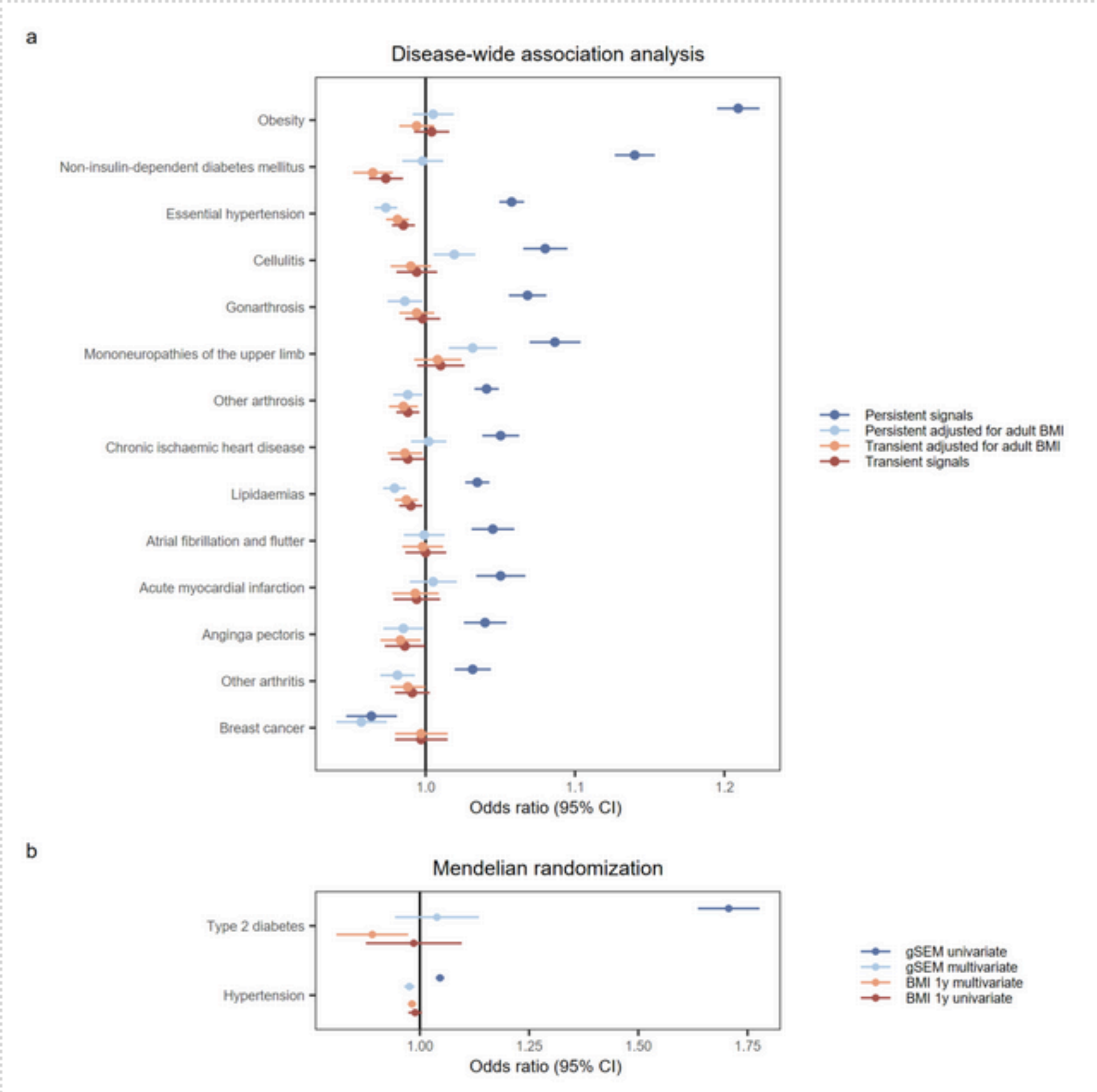

2) *It appears, through the review process, the authors feel their key discovery in this work is the apparent differential regulation of core obesity genes/pathways between early life obesity and adult obesity. The authors do make this broad conclusion more clear, but I both feel this "key finding" is under-represented in the text and that the data supporting this conclusion, as opposed to other alternate hypotheses, could be shown more clearly and discussed in more detail. It is mostly presented as conjecture in the discussion. If well supported, I do think this is a particularly interesting and important conclusion from this work, and probably should be mentioned in the abstract as well.*

RESPONSE In an attempt to substantiate the points made in the discussion, we have now undertaken several further analyses to distinguish between early life and adult adiposity genes and signals and to quantify the extent of shared and non-shared child and adult adiposity signals.

We now present a new extended gene-centric analysis (rows 247-254, and new sTable 15): that shows that 217 of our 434 high-confidence childhood adiposity genes have no association with adult BMI, i.e. no genome-wide significant signal for adult BMI within 500 kb and r2>0.05 of the childhood signals (e.g. *GLP1R, BSX, GNAS, BBS9*). A further 27 genes show more complex pattern with both child-specific and shared childhood/adult adiposity signals, indicating that some common adiposity genes are differentially regulated between early and later life (e.g, *LEPR, PCSK2, NEGR, DLK*1 and *CALCR*). The remaining 190 genes have signals associated with both childhood adiposity and adult BMI.

We also performed a biological pathway enrichment for adult BMI GWAS signals in order to distinguish those pathways specifically enriched for childhood adiposity associations from those that are shared with adult BMI (rows 313-319). This finds 60 individual pathways and 6 pathway groups specifically enriched for childhood adiposity signals, these pathway groups included "*cellular response to hormone stimulus*", "*regulation of axon regeneration*", and "*response to monosaccharide.*

Reviewer #2 (Remarks to the Author):

A few concerns/comments remain:

Response to comment 1(i.e. from previous revission):
In their response, the authors focus on weight gain, which was not examined in the current study. Moreover, the cited papers report evidence of weight gain only within the first year of life, with no data or findings presented for the subsequent early years. As such, I still find there to be limited clarity around what cross-sectional BMI at these very early ages actually represents, and whether it can be reliably defined as a measure of adiposity.

The proposed PheWAS analysis could help clarify this, and I would suggest including at least a table to provide further insight. In my view, understanding what early BMI truly reflects is central to the study's aims.

Response: As described in response to Reviewer 1, Comment #1, we have now performed an extensive Phewas on the 78 most prevalent disorders in the UK Biobank on the transient and childhood persistent BMI clusters, with and without controlling for adult BMI. We then followed up these findings with MVMR to test whether the early life adiposity (around age 1 year) and/or childhood adiposity (gSEM results) have independent effects on disease risk or whether they are mediated through later life adiposity. These results show that high early life adiposity/BMI does not seem to have adverse consequences on adult health, but rather putative protective effects on T2D and hypertension, particularly when controlling for the effects of adult life adiposity.

These data are now presented as Supplementary Data 20, and Extended Data Figure 6 and discussion rows 510-514.

Regarding the validity of BMI to proxy adiposity in early childhood. BMI is obviously more easily available than more precise measures of adiposity and is still widely used in pediatrics as it shows relatively high correlation with direct measures of adiposity in adults, adolescents and school-aged children (correlation coefficients of 0.79–0.92). The use of BMI to proxy adiposity in infancy is more uncertain, but studies report that zBMI correlates well with fat mass and fat mass index (FMI), particularly at around ages 4 to 7 months (fat mass r_s=0.72–0.76; FMI r_s=0.75–0.79) (Bell et al, 2018, PMID: 29551311), with similar results in (Perng et al 2017, PMID: 28659296) and to lesser degree with lean mass. We therefore argue that BMI is a valid measure that allows us to perform large scale GWASs to identify important regulators of adiposity also in early childhood. It is also reassuring that we find that several of the strongest signals in infancy are located near established obesity genes and/or drug targets, such as *LEPR*, *ADCY3* and *GLP1R*.

Reviewer´s response to comment 2 (from first revision):
The Olga et al. study does not examine BMI, nor does the Catalano et al. study. Both focus on other anthropometric traits, but not BMI specifically. Similarly, the authors' response does not directly address BMI, instead concentrating on weight and length. To strengthen the argument, it would be helpful to include more appropriate references that specifically quantify the relationship between BMI and adiposity in very early life.
Response: We have removed these references and now instead cite references that report the validity of BMI as a measure of adiposity in infancy, as in our response to your Comment #1 above (Bell et al, 2018, PMID: 29551311 and Perng et al 2017, PMID: 28659296). These studies support the validity of zBMI in early life GWASs:

This has now been addressed in the discussion (rows 542-546): *"We assumed BMI in infancy to be a marker of adiposity. This is supported by studies of fat mass measured by more accurate methods such as air-displacement plethysmography[49] and total body electrical conductivity[50] (correlation rs= 0.70-0.76 between zBMI and fat mass in infancy age 4 to 7 months) and we know of no sufficiently large studies with measured infant body composition and GWAS data."*

Response to comment for L114:
Are there any insights into the 10 loci (out of 46, or approximately 20%) reported by Helgeland et al. that were not replicated in the current study?

Response: Only 36 of the 46 SNPs reported in Helgeland et al. (2022) are relevant for replication in the current study and these showed good replication here. The remaining 10 non-relevant SNPs are:

I) One SNP located on chromosome X which is unavailable in the current data

Ii) Nine SNPs were identified as associated with BMI at birth by Helgeland (2022). BMI at birth was not included in the current GWAS. However, a comparissons with that data from the large Birth weight Meta-GWAS by Warrington et al 2019, show that all of these nine SNPs show genome-wide significant associations with birth weight (all P<5x10-8).

Reviewer #3 (Remarks to the Author):

Many thanks for the clarifications. The metrics on clustering are a very useful addition, but really point to, at best, evidence that signals can be split into two groups, not four (based on the silhouette metrics that relate to the average distance of individual datapoints to all those in the same cluster compared to all those in different clusters). I know it will be a little painful to reformat these results, but it would be a much better representation of the evidence to talk about 2 clusters. By splitting into 4, there is a risk of over interpretation by readers and all those researchers wishing to follow up what is otherwise a fascinating paper.

Response: We now focus the analysis on the two-cluster model and present the childhood adiposity signals (and all downstream analyses) split into 2 clusters: the 'Transient' adiposity cluster remains the same as previously, and the remaining 3 clusters (Early-rise, Late-rise, and Birth) now form a single 'Childhood Persistent' adiposity cluster. The four-cluster model is now mentioned as a plausible sub-categorization that may potentially be distinguishable with larger studies and more refined methods. We make clear that the 2-cluster model is the best fit. In addition to providing more robust distinction between trajectories, having fewer trajectories has increased the power of downstream analyses for trajectory-specific biological pathway enrichment.

These data are now presented in the results section row 202-212, Figure 3, Extended Figures 4-5, sTable 11-12, and all down-stream analyses refer to the 2-cluster model.

Limitations with the clustering approach are discussed in row 535-540.

"
